# Comprehension of the Synergistic Effect between m&t-BiVO_4_/TiO_2_-NTAs Nano-Heterostructures and Oxygen Vacancy for Elevated Charge Transfer and Enhanced Photoelectrochemical Performances

**DOI:** 10.3390/nano12224042

**Published:** 2022-11-17

**Authors:** Zhufeng Shao, Jianyong Cheng, Yonglong Zhang, Yajing Peng, Libin Shi, Min Zhong

**Affiliations:** 1 College of Physical Science and Technology, Bohai University, Jinzhou 121000, China; 2 College of Chemistry and Materials Engineering, Bohai University, Jinzhou 121000, China

**Keywords:** m&t-BiVO_4_/TiO_2_-NTAs nanoheterojunctions, oxygen vacancy defects, charge transfer, photodegradation, biosensing and gas-sensing application

## Abstract

Through the utilization of a facile procedure combined with anodization and hydrothermal synthesis, highly ordered alignment TiO_2_ nanotube arrays (TiO_2_-NTAs) were decorated with BiVO_4_ with distinctive crystallization phases of monoclinic scheelite (m-BiVO_4_) and tetragonal zircon (t-BiVO_4_), favorably constructing different molar ratios and concentrations of oxygen vacancies (V_o_) for m&t-BiVO_4_/TiO_2_-NTAs heterostructured nanohybrids. Simultaneously, the m&t-BiVO_4_/TiO_2_-NTAs nanocomposites significantly promoted photoelectrochemical (PEC) activity, tested under UV–visible light irradiation, through photocurrent density testing and electrochemical impedance spectra, which were derived from the positive synergistic effect between nanohetero-interfaces and V_o_ defects induced energetic charge transfer (CT). In addition, a proposed self-consistent interfacial CT mechanism and a convincing quantitative dynamic process (i.e., rate constant of CT) for m&t-BiVO_4_/TiO_2_-NTAs nanoheterojunctions are supported by time-resolved photoluminescence and nanosecond time-resolved transient photoluminescence spectra, respectively. Based on the scheme, the m&t-BiVO_4_/TiO_2_-NTAs-10 nanohybrids exhibited a photodegradation rate of 97% toward degradation of methyl orange irradiated by UV–visible light, 1.14- and 1.04-fold that of m&t-BiVO_4_/TiO_2_-NTAs-5 and m&t-BiVO_4_/TiO_2_-NTAs-20, respectively. Furthermore, the m&t-BiVO_4_/TiO_2_-NTAs-10 nanohybrids showed excellent PEC biosensing performance with a detection limit of 2.6 μM and a sensitivity of 960 mA cm^−2^ M^−1^ for the detection of glutathione. Additionally, the gas-sensing performance of m&t-BiVO_4_/TiO_2_-NTAs-10 is distinctly superior to that of m&t-BiVO_4_/TiO_2_-NTAs-5 and m&t-BiVO_4_/TiO_2_-NTAs-20 in terms of sensitivity and response speed.

## 1. Introduction

With the continuous development of globalization and the spread of infectious viruses (such as COVID-19) encroaching on human health, the sustainable utilization of energy and the protection of the natural environment have become issues of substantial concern worldwide. A promising avenue is photoelectrochemicals (PEC), which, due to the pioneering work of Fujishima and Honda, have received a great deal of attention for a variety of potential applications, including water-splitting hydrogen generation, rechargeable solar cells, photocatalytic fuel cells, organic pollutant photodegradation, and biosensing [1,2,3]. TiO_2_ is credited as among the most promising and well-documented PEC materials, as it possesses an appropriate energy-band position towards the redox reaction of water splitting, has excellent chemical stability, is abundantly available, and is environmentally friendly. At present, powder suspensions and thin films are the two representative types of TiO_2_-related photocatalysts [4]. Notably, powdered TiO_2_-based PEC materials have been adopted because they have a fully available surface area, lower requirements in terms of cleaning, and they can be conveniently manipulated [5]. Nevertheless, the practical application of TiO_2_-related powdered photocatalysts is still under restrictions due to the poor PEC performance, low reusability, and low recyclability [6]. Compared with the PEC nanomaterials of particulates, thin-film TiO_2_-related photocatalysts have gained more attention owing to the following reasons: (i) with valid and appropriate light absorption, abundant free electrons are generated; (ii) the thin-film form demonstrates maximum activity, with the activity up to an order of magnitude higher compared with the powder form; (iii) the operating cost and recycling reuse of thin-film-based panels are expected to be substantially lower compared with powder-like samples without mechanical stirring; (iv) more efficient electron transfer occurs in thin-film PEC nanosystems through the underlying conductive layer, and therefore, the thin-film system can substantially improve the PEC activity compared with powder suspension processes; (v) they are well suited to efficient large-scale applications [7,8]. Furthermore, the self-organized TiO_2_ nanotube array films (NTAs) prepared by the Ti foil anodic oxidation process and vertically oriented on Ti-metal substrates are outstanding nanoscale thin-film architectures for boosting the PEC and biosensing performances [9,10]. TiO_2_ NTAs with highly ordered nanoporous surfaces possess unique characteristics, which are summarized as follows: (i) an enhanced active adsorption area for redox target compounds [11]; (ii) ordered-array architectures, which not only enable charge transfer (CT) along the axial direction, but also enable the segregation of the photoexcited charge carriers [12]; (iii) band modification to improve the light absorption and reduce the charge recombination [13]. Although the profitable properties of TiO_2_ NTAs are evident, the intrinsic features of TiO_2_ remain, which mainly include the UV-activated wide band-gap energy (E_g_~3.2 eV), and the rapid charge recombination rate that results in a sluggish rate of charge separation [14], which inherently conflicts with the aim of PEC-related practical applications under visible-light irradiation. To circumvent the abovementioned obstacles, researchers have demonstrated that doping with metals (e.g., Au, Ag, and Cu) or nonmetals (e.g., C, N, and S) is a valid route for elevating the visible-light-harvesting capacity of TiO_2_ NTAs [15]. Metallic elements, when deposited on TiO_2_ NTAs, can induce a suitable band-gap shift and act as light gatherers, prolongating the wavelength absorption scope and enhancing the PEC activity in the visible-light region. Nevertheless, the method is hampered by several drawbacks, as noble-metal nanoparticles (NPs) are quite toxic in nature, the reaction setup is costly and cumbersome, and photocorrosion is inevitable during the PEC process. Likewise, using nonmetal ions instead of metals to dope TiO_2_-NTAs photoanode materials is an alternative viable approach for exploring visible-light-active photocatalysts. Nonmetallic element doping could introduce mid-gap energy levels above the valence band (VB) of TiO_2_ NTAs and act as a trapping center for the photoexcited electrons, which achieve the expected purposes for the photoresponse of the narrow band gap and suppress the recombination of photogenerated species. The decrease in the electronegativity of PEC materials that leads to a reduced PEC-related capacity due to the introduction of new energy states is an unavoidable issue [16]. Considering the trend of practical application, visible-light-active PEC nanosystems with striking CT abilities are more advantageous because a smaller percentage (5%) of the solar spectrum is emitted in the UV region. Within this frame, the construction of TiO_2_-NTAs-based nano-heterojunctions not only substantially broadens the light-harvesting window, but it can also help increase the speed of the charge carrier separation. 

Bismuth vanadate (BiVO_4_) is an intrinsic n-type direct band-gap ternary oxide semiconductor, and it has been proposed as a promising alternative to visible-light-active PEC materials owing to its high stability, nontoxicity, and appropriate band position [17]. The PEC performances of BiVO_4_ are intensely affected by the prepared morphology and crystallographic structure. BiVO_4_ appears in three main crystalline phases based on different synthesized methods: monoclinic scheelite (ms-BiVO_4_), tetragonal scheelite (ts-BiVO_4_), and tetragonal zircon (tz-BiVO_4_), with E_g_ values of 2.4 eV, 2.4 eV, and 2.9 eV [18,19], respectively. Among them, state-of-the-art ms-BiVO_4_ is the most stable and has the best photocatalytic activity, delivering a remarkable stability above 1000 h. The theoretical photocurrent density was 7.5 mA cm^−2^ with a solar-to-hydrogen conversion efficiency of 9.2% under AM 1.5 G illumination [20], which is mainly attributed to the transition from the VB to the conduction band (CB) caused by the orbital mixing of Bi 6s and O 2p, which leads to the narrowed band gap and the sufficient oxidation potential of the VB (ca. +2.79 eV vs. NHE) to oxidize various organic compounds [21]. Moreover, ts-BiVO_4_ has similar crystal and energy structures to those of ms-BiVO_4_, which have rarely been studied, while the crystal phase of tz-BiVO_4_ exhibits the lowest photocatalytic performance owing to its wide bandgap, which restricts the widespread photodegradation and water-splitting application in visible-light regions. Based on conclusive experiments [22], researchers have verified that an inferior carrier mobility (0.044 cm^2^ V^−1^ s^−1^), short carrier diffusion length (~70 nm), and sluggish electron transfer kinetics are the intrinsic drawbacks of the pristine ms-BiVO_4_, which result in unsatisfactory photocurrent densities. Moreover, Wang et al. [23] stressed that the reduction in the capability of electrons on the CB of pure ms-BiVO_4_ (+0.04 eV vs. NHE) was weak, even though the holes on the VB of BiVO_4_ possessed strong oxidation capability, which resulted in its inability to reduce the oxygen molecule (O_2_) to a superoxide radical (O_2_^−^, −0.33 eV vs. NHE) by trapping the electrons on the CB, as well as weak surface adsorption properties [24], which stemmed from the more negative O_2_/O_2_^−^ potential than the CB of ms-BiVO_4_, and incurring a disappointing PEC conversion efficiency. In the single ms-BiVO_4_ phototrigger system, the tradeoff between an adequate redox potential and the generation of plenty of energetic photoexcited carriers according to the bandgap limits improves the PEC performances. The redox reaction on a photoelectrode is an integral whole, and it only takes place when there is an excess of photogenerated e^−^ and h^+^ on the surface. Too many free movable phototriggered carriers accumulated on the surface of the semiconductor causes deficiency in the powerful in-built electric field for efficacious delivery, which is inevitably unfavorable for PEC reactions, and the carriers are more inclined to recombine in the interiors of photocatalysts. The construction of ms-BiVO_4_/tz-BiVO_4_ (m/t-BiVO_4_) hetero-nanostructures is an alternative strategy to ameliorate the charge kinetics of ms-BiVO_4_ alone, and in particular, to facilitate the charge separation, as researchers have recently demonstrated [25]. However, the m/t-BiVO_4_ heterogeneous PEC materials are not only under harsh synthesis conditions, but they are also incapable of presenting an improvement in the energy-band-matching level to speed up the photoinduced charge segregation compared with ms-BiVO_4_/TiO_2_ and tz-BiVO_4_/TiO_2_ (m&t-BiVO_4_/TiO_2_) nano-heterostructures. Energy-band-matching m&t-BiVO_4_/TiO_2_-NTAs nano-heterojunctions with rich intrinsic oxygen vacancy (V_o_) defects exhibit an impressive catalytic activity owing to the synergistic effect between the heterojunction interface effect and vacancy effect [26]. Furthermore, researchers have extensively unveiled the intrinsic correlation between the boosted PEC-related performances and the expedited CT dynamic process associated with the V_o_ in m&t-BiVO_4_/TiO_2_-NTAs nanocomplexes. The details of the contributions of the V_o_ defects are summarized as follows [23,27,28,29]: (I) V_o_ defects can function as electron donors that increase the majority carrier density and photovoltage; (II) V_o_ could provide shallow trapping sites to promote electron–hole (e^−^–h^+^) pair segregation and restrain the recombination of the charge carrier; (III) the electronic structure can be overlapped and delocalized by V_o_ defects, which leads to an enlarged light-absorption edge; (IV) the abundant surface V_o_ defects, which are positively charged, can serve as PEC reaction centers to adsorb adequate photodegradable active group species, including O_2_^−^ and hydroxyl radicals (OH); (V) V_o_ sites contribute to the upward shift (more negative) in the Fermi level (E_F_) and the CB of BiVO_4_, and they can act as active sites to elevate the charge injection efficiency, benefitting from the favorable band-energy offset between the m&t-BiVO_4_/TiO_2_-NTAs nano-heterojunctions, which is instigated to enhance the frustrating reduction kinetics. 

Contrasted with other preparation methods, hydrothermal synthesis is a preferable approach for the formation of nano-heteroarchitectures between the m&t-BiVO_4_ and TiO_2_ NTAs. Its suitability for large-scale industrial production is due to the simple process, environmental friendliness, and low costn [30]. The pH value of the precursor solution has a substantial impact on the molar ratios of the crystalline phase and the concentration of the surface defect states for ms-BiVO_4_ and tz-BiVO_4_ [25,31], which could also have a striking impact on the energy band position and the efficiency of the interfacial CT in the heterojunction. According to the relevant research, the timescale for the interfacial CT and recombination ultrafast kinetic process of m&t-BiVO_4_/TiO_2_-NTAs nanohybrids is of a nanosecond (ns) magnitude [32], involving the rate-determining step of the surface redox reactions and making it exceedingly challenging to comprehend. The timescale is much greater than the timescale of the photoexcited e^−^–h^+^ pair transition from VB to CB (viz., the femtosecond (fs) level) [33]. Simultaneously, it is possible to integrate the probing technique of an ultrafast fs laser triggered with time-resolved photoluminescence (PL) spectroscopy into an instrument that is capable of measuring transient PL on nanosecond timescales, providing the verifiable quantitative and qualitative information on the charge carrier dynamics for the intermediate states in nano-heterostructures associated with the V_o_ intrinsic defects, including the transient PL intensity of the time-dependent photoexcited carrier lifetime, and the rate constant of the CT [34,35], which plays a decisive role in improving the PEC-related performances. Logically, by utilizing the positive synergetic effects between m&t-BiVO_4_ and TiO_2_ NTAs, we can obtain an improved PEC efficiency and highly sensitive PEC aptasensor [36,37]. However, to the best of our knowledge, the qualitative interfacial CT mechanism and quantitative charge injection dynamic process associated with V_o_ defects by taking advantage of transient-PL kinetics probing have not been comprehensively explored. Moreover, the inherent physical connection between the pH values of the precursor solution, different crystalline-phase molar ratios, and number of V_o_ defects are rarely referred to. 

First, we fabricated tidy and smooth TiO_2_ NTAs with highly ordered alignment top surfaces through the anodization approach, facilitating easy access for the uniform deposition of m&t-BiVO_4_ NPs via a convenient hydrothermal method, which we used to construct m&t-BiVO_4_/TiO_2_ NTA type-II heterostructure nanohybrids with different crystalline-phase molar ratios and the expected concentration of V_o_ defects. Moreover, the photoinduced carriers are dramatically separated and transferred at the interface between the m&t-BiVO_4_ and TiO_2_ NTAs, benefitting from the synergistic effects between the elevated band offset in staggered heterostructures and the increased exposed reaction active sites induced by the V_o_ defects, which are responsible for the boosted PEC-related performances. Additionally, we can use the unique combination of nanosecond-time-resolved transient PL (NTRT-PL) and time-resolved PL (TRPL) spectroscopy to independently track the charge carrier dynamics between the donor and acceptor energy levels, qualitative and quantitative extrapolating the CT process at the interfaces between the m&t-BiVO_4_ and TiO_2_ NTAs, which allows for the acquisition of the CT rate coefficient and charge carrier lifetime. Moreover, the established correlations between the PEC degradation performance, biosensing and gas-sensing sensitivity, and the crystalline structural feature of the m&t-BiVO_4_/TiO_2_ NTA nano-heterostructures provide insight into the expected interfacial energy band alignment related to the V_o_ defect concentration, which elevates the ultrafast injection of the free carriers from BiVO_4_ into TiO_2_ NTAs. Furthermore, the research highlights the importance and novelty of probing interfacial CT kinetic processes modulated by V_o_ surface defects for understanding the mechanisms of PEC conversion in m&t-BiVO_4_/TiO_2_-NTAs nanocomplexes. We hope that the investigation can provide practical experiences and in-depth comprehension for the design of PEC-related devices with substantially superior performances.

## 2. Experimental Section

### 2.1. Preparation of TiO_2_-NTAs Substrates via Anodization

We bought all the reagents and solvents from commercial sources, and we used them without any further purification. We prepared the TiO_2_ NTAs by anodizing Ti foils in an electrolyte containing NH_4_F (0.45 wt.%) and ethanediol (98 vol.%). This process resulted in the formation of TiO_2_ NTAs on the surface of the Ti foils, which researchers described in a previous report [35].

### 2.2. Fabrication of m&t-BiVO_4_/TiO_2_-NTAs Heterostructure Nanohybrids

The BiVO_4_ NPs were deposited onto the surface of the fabricated TiO_2_ NTAs via a facile low-temperature hydrothermal method. In brief, we sequentially dissolved a 2 mL concentration of 0.1 M of Bi(NO_3_)_3_ 5H_2_O and a 2 mL concentration of 0.1 M of NH_4_VO_3_ in 19 mL of ethylene glycol, and we then added a 1 mL concentration of 2.0 M of HNO_3_ to form a precursor solution. Adding HNO_3_ aqueous solution can help dissolve other reagents and render the resulting solution more acidic. We adjusted the mixture to a certain pH value (2, 5, or 8) by slowly adding ammonia with a magnetic stirrer to obtain different crystalline-phase molar ratios of m&t-BiVO_4_. We transferred the orange transparent precursor into a Teflon-lined stainless-steel autoclave (50 mL) after vigorous stirring for 30 min, and we maintained it at 100 °C with different hydrothermal deposition times (5 h, 10 h, and 20 h). We vertically placed the preprepared highly ordered TiO_2_ NTAs in the autoclave in advance though a home-built Teflon sample holder.

As a reference for characterizing the optical and PEC performances, we prepared the pristine BiVO_4_ films by the hydrothermal route. Briefly, we methodically dissolved 0.2 mmol of Bi(NO_3_)_3_ 5H_2_O, 0.2 mmol of NH_4_VO_3_, and 1 mL of 2.0 M HNO_3_ in 19 mL of ethylene glycol. We adjusted the pH value of the sample suspension to 5 with ammonia, and then vertically placed the prearranged clean FTO conductive glass substrate into the mixed solution and maintained it at 100 °C for 10 h.

**Scheme 1:**
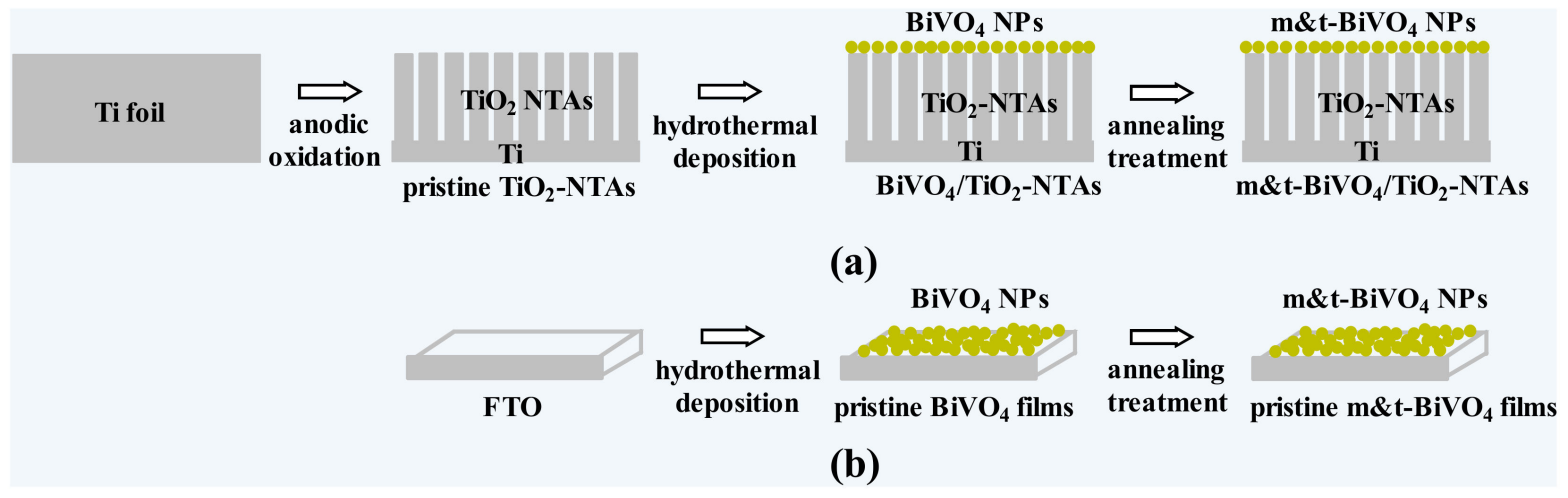
Preparation process of m&t-BiVO_4_/TiO_2_-NTAs nanocomplex (**a**), and pristine BiVO_4_ films (**b**).

After we rinsed the as-prepared specimens with deionized water, we dried them with a nitrogen gas flow. For facilitating the generation of intrinsic defects and the expected crystalline phases, we annealed the as-obtained specimens in a furnace in dry air. The annealing temperature was 450 °C, and the time was 30 min. The heating rate was 10 °C/min, and the cooling rate was 10 °C/min. The choice of the annealing temperature was deliberate to avoid the transformation of the crystal structure of t-BiVO_4_ into m-BiVO_4_ during the annealing process (annealing temperature > 500 °C) [38]. We present the details on the process of incorporating modified BiVO_4_ NPs onto FTO and TiO_2_ NTA surfaces with the same area size (1 cm × 1.5 cm) in Scheme 1.

### 2.3. Characterization

We characterized the microstructures of the as-prepared nano-heterostructures by scanning electron microscopy (SEM) (Hitachi S4200) and transmission electron microscopy (TEM) (JEOL JEM-2100). We performed the UV–visible diffuse reflectance spectrometry (UV–vis DRS) measurements using a UV–vis spectrophotometer (UV-1800, Shimadzu). We characterized the phase purity of the prepared specimens by an X-ray diffractometer (XRD) (Shimadzu XRD-600). We used micro-Raman spectroscopy to investigate the crystal structure and chemical bonding states of the nanocomposites, equipped with a confocal microscope with an Ar^+^ laser operating at 532 nm (Horiba JY-HR800). We investigated the oxidation states of the as-formed samples by X-ray photoelectron spectroscopy (XPS) (ESCALAB 250). We determined the instrument’s resolution to be 1.0 eV from the Ag 3d_5/2_ peak’s full width at half maximum. We calibrated the XPS energy scale by aligning the Ag 3d_5/2_ line on clean silver with the E_F_, which we set at 368.3 eV. The XPS spectra energy axis shifted due to the specimen charging that takes place during X-ray irradiation. Consequently, we set the C1s binding energy line to 285.0 eV, which is the standard hydrocarbon energy used to reference the charging effect.

We used CHI660E equipment (Chenhua) to perform the PEC-related performance tests. We performed the transient photocurrent density curve (transient I–t curve) measurements via frontside illumination under AM 1.5G (SS150A, ZOLIX) at a constant applied potential of 0 V in a 0.1 M Na_2_SO_4_ solution. The 0.2 M Na_2_SO_4_ solution acted as the electrolyte for the electrochemical impedance spectra (EIS) analysis. We constructed Mott–Schottky plots at 1 kHz to examine the relationship between the voltage and capacitance in the 0.5 M Na_2_SO_4_ solution. We adopted the following equation for the NHE potentials: E_NHE_ = E_Ag/AgCl_ + 0.1976 V, where E_Ag/AgCl_ is the Ag/AgCl electrode.

**Figure 1:**
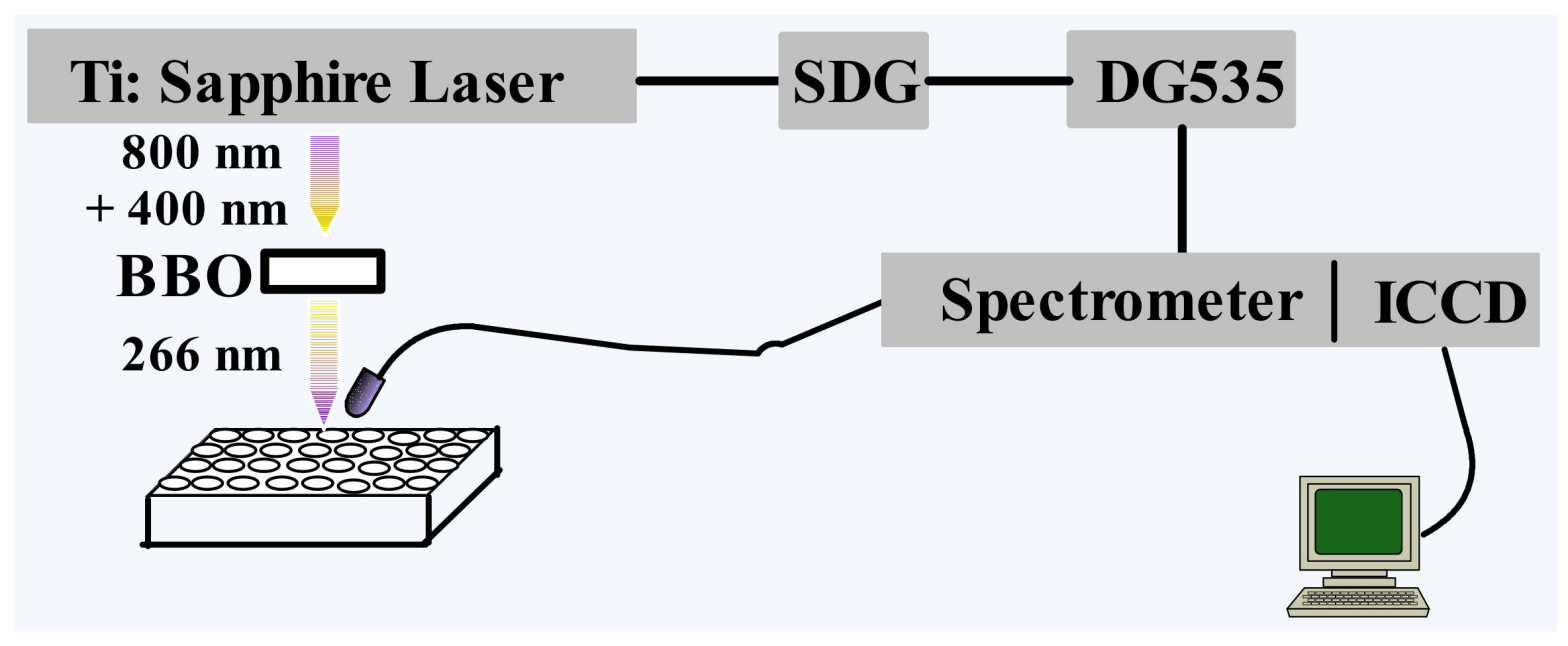
Schematic of experiment setup for NTRT-PL measurements.

We excited the NTRT-PL using a Ti:sapphire femtosecond (fs) laser system (Spectra-Physics). We present the schematic diagram of the experimental setup in Figure 1. We collected the data for the TRPL using a custom-built single-photon-counting system, and the excitation source wavelength was 375 nm. We dispersed the signals of the PL emission for Vo defects (λ_em_ = 2.9 eV) by means of a grating spectrometer, and we detected them by a high-speed photomultiplier tube conjunction with a single-photon-counting card.

### 2.4. Performance of MO Photodegradation, PEC Biosensing, and Gas-Sensing Measurements

We assessed the photocatalytic activities of the as-prepared nano-heterojunctions using the photodegradation of MO under UV–visible light irradiation. We used the UV–vis spectrophotometer to assess the concentration of the MO solution (10 mg/L) every 20 min. We monitored the intensity change in the characteristic absorption peak at 465 nm in order to determine the concentration of the MO solution. We used a concentration of 2 mM methanol solution, isopropanol (IPA) as holes (h^+^), and hydroxyl radicals (OH) scavengers to detect the reactive species [39].

We measured the PEC biosensing performance by the electrochemical workstation (CHI660E). Simultaneously, the supporting electrolyte was a 0.1 M solution of phosphate-buffered saline (PBS) (pH = 7.0) We determined the detection limit to be 0.5 V by observing the lowest value that could be distinguished from the background signal. We investigated the gas-sensing properties of the as-synthesized nanohybrids by a home-built test chamber (1 L). We used a nanoamperemeter (GT8230) to document the measuring current as a function of the exposure time by applying a sweep voltage (5V) under 100 ppm of NH_3_.

## 3. Results and Discussions

**Figure 2:**
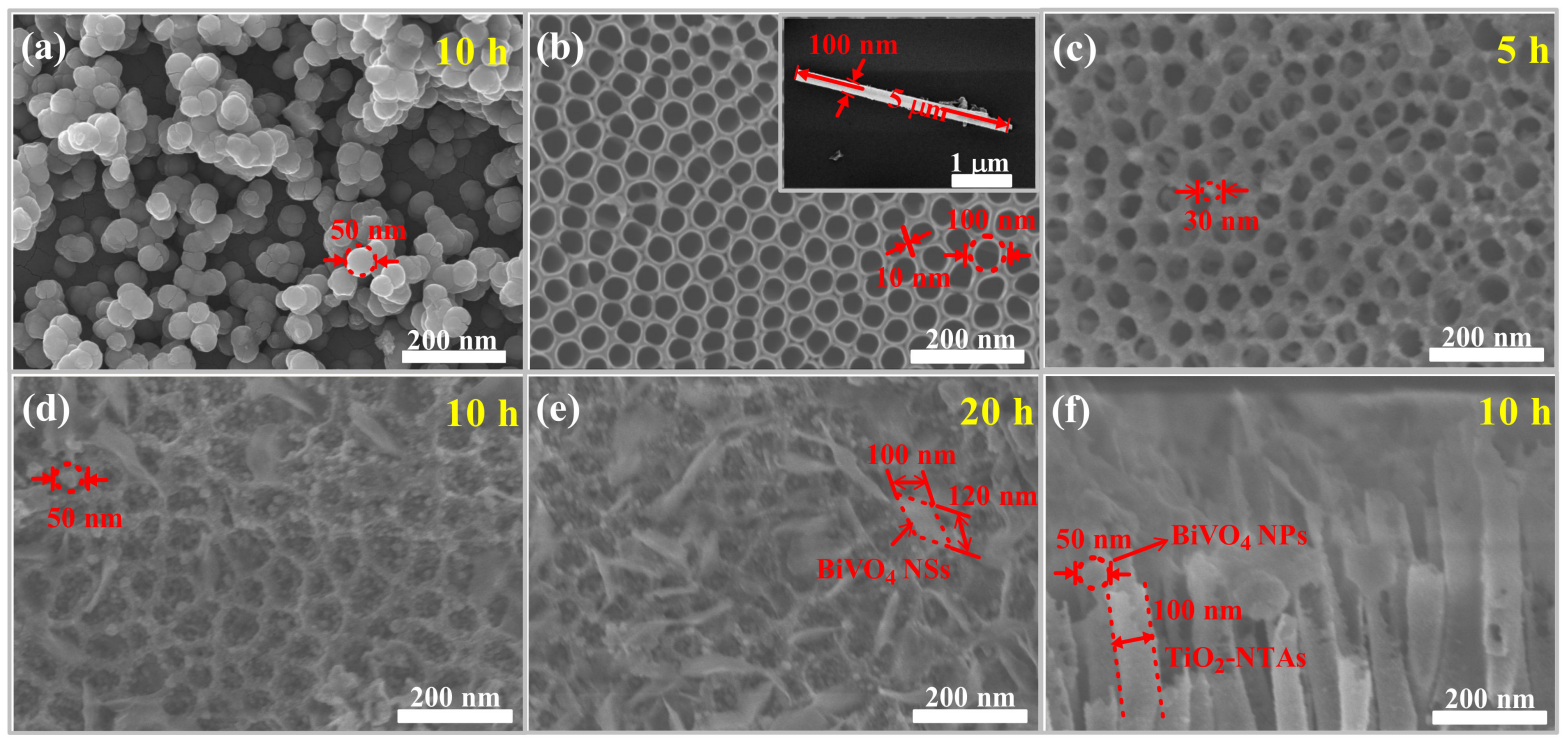
SEM pictures of (**a**,**b**) top-view for as-formed pristine BiVO_4_ films and pure TiO_2_-NTAs, and low-resolution TEM picture for individual nanotube of TiO_2_ inset in figure (**b**). (**c**–**e**) Top-view SEM images of BiVO_4_/TiO_2_-NTAs with BiVO_4_ hydrothermal-deposition times of 5 h, 10 h, and 20 h, respectively. (**f**) SEM picture with cross-section for as-obtained BiVO_4_/TiO_2_-NTAs nanoheterostructures with BiVO_4_ nanoparticles (NPs) deposited time 10 h.

We used SEM and TEM to characterize the surface morphology and cross-sectional arrangement of the pure TiO_2_ NTAs, referenced pristine BiVO_4_ films, and binary BiVO_4_/TiO_2_-NTAs nanohybrids with different BiVO_4_ NP hydrothermal synthesis times (5 h, 10 h, and 20 h), as displayed in Figure 2. We prepared the BiVO_4_ NPs with uniform size and regular spherical-shaped distribution on the FTO conductive surface by the hydrothermal method, and the average particle size of the BiVO_4_ NPs was about 50 nm. Moreover, we found spherical-form BiVO_4_ NPs with agglomeration in some places, which enhance the specific surface area, which increases the active surface area for the redox reactions [40]. Simultaneously, we manufactured the TiO_2_ NTAs with smooth and uniform top surfaces on the Ti substrate, and the average pore diameter and wall thickness are about 100 nm and 10 nm, respectively, as depicted in Figure 2b. The inset in Figure 2b is the TEM image of a single nanotube, illustrating that the outer diameter and length are separately ca. 100 nm and 5 μm, respectively, which accord well with the top-view SEM observation. We present the typical SEM images for the vertical-view morphologies of the BiVO_4_/TiO_2_-NTAs nanohybrids for increasing the BiVO_4_ NP hydrodeposition times from 5 h to 20 h with a precursor solution with pH values successively equal to 2, 5, and 8 after annealing at 450 °C in Figure 2c–e. We observed distinctly different nanotopographies (from dispersed or aggregated spherical-shaped NPs to clustered nanosheets) after the decoration of BiVO_4_ onto the surfaces of the TiO_2_ NTAs. We present the SEM image for the BiVO_4_/TiO_2_-NTAs nanocomposites with a BiVO_4_ NP hydrothermal preparation time of 5 h (BiVO_4_/TiO_2_-NTAs-5) in Figure 2c, which we could use to precisely examine the discrete distribution of BiVO_4_ NPs with an average size of nearly 30 nm, which embrace upon opening and infill the interstices of TiO_2_ NTAs. Furthermore, we present the overhead-view SEM image of BiVO_4_/TiO_2_-NTA-10 in Figure 2d. The BiVO_4_ NPs with a mean size of ca. 50 nm are evenly distributed on the top surface of the nanotube, as well as in the intertube space, and they also fill the interior of the nanotubes, connecting them together. The skeleton of the TiO_2_ NTAs remains unchanged. BiVO_4_ nanosheets (NSs) formed in some areas on the surfaces of the TiO_2_ NTAs. The top-view SEM image of the BiVO_4_/TiO_2_-NTAs nanocomposites with a BiVO_4_ hydrothermal reaction time of 20 h, labeled as BiVO_4_/TiO_2_-NTAs-20, are exhibited in Figure 2e. The result further verified that the synthetic reaction time and pH value for the preparation of BiVO_4_ are essential factors, as they have a tendency to aggregate together by forming NS clusters, randomly distributed on the top surfaces of the nanotubes, with a length and width of approximately 120 nm and 100 nm, respectively, which are in good agreement with the published article in terms of the occurrence of the introduction of foreign species at the surface, which usually block the nanotube openings [41]. 

To definitively illustrate the formation of BiVO_4_/TiO_2_-NTAs nano-heterostructures, we performed a cross-sectional SEM characterization on a representative specimen of BiVO_4_/TiO_2_-NTAs-10, as presented in Figure 2f. The incorporation of BiVO_4_ NPs led to an increase in the surface roughness of the BiVO_4_/TiO_2_-NTAs-10 nanohybrids, which indicated that the particle size of the BiVO_4_ and the outer diameter of the individual TiO_2_ nanotube were about 50 nm and 100 nm, respectively, which coincide with the results of Figure 2b,d. Most notably, the deposited BiVO_4_ NPs screened the entrances of the TiO_2_ NTAs, successfully preparing the BiVO_4_/TiO_2_-NTAs nano-heterostructures.

**Figure 3:**
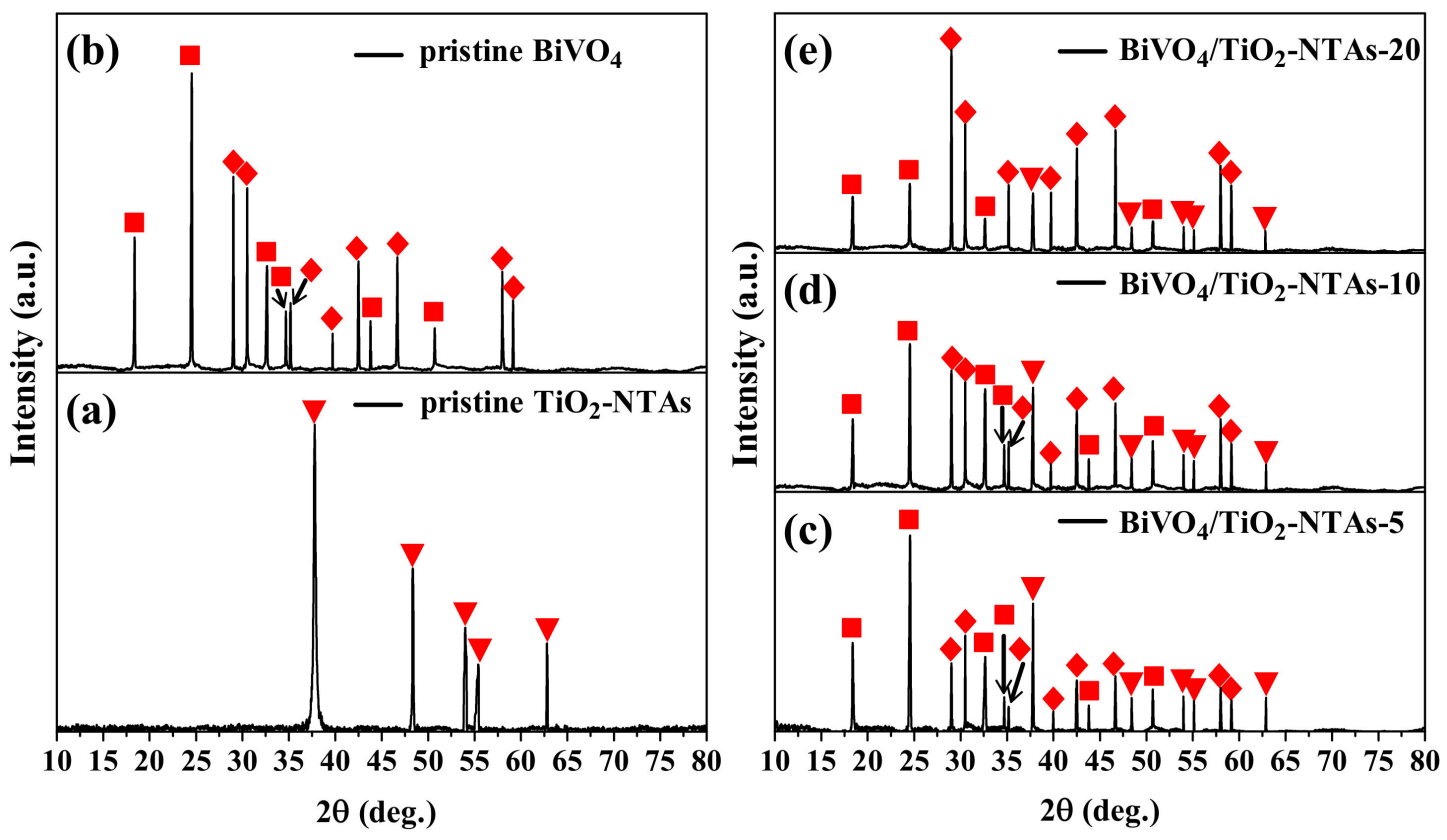
XRD of pristine TiO_2_-NTAs (**a**,**b**) pure BiVO_4_ films, and (**c**–**e**) BiVO_4_/TiO_2_-NTAs nanohybrids with different hydrothermal deposited amounts, respectively.

We examined the crystalline structures and phase compositions of the as-prepared films in depth by XRD. We present the XRD patterns of the pristine TiO_2_ NTAs, pure BiVO_4_ films, and BiVO_4_/TiO_2_-NTAs binary nano-heterojunctions in Figure 3. The BiVO4/TiO_2_-NTAs dual nano-heterostructures with various thermal depositing times (5 h, 10 h, and 20 h) correlate with the different pH values (2, 5, and 8, respectively) of the precursor solutions. Evidently, all the specimens had narrowed and sharpened peaks under the hydrothermal crystallization conditions, proving that the as-fabricated products were crystalline, as expected, which is consistent with a previous report [42]. As shown in Figure 3a, there are five diffraction peaks of the untampered TiO_2_ NTAs (labeled with a ‘▼’ mark), with diffraction 2θ angles located at 37.88°, 48.12°, 53.97°, 55.10°, and 62.74°, which are indexed to the (004), (200), (105), (211), and (204) diffraction planes, respectively. These results suggest that the sample is anatase TiO_2_ (Card No. 21-1272). The anatase TiO_2_, in particular, has a better PEC performance than the other crystal phases, resulting from the smaller effective mass and longer carrier lifetime, which result in a faster migration rate and higher generation of active species for PEC reactions [43]. We present the XRD patterns of the as-obtained BiVO_4_ films with pH values equal to 5 of the precursor solution annealing at 450 °C in Figure 3b, in which it can be seen that the locations of the diffraction peaks coincide with the tz-BiVO_4_ and ms-BiVO_4_ phases, according to the standard JCPDS files, which proves the successful fabrication of the m/t-BiVO_4_ isotype crystal-phase heterojunctions. Specifically, the prominent peaks sited at 18.3°, 24.4°, 32.7°, 34.7°, 43.8°, and 50.7° typically correspond to the (101), (200), (112), (220), (103), and (213) crystal planes (labeled with a ‘■’ mark) of the tz-BiVO_4_ (Card No. 14-0133), respectively. Simultaneously, the ms-BiVO_4_ sample displays the characteristic diffraction profiles at 28.8°, 30.5°, 35.2°, 39.7°, 42.5°, 46.7°, 58.0°, and 59.2° (denoted by “♦” mark), corresponding to the (121), (040), (002), (211), (051), (240), (170), and (123) crystal planes, which is in good accordance with Card No. 14-0688. We probed the crystallinities of the hydrothermally precipitated BiVO_4_ NPs decorated on the TiO_2_ NTAs with various deposition amounts (from 5 h to 20 h) in different pH values (from 2 to 8) of the precursor solution by XRD, as presented in Figure 3c–e. 

Compared with the diffraction peak positions of Figure 3a,b, the XRD patterns for Figure 3c–e vividly imply the lack of impurity peaks, except in the crystal phases of the TiO_2_ NTAs, ms-BiVO_4_, and tz-BiVO_4_ in the BiVO_4_/TiO_2_-NTAs samples, demonstrating the high purity of the hydrothermal treatment and the prospective achievement of binary m&t-BiVO_4_/TiO_2_-NTAs heterostructure nanocomposites (i.e., ms-BiVO_4_/TiO_2_-NTAs and tz-BiVO_4_/TiO_2_-NTAs nano-heterojunctions). Simultaneously, the XRD patterns of the m&t-BiVO_4_/TiO_2_-NTA nanohybrids show all the diffraction peaks of the anatase phase TiO_2_, which means that the original structure of the TiO_2_-NTAs was maintained during the BiVO_4_ coating process. The intensities of the diffraction profiles for the TiO_2_-NTAs in the patterns of pristine TiO_2_-NTAs and m&t-BiVO_4_/TiO_2_-NTAs nano-heterojunctions decreased with the increasing hydrothermal BiVO_4_ deposition times from 0 h to 20 h, mainly owing to the blocking effect of the heterogeneous interface between the m&t-BiVO_4_ and TiO_2_-NTAs [44], which gradually weakens the XRD signal strength of the anatase TiO_2_-NTAs substrates with the increasing deposition amount of BiVO_4_. For the as-prepared samples of the BiVO_4_/TiO_2_-NTAs activated at 450 °C, we probed the mixed heterogeneous phases of the m&t-BiVO_4_/TiO_2_-NTAs, and the research data are in agreement with the achievement reported by Parida et al., who demonstrated the coexistence of m&t-BiVO_4_, as the samples were annealed from 300 °C to 600 °C [45]. More notably, when the pH values for the precursor solution increased successively from 2 to 8, the diffraction peak intensities of (121) and (040) progressively increased for the ms-BiVO_4_/TiO_2_-NTAs nano-heterostructures, while those of (101) and (200) gradually declined for the tz-BiVO_4_/TiO_2_-NTAs nano-heterojunctions, as plotted in Figure 3c–e. To quantitatively evaluate the proportions of tz-BiVO_4_ (η_tz-B/T_) and ms-BiVO_4_ (η_ms-B/T_) in the specimens of pristine BiVO_4_, BiVO_4_/TiO_2_-NTAs-5, BiVO_4_/TiO_2_-NTAs-10, and BiVO_4_/TiO_2_-NTAs-20, we made the estimates using Equations (1) and (2) [46,47]. I_tz-B/T_ and I_ms-B/T_ refer to the relative intensities of the diffraction profiles for the tetragonal phase (i.e., (101) and (200)) and monoclinic phase (i.e., (121) and (040)), respectively. We present the details on the percentage compositions of the η_tz-B/T_ and η_ms-B/T_ in the single and dual nano-semiconductors in Table 1.

η_tz-B/T_ (%) = (I_tz-B/T_ × 100%)/(I_tz-B/T_ + I_ms-B/T_) (1)

η_ms-B/T_ (%) = (I_ms-B/T_ × 100%)/(I_tz-B/T_ + I_ms-B/T_) (2)

Table 1 indicates that the fractions of η_ms-B/T_ (or η_tz-B/T_) in the m&t-BiVO_4_/TiO_2_-NTAs binary nano-heterojunction samples increased (or decreased) with the increasing pH values of the precursor solution, and vice versa, manifesting the efficacious construction of the different ratios of m&t-BiVO_4_/TiO_2_-NTAs nanohybrids, which was achieved by controlling the pH values, which was entirely in agreement with the results reported by Huang et al. [25]. We believe that the increased pH values for the precursor inhibit the crystal growth of the tz-BiVO_4_ due to the enlargement of ms-BiVO_4_. The proportion composition of ms-BiVO_4_ and tz-BiVO_4_ in the individual BiVO_4_ films is approximately equal to that in the BiVO_4_/TiO_2_-NTAs-10 specimen, which verifies the formation of ms/tz-BiVO_4_ heterojunctions in BiVO_4_ films alone, and further validates the crucial role of the pH value in mediating the heterostructure ratio for m&t-BiVO_4_/TiO_2_-NTAs nanocomplexes under the determined annealing temperature.

**Figure 4:**
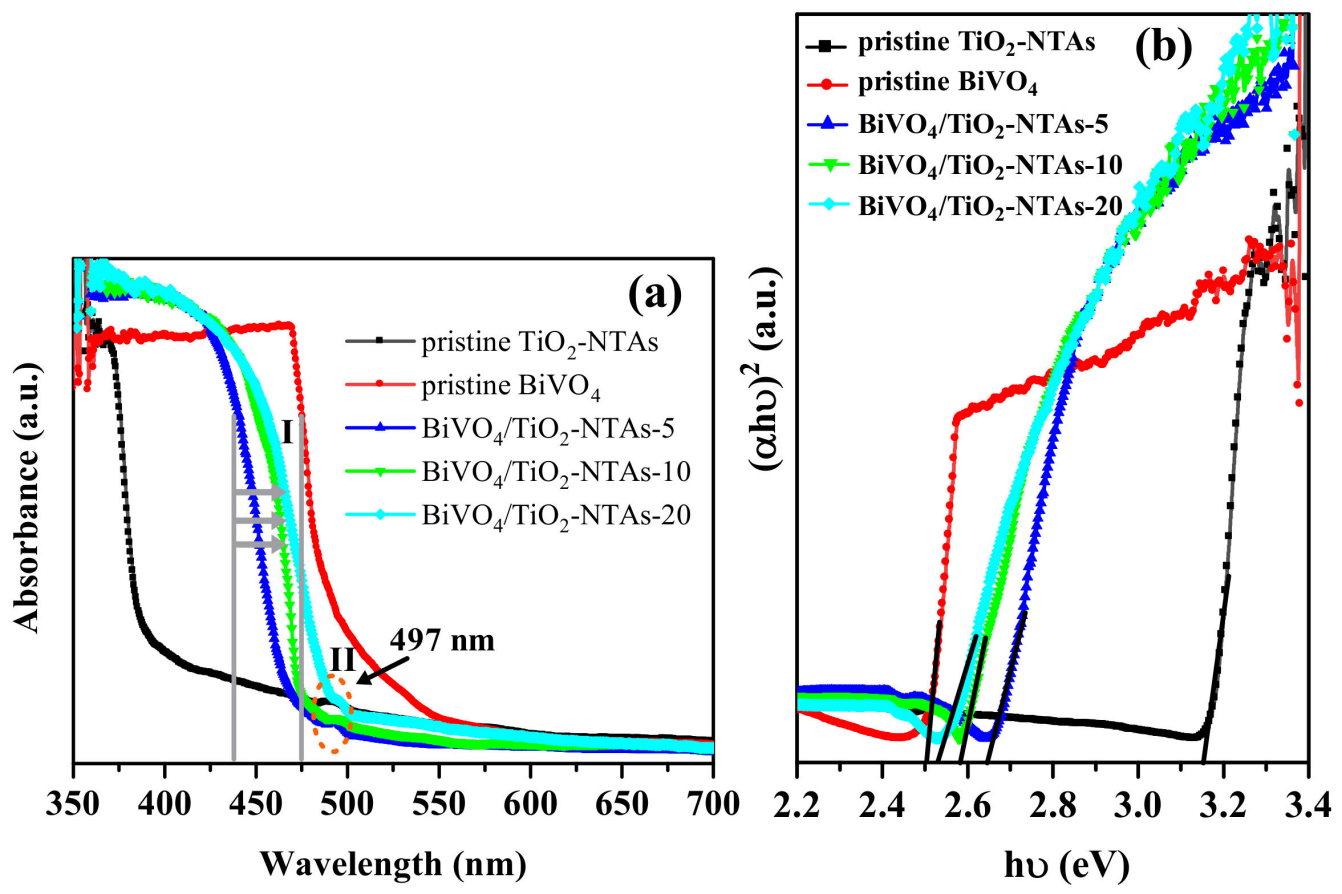
UV-vis DRS spectra (**a**,**b**) showed band-gap energy of pure TiO_2_-NTAs, pristine BiVO_4_ films, and BiVO_4_/TiO_2_-NTAs nanoheterostructure compositions with different BiVO_4_ hydrothermal-deposited amounts, respectively.

As represented in Figure 4, we performed UV–vis DRS measurements and derived Tauc plots to evaluate the optical absorption intrinsic properties and bandgap values of the as-obtained specimens for unitary and binary semiconductors, respectively, which are indispensable characterization instruments for constructing superior photoresponse nanohybrids. We present the UV–vis DRS detection by a wavelength-dependent absorbance between 350 nm and 700 nm for the pristine TiO_2_-NTAs, pure BiVO_4_ films, and m&t-BiVO_4_/TiO_2_-NTAs binary nano-heterojunctions with varying BiVO_4_ NP hydrothermal preparation times (5 h, 10 h, and 20 h) in Figure 4a. The absorption edges of the TiO_2_-NTAs alone are nearly identical at 393 nm, which is due to the transition of the near band edge (NBE) [48]. The UV–vis DRS spectrum pattern for the pristine BiVO_4_ films is perceptibly redshifted compared with that of the single TiO_2_-NTAs in Figure 4a, exhibiting the characteristic spectrum related to 496 nm, which is between the intrinsic absorption band edges of ms-BiVO_4_ (517 nm) and tz-BiVO_4_ (428 nm). Furthermore, as illustrated in the traces of UV–vis DRS for m&t-BiVO_4_/TiO_2_-NTAs nano-heterostructures, the absorption edges of the dual nanohybrids are substantially transparent to longer wavelength regions in comparison with the pure TiO_2_ NTAs, which indicates that the incorporation of BiVO_4_ elevates the absorption ability and facilitates the transport of photogenerated electrons, which is the result of the synergistic effect of the heterostructure between m&t-BiVO_4_ and TiO_2_ NTAs. Evidently, with the increase in the hydrothermal times from m&t-BiVO_4_/TiO_2_-NTAs-5 to m&t-BiVO_4_/TiO_2_-NTAs-20, there is a gradual shift of the absorbance boundary to a larger wavelength, which indicates that the bandgap is reducing, and the material is becoming more sensitive to visible light. Simultaneously, all the absorption patterns of the m&t-BiVO_4_/TiO_2_ NTAs with various amount of BiVO_4_, and especially for m&t-BiVO_4_/TiO_2_-NTAs-10, present conspicuous forward saddle-backing shapes in the visible region (i.e., labeled as area I), which are probably traceable to the increased average atomic distance induced by the V_o_ defects in BiVO_4_ [27]. Additionally, the absorption peaks located at 497 nm, labeled as area II, are ascribed to the absorption of the V_o_ defects in TiO_2_ NTAs [49]. The corresponding E_g_ values of the as-prepared specimens can subsequently be determined using Tauc plots, as exhibited in Figure 4b. The (αhυ)^1/n^ against the photon energy (hυ) curves are plotted basing on the following classic Tauc equation [50]:

(αhυ)^1/n^ = A(hυ − E_g_) (3)

The absorption coefficient, Planck’s constant, incident light frequency, proportionality constant, bandgap energy, and characteristic integer are denoted by α, h, υ, A, E_g_, and n, respectively. The n value depends on the nature of the optical transition, and because of the characteristic direct transitions of BiVO_4_ and TiO_2_, the value of n is 1/2 [51]. Logically, by extrapolating the linear portion of (αhν)^2^ to zero, we can estimate the E_g_ for the as-formed samples. The predicted E_g_ values for the pristine TiO_2_ NTAs, bare BiVO_4_ films, m&t-BiVO_4_/TiO_2_-NTAs-5, m&t-BiVO_4_/TiO_2_-NTAs-10, and m&t-BiVO_4_/TiO_2_-NTAs-20 are about 3.15 eV, 2.50 eV, 2.65 eV, 2.58 eV, and 2.52 eV, respectively. In order to further evaluate the validity of the calculated E_g_ values for the m&t-BiVO_4_/TiO_2_-NTAs under different hydrothermal-synthesis conditions, we can use the alternative bandgap prediction method associated with the weighting of the m&t-BiVO_4_ content, as follows:

E_g-W_ = E_g-ms_ × η_ms-B/T_ (%) + E_g-tz_ × η_tz-B/T_ (%) (4)

where E_g-W_, E_g-ms_, E_g-tz_, η_ms-B/T_, and η_tz-B/T_ are the bandgap energies for the weighted contents. The E_g_ for ms-BiVO_4_ is 2.4 eV, while the E_g_ for tz-BiVO_4_ is 2.9 eV, which are the percentages of the monoclinic and tetragonal phases of BiVO_4_ in m&t-BiVO_4_/TiO_2_-NTAs nano-heterojunctions, respectively. For convenience, we list the detailed comparison results in Table 2. By stripping out the impact of the TiO_2_-NTAs substrates for the energy-band structures of the m&t-BiVO_4_/TiO_2_-NTAs with different preparation conditions, the obtained E_g_ from the Tauc formula and valuated E_g-W_ are essentially consistent, as expected, which reinforces the assumption that the presence of BiVO_4_ associated with V_o_ provides a synergistic enhancement in the visible-light absorption, which, in turn, promotes the energy coupling between photons and excitons. The difference in the E_g_ values between pristine BiVO_4_ and m&t-BiVO_4_/TiO_2_-NTAs-10 under the same hydrothermal manufabricated environment probably derives from the discrepancy in the thickness of the photoactive layer between them [52].

**Figure 5:**
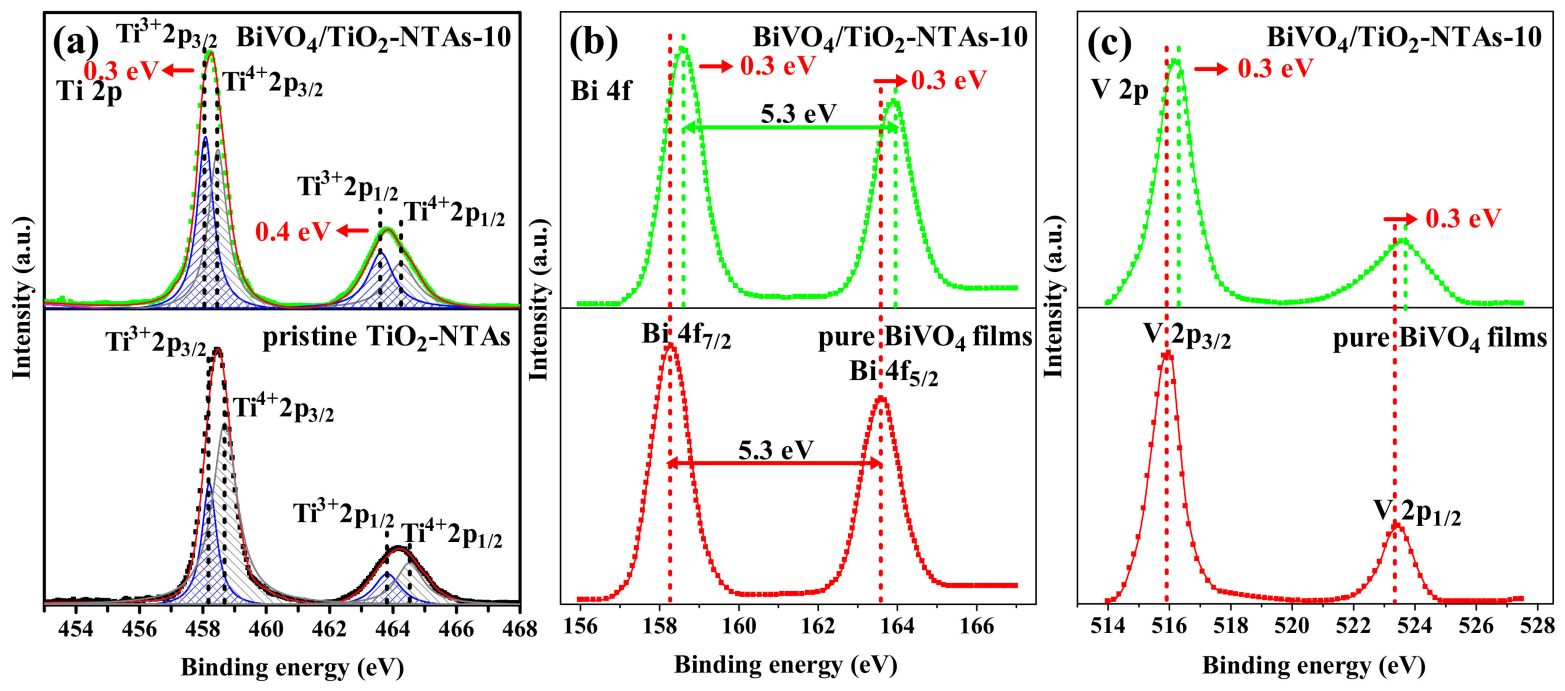
High-resolution X-ray photoelectron spectroscopy characterizations of (**a**) Ti 2p, (**b**) Bi 4f, and (**c**) V 2p core level for pure TiO_2_-NTAs, pristine BiVO_4_ films, and m&t-BiVO_4_/TiO_2_-NTA-10, respectively.

In order to further intuitively unveil the synergistic effect of BiVO_4_ and TiO_2_ NTAs on the surface defects, which profoundly influence the CT process and the performance of PECs, we analyzed the chemical components and bonding configurations of the as-prepared nano-heterojunctions using XPS, as exhibited in Figure 5.

Specifically, in Figure 5a, we plot the high-resolution XPS spectra (HR-XPS) of the Ti 2p core level for the pristine TiO_2_-NTAs and BiVO_4_/TiO_2_-NTAs-10 dual nanocomposites. We fit the experimental data points (black and green dots) with a curve (red solid line) using a mixed Gaussian–Lorentzian function. We chose this function because it provided the optimized fit to the data points, as determined by the nonlinear least-squares fitting algorithm, including Ti^3+^2p_3/2_, Ti^4+^2p_3/2_, Ti^3+^2p_1/2_, and Ti^4+^2p_1/2_, which originated from the core levels of Ti^3+^ and Ti^4+^. The two intense peaks in the pure TiO_2_ NTAs at the binding energies (BEs) sited at 458.5 eV and 464.2 eV represent Ti 2p_3/2_ and Ti 2p_1/2_ [53], respectively. Moreover, the BE of the Ti 2p core level for the referenced BiVO_4_/TiO_2_-NTAs-10 centered at 458.2 eV and 463.8 eV are assigned to Ti 2p_3/2_ and Ti 2p_1/2_ [54], respectively. Additionally, the BE values of the different oxidation states of the Ti atoms are distinct. The BE peaks located at 458.7 eV, 464.5 eV, 458.5 eV, and 464.3 eV are attributed to the Ti^4+^ valence state [55], and the BE peaks positioned at 458.1 eV, 463.6 eV, 458.2 eV, and 463.9 eV are attributed to the Ti^3+^ valence state and V_o_ defects in the TiO_2_ NTAs [54,56,57]. We present the surface atomic Ti^3+^/Ti^4+^ ratios of the pristine TiO_2_-NTAs specimens and binary m&t-BiVO_4_/TiO_2_-NTAs-10 nano-heterojunctions in Table 3, which we obtained by calculating the integral fitting of the peak areas for the concentration of the spin-orbit-splitting Ti 2p_1/2_ and Ti 2p_3/2_ core levels in the Ti 2p XPS spectra, which directly correspond to the concentrations of the V_o_ defects (Ti^3+^) and Ti^4+^ [58]. We found an explicit shift of 0.3–0.4 eV to a low BE (redshift) in the peak positions of Ti 2p for m&t-BiVO_4_/TiO_2_-NTAs-10 compared with the pristine TiO_2_ NTAs, which mainly derived from the CT from the BiVO_4_ to TiO_2_ after the formation of the heterostructures [59], which increased the electron density and V_o_ defect concentration in the TiO_2_ NTAs [60].

As can be observed in Figure 5b, the HR-XPS of the Bi 4f core level in the pure BiVO_4_ films reveals two peaks at 158.3 eV and 163.6 eV, which index to the orbits of Bi 4f_7/2_ and Bi 4f_5/2_ [61], respectively. In contrast, the spin-orbit splitting of Bi 4f (158.6 eV and 163.9 eV) for BiVO_4_/TiO_2_-NTAs-10 shift to a higher BE by approximately 0.3 eV, relative to its values for pristine BiVO_4_ films. The separation between the splitting of the two spin orbits of Bi 4f is 5.3 eV for both the BiVO_4_ films alone and BiVO_4_/TiO_2_-NTAs-10, which is attributed to the expected oxidation state of Bi^3+^ in BiVO_4_ [62]. As plotted in Figure 5c, two spin-orbit-splitting peaks of V 2p are centered at 515.9 eV and 523.4 eV for the pure BiVO_4_ films, assigned to V 2p_3/2_ and V 2p_1/2_, respectively, and certifying the existence of the V^5+^ oxidation state in BiVO_4_ [63]. Compared with that of bare BiVO_4_, the V 2p spin-orbit peaks of BiVO_4_/TiO_2_-NTA-10 (i.e., V 2p_3/2_ and V 2p_1/2_) shifted to the higher BE at around 0.3 eV of 516.2 eV and 523.7 eV, respectively. The phenomenon of the BE shift to greater values for the Bi 4f and V 2p core levels for BiVO_4_/TiO_2_-NTAs-10 than pure BiVO_4_ are indicative of the electron migration from BiVO_4_ to TiO_2_ between the different components in the interface of the nanocomposites, which suggests the weakened electron screening effect because of the decrease in the electron density for the BiVO_4_ [64], which is consistent with the aforementioned XPS analysis of Ti 2p for BiVO_4_/TiO_2_-NTAs-10.

**Figure 6:**
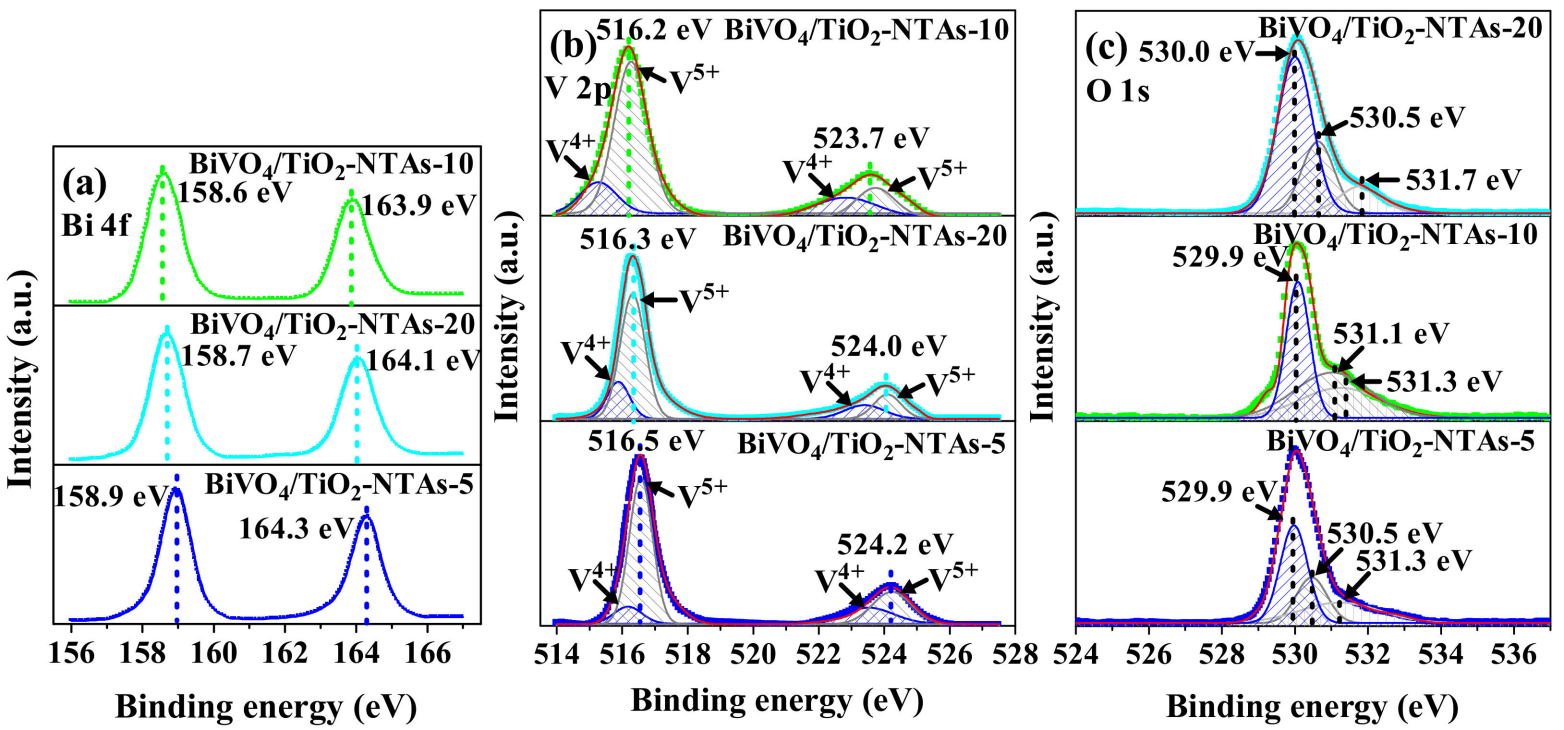
HR-XPS spectroscopy characterizations of (**a**) Bi 4f, (**b**) V 2p, and (**c**) O 1s core levels for m&t-BiVO_4_/TiO_2_-NTAs heterojunctions nanohybrids with different hydrothermal-synthesis times 5 h, 10 h, and 20 h, respectively.

We applied XPS detection to further certify the influence of the various pH values of the precursor (2, 5, 8) on the surface valence states and V_o_ defect concentration of the interface in the as-prepared nano-heterostructures of m&t-BiVO_4_/TiO_2_-NTAs with different hydrodeposition times (5 h, 10 h, and 20 h). We present the Bi 4f-core-level high-resolution XPS spectra of the m&t-BiVO_4_/TiO_2_-NTA nano-heterojunctions with precipitation times of 5 h, 10 h, and 20 h in Figure 6a. The split BE peaks of Bi 4f appear at 158.6 eV–158.9 eV and 163.9 eV–164.3 eV for Bi 4f_7/2_ and Bi 4f_5/2_, respectively, which are characteristics of the trivalent oxidation state of Bi element species [61,65,66]. Compared with the m&t-BiVO_4_/TiO_2_-NTAs-5 sample (158.9 eV and 164.3 eV for Bi 4f_7/2_ and Bi 4f_5/2_, respectively), the spin-orbit splitting of the Bi 4f_7/2_ and Bi 4f_5/2_ signals for BiVO_4_/TiO_2_-NTA-20 (158.7 eV and 164.1 eV, respectively), and BiVO_4_/TiO_2_-NTAs-10 (158.6 eV and 163.9 eV, respectively) slightly shift towards lower BE values by 0.2 eV and 0.3 eV, respectively, sufficiently attesting to the interfacial interaction that is formed in a typical m&t-BiVO_4_/TiO_2_-NTAs heterojunction sample. We present the core level XPS spectra of the V 2p for m&t-BiVO_4_/TiO_2_-NTAs nanohybrids with deposition times of 5 h, 10 h, and 20 h in Figure 6b. Two asymmetric BE peaks are centered at 516.5 eV and 524.2 eV for the V 2p of m&t-BiVO_4_/TiO_2_-NTAs-5, which are ascribed to the characteristic spin-orbit signals of V 2p_3/2_ and V 2p_1/2_ [67], respectively, while the broad V 2p XPS spectra of m&t-BiVO_4_/TiO_2_-NTAs-10 and m&t-BiVO_4_/TiO_2_-NTAs-20 exhibited characteristic splitting BE peaks at 516.2–516.3 eV and 523.7–524.0 eV, respectively, which can be assigned to the V 2p_3/2_ and V 2p_1/2_ spin-orbit signals [68,69], respectively. Coincidentally, besides the core level XPS spectra of Bi 4f, we distinctly observed a slight shift to the lower BE values in the core-level signals of V 2p for the m&t-BiVO_4_/TiO_2_-NTAs-10 and m&t-BiVO_4_/TiO_2_-NTAs-20 samples compared with that of m&t-BiVO_4_/TiO_2_-NTAs-5, which is evidence of the existence of V^4+^ in BiVO_4_/TiO_2_-NTAs nano-heterostructures. Based on previous research reports [59,70], we are inclined to accept that the lower shift in the BE value for the XPS peak has an inevitable relationship with the presence of V_o_ defects, which is due to the changes in their local coordination environments and the increase in the electron density of the Bi and V atoms after introducing the V_o_. The further deconvolution of each asymmetric V 2p-core-level peak of all the specimens using Gaussian distribution peaks produces two doublets: the high-intensity doublet observed at the higher BE values, which was assigned to the V^5+^ state, and the low-intensity doublet at lower BE values, which indicated the presence of V^4+^ related to V_o_ defects in BiVO_4_ [71]. As demonstrated in Figure 6b, for the m&t-BiVO_4_/TiO_2_-NTAs-5 sample, besides the signals at 516.5 eV and 524.1 eV that index to the V 2p_3/2_ and V 2p_1/2_ peaks of V^5+^ [32], the signals sited at 516.2 eV and 523.4 eV confirm the presence of the V^4+^ valence state [72]. To further deconvolute the V 2p lines of the m&t-BiVO_4_/TiO_2_-NTAs-10 sample, the profiles of the V 2p_3/2_ doublet at the BE values of 515.5 eV and 516.3 eV can be assigned to the V^4+^ 2p_3/2_ and V^5+^ 2p_3/2_, respectively, and the peak of the V 2p_1/2_ core level has two components: V^5+^ 2p_1/2_ and V^4+^ 2p_1/2_, with the latter appearing at lower BE values, located at 523.8 eV and 523.1 eV [73,74], respectively. Eventually, each V 2p core-level signal for the m&t-BiVO_4_/TiO_2_-NTAs-20 sample is decomposed into V^4+^ and V^5+^ doublet peaks, exhibiting the V^4+^ 2p_3/2_ and V^4+^ 2p_1/2_ peaks centered at the BE values of 515.8 eV and 523.3 eV, respectively, and showing the V^5+^ 2p_3/2_ and V^5+^ 2p_1/2_ peaks at the BE values of 516.4 eV and 524.0 eV, respectively, which correspond to the V^4+^ and V^5+^cations in BiVO_4_ [75,76], respectively. Additionally, the m&t-BiVO_4_/TiO_2_-NTAs nano-heterojunctions are oxygen-deficient through the electroneutrality principle, and the V^4+^/V^5+^ molar ratios dictate the amount of nonstoichiometric oxygen, which is proportional to the ratio of the peak area of V^4+^/V^5+^ [77]. As depicted in Table 4, the calculated surface molar ratio of m&t-BiVO_4_/TiO_4_-NTA-10 had a higher ratio of V^4+^/V^5+^ (0.587) then that for BiVO_4_/TiO_4_-NTA-20 (0.491), and the lowest ratio was for BiVO_4_/TiO_4_-NTA-5 (0.436). 

To further verify the existence of V_o_ defects in the surface region of the as-prepared m&t-BiVO_4_/TiO_2_-NTAs nano-heterostructures, we analyzed the HR-XPS spectra of the O 1s core-level signals, and we present the results in Figure 6c. We deconvoluted all of the specimens into three components by Gaussian function fitting, indexing to three oxygen species: lattice oxygen (L_o_), V_o_, and adsorbed oxygen (A_o_), which was evidenced by the corresponding characteristic peaks at 529.9–530.0 eV, 530.5–531.1 eV, and 531.3–531.7 eV [78,79,80,81], respectively. In order to intuitively unveil the influence of the hydrothermal preparation environment on the number of V_o_ defects, we summarize the estimated V_o_/(L_o_ + A_o_) and A_o_/(L_o_ + V_o_) molar ratios of the O 1s XPS spectra for the m&t-BiVO_4_/TiO_2_-NTAs specimens with various hydrothermal synthesis times (5 h, 10 h, and 20 h) in Table 5, with the ratios of the peak area decomposed into three components: V_o_, L_o_, and A_o_. The maximal molar ratio value of V_o_/(L_o_ + A_o_) for m&t-BiVO_4_/TiO_2_-NTAs-10 is 0.571, followed by 0.402 for m&t-BiVO_4_/TiO_2_-NTAs-20, and the minimal value of 0.361 for m&t-BiVO_4_/TiO_2_-NTAs-5. Simultaneously, the molar ratio values of A_o_/(L_o_ + V_o_) evinced a similar varied trend, with that of V_o_/(L_o_ + A_o_) for the TiO_2_-NTAs hydrothermal precipitation BiVO_4_ NPs with times of 5 h, 10 h, and 20 h equaling 0.336, 0.423, and 0.396, respectively, which substantiates the amount of A_o_ species directly proportional to the V_o_ levels. According to a synthetic comparison of the above results, the higher surface V^4+^/V^5+^ molar ratio for the as-formed m&t-BiVO_4_/TiO_2_-NTAs samples contain a higher amount of V_o_ defects, and we observed a greater lower shift in the XPS peak, which was also confirmed by the molar ratio of A_o_/(L_o_ + V_o_) for the as-prepared samples, which mainly resulted from the chemisorption of the A_o_ species at the surface V_o_ defects of BiVO_4_ [81]. As expected, the concentration of V_o_ defects in the dual m&t-BiVO_4_/TiO_2_-NTAs heterostructure nanosystem was mediated by the synergistic effect of the precipitating time and pH value of the precursor. With the increase in the hydrothermal reaction time from 5 h to 20 h, the number of V_o_ defects gradually increased and then decreased, instead of showing nonlinear variation, and we found the maximal content of V_o_ defects in m&t-BiVO_4_/TiO_2_-NTAs-10. Hence, we conclude that the pH value of the precursor primarily controls the concentration of the V_o_ defects in the m&t-BiVO_4_/TiO_2_-NTAs heterostructure nanosystem.

**Figure 7:**
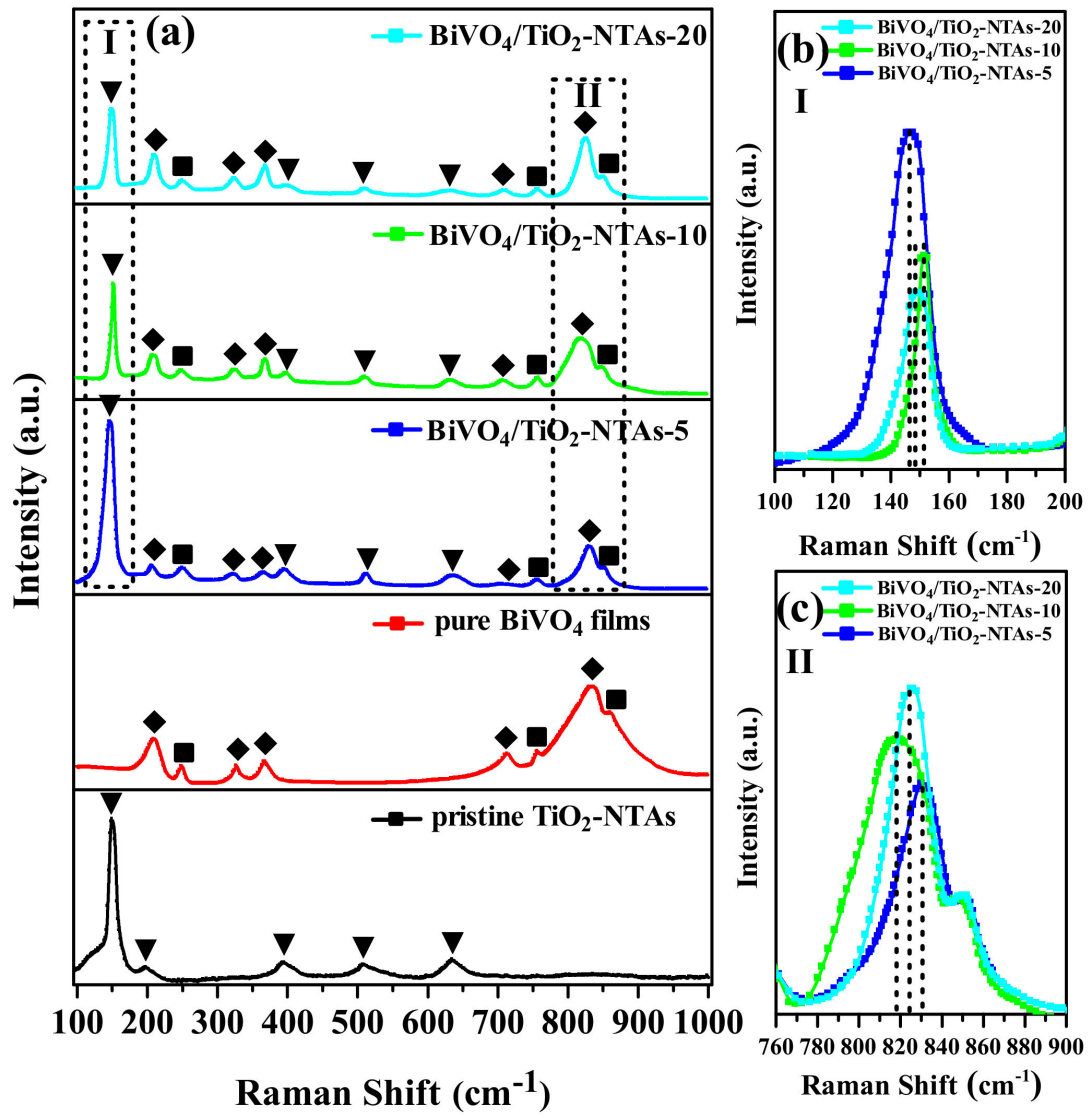
Raman spectra of (**a**) pristine TiO_2_-NTAs, pure BiVO_4_ films, and m&t-BiVO_4_/TiO_2_-NTAs nanoheterostructures with different BiVO_4_ hydrothermal depositing contents; (**b**) magnified spectra of the 100–200 cm^−1^ region, and (**c**) enlarged view of the 760–900 cm^−1^ range, respectively.

Raman spectroscopy is a powerful technique that can detect vibrational transitions, the bounding states in crystals, and the local structure distortions of inorganic materials. Accordingly, we obtained Raman spectra to evaluate the detailed structural and composition insights for the m&t-BiVO_4_/TiO_2_-NTAs nanohybrids synthesized by the hydrothermal-precipitation method with various reactive times using a green laser (532 nm), as sketched in Figure 7.

In Figure 7a, we depict the Raman patterns of the selected samples, the peaks of which are located within the scope of 100–1000 cm^−1^. In the given Raman spectrum of the pristine TiO_2_-NTAs, we identified a dramatically strong Raman peak at 149.6 cm^−1^, which corresponded to the E_1g_ vibrational mode, and a lower intense peak around 197.7 cm^−1^, which indexed to the main E_1g_ active mode of TiO_2_. We could assign the other three medium-intensity peaks located at 397.8 cm^−1^, 513.8 cm^−1^, and 639.0 cm^−1^ to the B_1g_ (A_1g_ + B_1g_) and E_1g_ vibrational modes [82], respectively. Anatase phase TiO_2_ was indicated by the presence of these Raman peaks, labeled by the symbol “▼”, which is consistent with the XRD analysis. The Raman spectrum analysis of the pure BiVO_4_ film as a reference exhibited the presence of eight typical vibrational bands at 210.9 cm^−1^; 248.6 cm^−1^; 326.5 cm^−1^; 367.3 cm^−1^; 711.2 cm^−1^; 758.3 cm^−1^; 821.7 cm^−1^; 855.6 cm^−1^, which are characteristic of mixed BiVO_4_ phases with ms-BiVO_4_ (marked as “♦”) and tz-BiVO_4_ (denoted with “■”), and which confirm the XRD results [83]. Specifically, we observed the external twisting vibrational modes in the pure BiVO_4_ at 210.9 cm^−1^ and 248.6 cm^−1^, corresponding to the formation of monoclinic and tetragonal phases, respectively, which are assigned to the translation/rotation and the Bi–O stretching modes, respectively, while those at 326.5 cm^−1^ and 367.3 cm^−1^ could be ascribed to the asymmetric (B_g_ symmetry mode) and symmetric (A_g_ symmetry mode) bending modes of the V–O bond in the VO_4_ units for the ms-BiVO_4_ phase [37], respectively. Likewise, the pure BiVO_4_ materials exhibited Raman bands at 711.2 cm^−1^ and 821.7 cm^−1^ and were assigned to antisymmetric stretching (B_g_ symmetry mode) and symmetric stretching (A_g_ symmetric mode) of the two sets of the V–O vibration bond of the monoclinic BiVO_4_ phase, respectively. Furthermore, the B_g_ stretching mode of V–O for the Raman peak sited at 711.2 cm^−1^ is related to the V_o_ defects [84], which is in good agreement with the UV–vis DRS test. The antisymmetric stretching vibration mode and symmetric bending vibration mode of the V–O bond in the tetragonal phase are indicated by the Raman bands at 758.3 cm^−1^ and 855.6 cm^−1^, respectively [83]. The Raman spectra of the hydrothermal-synthesized m&t-BiVO_4_/TiO_2_-NTAs nano-heterojunctions adopted the TiO_2_-NTAs decorated with various BiVO_4_ deposition times (5 h, 10 h, and 20 h) (Figure 7), which can be distinctly observed in the differences in the Raman patterns between them, which we categorized into four main groups, as follows: (I) Besides the monoclinic and tetragonal phases of BiVO_4_, we observed the Raman characteristic peaks of the anatase TiO_2_-NTAs in all three selected specimens, validating the predictions for the m&t-BiVO_4_/TiO_2_-NTAs mixed-phase nano-heterostructures, which coincide with the UV–vis DRS and XRD experiments. However, the intensities of the Raman peaks for the anatase TiO_2_ NTAs located at 149.6 cm^−1^, 397.8 cm^−1^, 513.8 cm^−1^, and 639.0 cm^−1^ gradually decrease when increasing the hydrothermal-reactive times from 5 h to 20 h, which is possibly because the deposited BiVO_4_ NPs attenuated the Raman signal of the underlying TiO_2_-NTAs [35]. (II) The peak intensities of the monoclinic phase (i.e., 210.9 cm^−1^, 326.5 cm^−1^, 367.3 cm^−1^, and 821.7 cm^−1^) increased with the increase in the pH values of the precursor from 2 to 8, while the intensities of the weak peaks for the tetragonal phase decreased with increasing pH values, which demonstrates that the content of m-BiVO_4_ increases with the increase in the pH value, and the variation trend of that for t-BiVO_4_ is the opposite, which corroborates that the pH value of the precursor has a substantial influence on the m-BiVO_4_ and t-BiVO_4_ contents in m&t-BiVO_4_/TiO_2_-NTAs nanocomposites, and which is in accordance with the results of the XRD detection. (III) As shown in Figure 7b, the enlarged view of the E_1g_ active vibration peaks of the anatase TiO_2_ (corresponding to Region I) centered at 147.2 cm^−1^, 151.5 cm^−1^, and 148.6 cm^−1^ for the m&t-BiVO_4_/TiO_2_-NTAs-5, m&t-BiVO_4_/TiO_2_-NTAs-10, and m&t-BiVO_4_/TiO_2_-NTAs-20 specimens, respectively, allow us to discern that the increased deposition of BiVO_4_ results in varied shift values to higher wavenumbers in comparison with m&t-BiVO_4_/TiO_2_-NTAs-5, and especially for the m&t-BiVO_4_/TiO_2_-NTAs-10 with the maximum value, which originates from the generation of V_o_ defects in BiVO_4_ caused by the deformation of the TiO_2_ lattice after its modification following the introduction of BiVO_4_ NPs [85]. (IIII) Additionally, Figure 7c is the magnified view of Region II in Figure 7a, and the range is between 760 cm^−1^ and 900 cm^−1^. It explicitly portrays that the Raman peaks of the A_g_ symmetric stretching modes for the m&t-BiVO_4_/TiO_2_-NTAs-10 and m&t-BiVO_4_/TiO_2_-NTAs-20 specimens are broader and shift to lower wavenumbers in comparison with those of m&t-BiVO_4_/TiO_2_-NTAs-5, and the shifts observed for m&t-BiVO_4_/TiO_2_-NTAs-10 are the most pronounced and are ascribed to the increase in the V–O bond length owing to the introduction of V_o_ in BiVO_4_ [86,87], which is completely consistent with the XPS experimental results.

**Figure 8:**
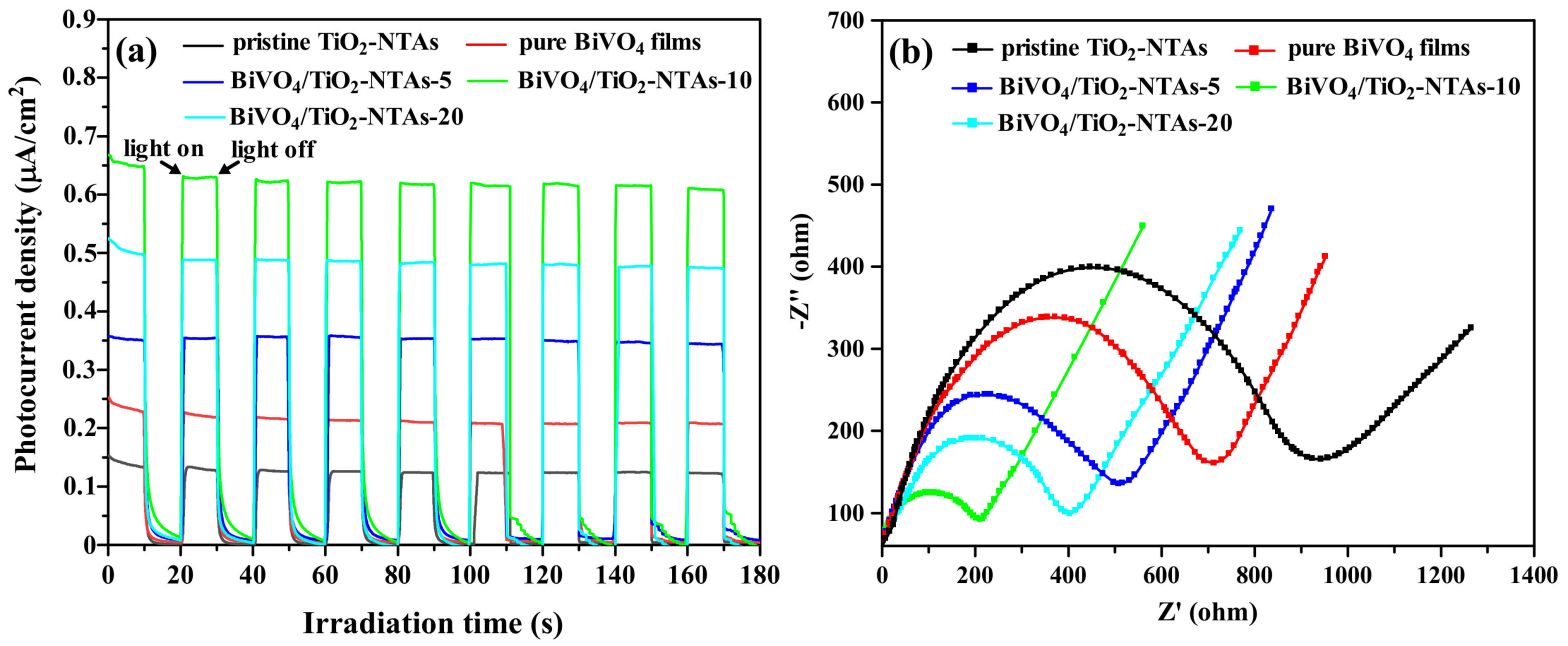
Transient I-t curves for (**a**,**b**) EIS of pure TiO_2_-NTAs, solely BiVO_4_ films, and dual nano-sized m&t-BiVO_4_/TiO_2_-NTAs nanohybrids deposited BiVO_4_ under different prepared times 5 h, 10 h, and 20 h irradiated by AM 1.5G simulated solar light, respectively.

To further evaluate the role of the m&t-BiVO_4_ decoration and V_o_ defects in the charge separation, migration, and recombination of the photoexcited e^−^–h^+^ pairs at the heterointerface between the m&t-BiVO_4_/TiO_2_-NTAs photoabsorber layer and electrolyte, we assessed the PEC characteristics, including the transient I–t curves and EIS of the primary and binary specimens, to explore the photocatalytic mechanism. We present the data in Figure 8a,b. A comparison of the transient photocurrent magnitude is thus a useful technique to demonstrate the m&t-BiVO_4_/TiO_2_-NTAs heterojunction photoactivity in response to the hydrothermal treatment under different reactive times. We noted the product’s photoresponse switching behavior over the course of nine chopped photoswitching cycles at an interval of 10 s under simulated solar light irradiation, and we present the results in Figure 8a. The photocurrent values of the as-obtained specimens in the order of pristine TiO_2_-NTAs < pure BiVO_4_ films < m&t-BiVO_4_/TiO_2_-NTAs-5 < m&t-BiVO_4_/TiO_2_-NTAs-20 < m&t-BiVO_4_/TiO_2_-NTAs-10 indicates the higher separation efficiency and longer charge carrier lifetime in the binary hetero-nanohybrids than in the single semiconductor. The pristine TiO_2_-NTAs had a weak photocurrent intensity (ca. 0.146 μA cm^−2^) owing to the wide E_g_, in which there was a limited photoresponse, while the pure BiVO_4_ revealed a higher photocurrent response (ca. 0.243 μA cm^−2^) than the primary TiO_2_-NTAs, with the on/off switch benefiting from the narrower E_g_ corresponding to greater visible-light absorption. The current density dramatically increased once the BiVO_4_ and TiO_2_ NTAs were fabricated into a heterojunction. The m&t-BiVO_4_/TiO_2_-NTAs-5 and m&t-BiVO_4_/TiO_2_-NTAs-20 samples had more sensitive photocurrent responses compared with the pure BiVO_4_ and TiO_2_-NTAs, approximately equaling 0.349 μA cm^−2^ and 0.503 μA cm^−2^, respectively, which were about 2.4 and 3.4 times more than that of the pure TiO_2_-NTAs sample, respectively. The m&t-BiVO_4_/TiO_2_-NTAs-10 sample expressed the highest photocurrent density, reaching about 0.646 μA cm^−2^, which was about 4.4 times that of the pure TiO_2_-NTAs. Simultaneously, according to the study of m&t-BiVO_4_/TiO_2_-NTAs-10, the light caused a spike in the data, which was due to the transient accumulation of photoinduced charges, which suggests that many carriers are produced in the heterojunctions rather than recombination. 

As exhibited in Figure 8b, the Nyquist plot for an EIS measurement typically consists of a series of semicircular arcs at high frequencies, and a linear portion at low frequencies. The resistance to charge separation is represented by the diameter of the semicircle, with a smaller arc radius signifying a greater efficiency of the photoinduced carrier separation. The pristine TiO_2_-NTAs sample had the greatest impedance arc radius compared with the other specimens, which suggests the greatest charge transfer resistance in all the selected samples, which is probably due to the poor light sensitivity in the visible-wavelength region for the TiO_2_-NTAs, which reduces the electron conduction rate. In comparison with the pure TiO_2_-NTAs, the arc radius of the pure BiVO_4_ films was further reduced, manifesting the more effective generation and separation of the photogenerated e^−^–h^+^ pairs within the scope of the simulated solar spectrum, which coincides quite well with the results of the UV–vis DRS and transient I–t analyses. The semicircular diameters of all these samples are ranked as follows: pristine TiO_2_-NTAs > single BiVO_4_ films > m&t-BiVO_4_/TiO_2_-NTAs-5 > m&t-BiVO_4_/TiO_2_-NTAs-20 > m&t-BiVO_4_/TiO_2_-NTAs-10, which is consistent with the variation trend of the abovementioned photocurrent density. The construction of an m&t-BiVO_4_/TiO_2_-NTAs nano-heterojunction provides an effective way to transport electrons to the charge collector and separate charges at the electrode/electrolyte interfaces. Strikingly, for the m&t-BiVO_4_/TiO_2_-NTAs nanohybrids, the arc radii of the curves decreased as the amount of BiVO_4_ increased (from 5 h to 10 h), and then began to increase when the BiVO_4_ deposition time reached 20 h. The smallest radius is seen in the Nyquist curve for BiVO_4_/TiO_2_-NTAs-10, which indicates the appropriate amount of BiVO_4_ NPs required to improve the conductivity and interfacial CT. If too much BiVO_4_ is deposited, then this will hinder the CT process for m&t-BiVO_4_/TiO_2_-NTAs nanocomposites. Hence, the diverse enhancement of the PEC performances are dependent on the different concentrations of V_o_ defects in m&t-BiVO_4_/TiO_2_-NTAs nanohybrids, which increases the carrier concentration and the transport of electrons by these channels, which allows for the effective separation of electron–hole pairs, as well as the spontaneous reaction with electrolytes to serve as additional highly reactive sites [88].

**Figure 9:**
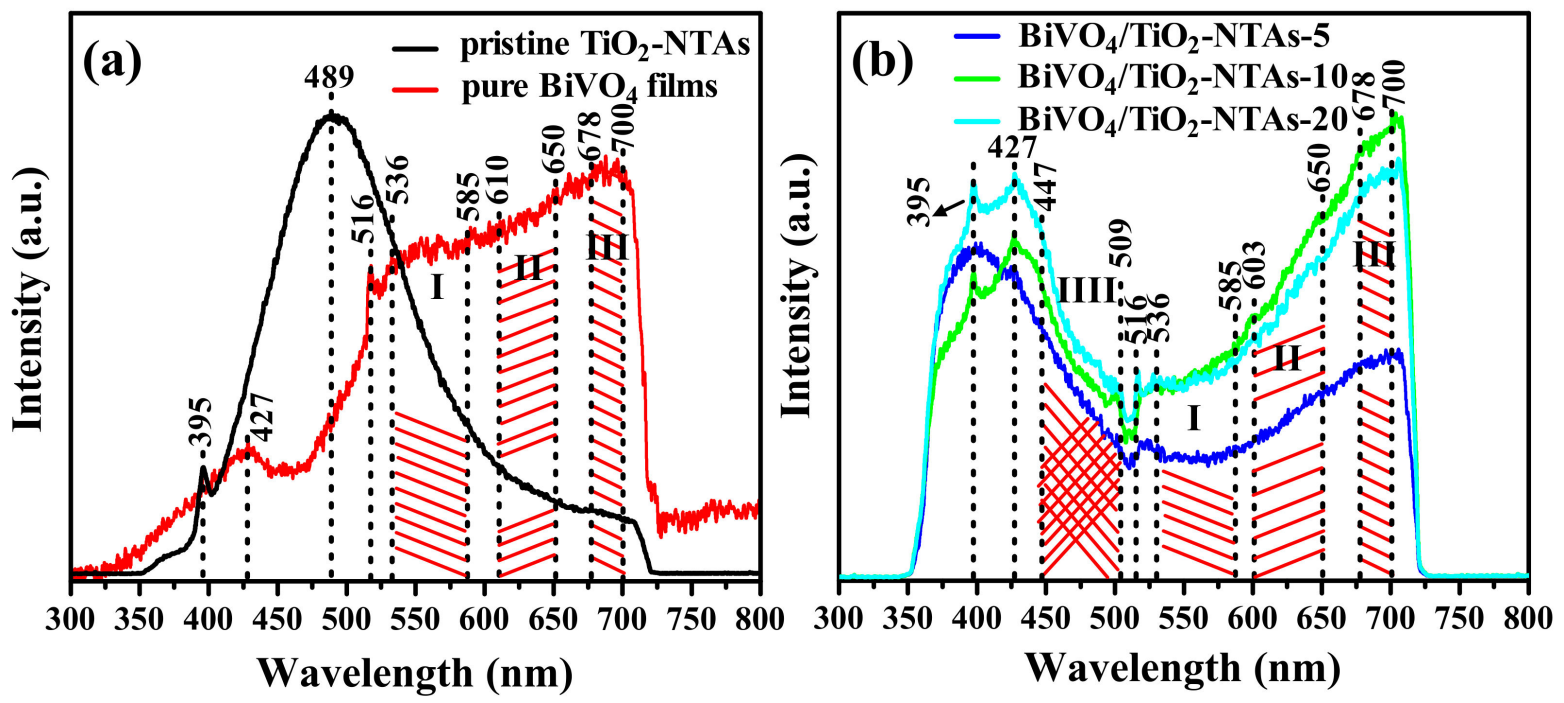
Steady-state PL spectra of bare TiO_2_-NTAs and pure BiVO_4_ films (**a**,**b**) m&t-BiVO_4_/TiO_2_-NTAs nanoheterojunctions with different depositing times of BiVO_4_ excited by 266 nm femtosecond pulse, respectively.

Steady-state PL spectroscopy is a broadly acknowledged channel to obtain additional insights into the electronic structure and properties of the active sites on the surfaces or interfaces of BiVO_4_/TiO_2_-NTAs nano-heterojunctions, whereby information such as the surface V_o_ and other defects, as well as the efficiency of the charge carrier trapping, migration, and recombination, can be provided. Compared with the change in the absolute intensity for the PL, we focus more on the variations in the steady-state PL spectral weight and features. We present the steady-state PL spectra for the pristine TiO_2_-NTAs, pure BiVO_4_ films, and binary BiVO_4_/TiO_2_-NTAs nano-heterojunctions with different BiVO_4_ NPs deposition amounts from 5 h to 20 h, excited by a 266 nm fs pulse at an ambient temperature, in Figure 9a,b. When in a steady state, the PL spectrum for pure TiO_2_ NTAs exhibits an asymmetric waveband emission plot, consisting of weak strength at 395 nm (3.1 eV) and an intense emission intensity at 489 nm (2.5 eV), which correspond to the NBE radiative transition [89] of the photogenerated carriers and the indirect radiative transition of the self-trapped electrons from V_o_ defects to holes in the TiO_2_-NTAs [90], respectively. Moreover, the steady-state PL pattern for the pure BiVO_4_ films were between 300 nm and 800 nm, with two emitted contributions sited at 427 nm (2.9 eV) and 516 nm (2.4 eV). Various authors have associated double emission peaks with the direct radiative recombination of carriers from the CB of V 3d to VB of O 2p and Bi 6s in VO_4_^3−^ for t-BiVO_4_ and m-BiVO_4_ [91,92], respectively. Simultaneously, there are three other successive PL irradiation domains: Region I (from 536 nm to 585 nm), Region II (from 610 nm to 650 nm), and Region III (from 678 nm to 700 nm), which originate from the indirect transition of the self-trapped electrons associated with the V_o_ defects, surface vanadium vacancies (V_v_), and V_o_ defect states with the holes in the VB of m&t-BiVO_4_ [86,93,94], respectively. Additionally, the steady-state PL spectra for the BiVO_4_/TiO_2_-NTAs binary nano-heterostructures with various BiVO_4_ deposition amounts were characterized with an acquisition time of 100 ms, and they also exhibited broad spectral emissions in the scope from 350 nm to 725 nm (Figure 9b). All the m&t-BiVO_4_/TiO_2_-NTAs nano-heterosystems expressed three emission peaks located at 395 nm, 427 nm, and 516 nm, which originated from the direct recombination of the carriers between the CB and VB for TiO_2_, t-BiVO_4_ and m-BiVO_4_, respectively, which are identical to the results in Figure 9a. Likewise, according to the differences in the origin of the PL spectra, the visible region can be divided into three parts: Region I (from 536 nm to 585 nm), Region II (from 603 nm to 650 nm), and Region III (from 678 nm to 700 nm), which originated from the indirect transition of the self-trapped carriers with the V_o_ and V_v_ defect states in m&t-BiVO_4_. Beyond that, we can clearly observe the steady-state PL band in the range of from 447 nm to 509 nm, labeled as Region IIII, which resulted from the indirect radiative transition between the trapped electrons in the V_o_ defect states and the holes in the VB of the TiO_2_-NTAs [95,96]. The PL intensity associated with the V_o_ defects in the m&t-BiVO_4_ increased with the increase in the BiVO_4_ deposition times from 5 h to 10 h, and then decreased when the BiVO_4_ deposition times were 20 h in Regions I and III, which is in accordance with the characterized results of the concentration for the V_o_ defects. As expected, the appearance of the PL intensity related to the V_v_ defects under 450 °C atmospheric annealing conditions without vanadium sources for all the tested [97] specimens manifested the coherent variation trend of the PL intensity for V_o_ defects, which is mainly attributed to the higher V_o_ concentration, which leads to a greater carrier density and the promotion of the generation of surface V_v_ defects (O_2_ + 2V^5+^ + 10e^−^ = > V_v_ + VO_2_) [98]. The additional V_v_ defects formed a series of discrete shallow defect levels in the bandgap of the m&t-BiVO_4_ photoelectrode, which can trap the photogenerated electrons and promote charge separation, which have positive effects on the PEC performance, as well as on the V_o_ defects [93,99]. Meanwhile, there was a significant steady-state PL band between 447 nm and 509 nm (Region IIII) that pertained to the photoinduced electron radiative recombination related to the V_o_ shallow trapping levels in the TiO_2_-NTAs [95,96], which is sensitive to the deposition amount of m&t-BiVO_4_.

**Figure 10:**
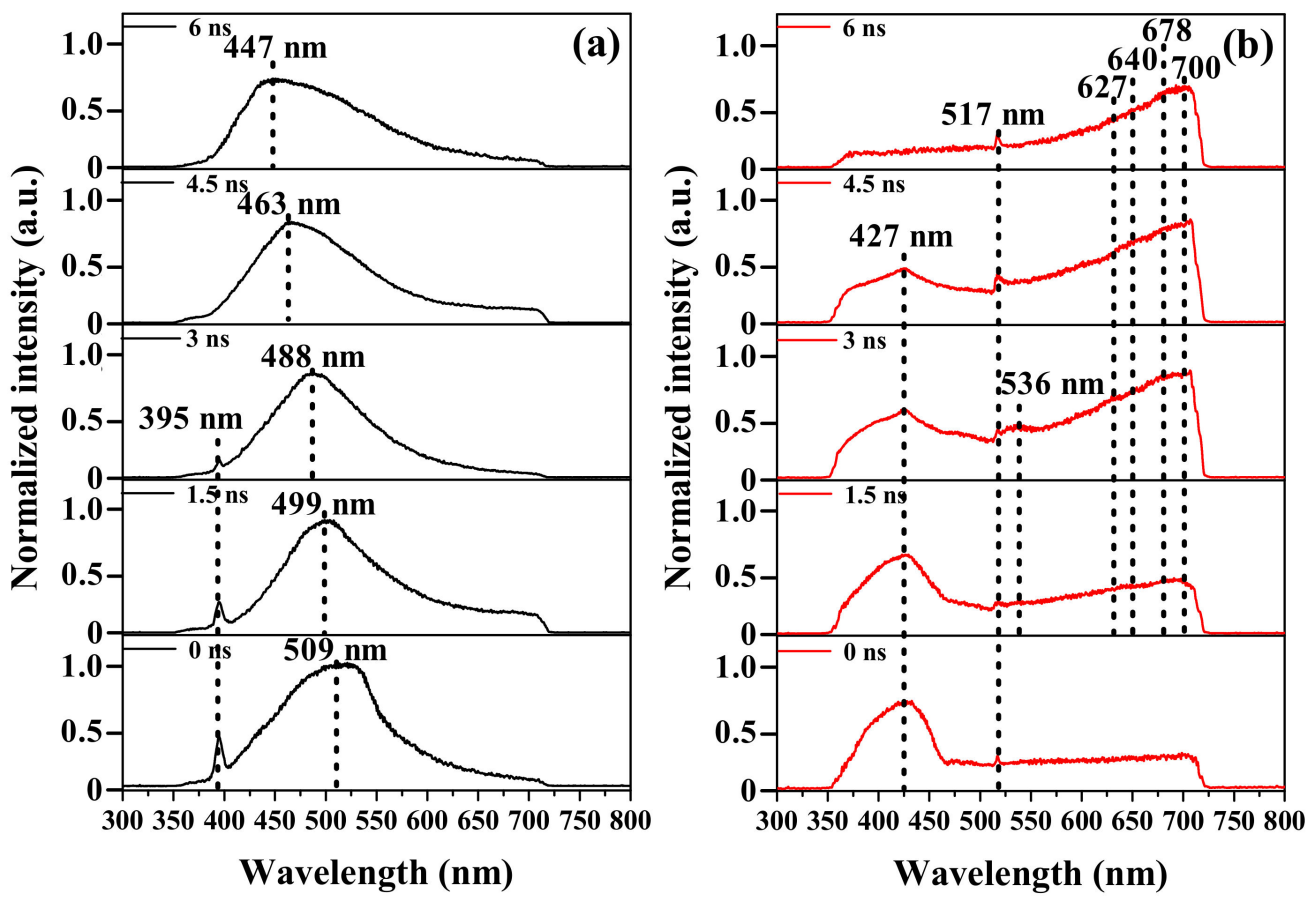
NTRT-PL spectra of the pure TiO_2_-NTAs (**a**,**b**) pure BiVO_4_ films irradiated by fs light with wavelength of 266 nm, respectively.

We present the NTRT-PL spectra for the pristine TiO_2_-NTAs and pure BiVO_4_ film specimens in Figure 10a,b. We irradiated these samples with a monochromatic fs laser wavelength at 266 nm under an atmospheric environment and normal temperature, with an interval time evolution of 1.5 ns.

The NTRT-PL patterns for the bare TiO_2_-NTAs sample, as plotted in Figure 10a, express a comparatively low transient PL emitted peak near 395 nm from 0 ns to 3 ns, which is ascribed to the direct radiative transition of the photoinduced carriers between the CB and VB in the TiO_2_ NTAs, as stated above [89]. The blueshift phenomenon for the transient PL emission peaks of the pure TiO_2_ NTAs emerged at 509 nm, 499 nm, 488 nm, 463 nm, and 447 nm with the increase in the intensities, and then decreased for a time evolution of 0–6 ns, originating from the indirect radiative emissions from the V_o_ defect levels within the VB of TiO_2_, which is in accordance with the PL earlier reported by other researchers [100]. Simultaneously, we could observe seven transient PL radiative peaks in the pure m&t-BiVO_4_ film sample, as revealed in Figure 10b, which were centered at 427 nm, 517 nm, 536 nm, 627 nm, 640 nm, 678 nm, and 700 nm, which is consistent with the results of the steady-state PL for the pristine BiVO_4_ presented in Figure 9a. As described above, we believe that the transient PL emission peaks sited at 427 nm and 516 nm are connected to the NBE direct recombination of t-BiVO_4_ and m-BiVO_4_, respectively. Likewise, the other transient PL radiative peaks centered at 536 nm, 627 nm, 640 nm, 678 nm, and 700 nm could be attributed to the indirect transition of the trapping carriers related to the V_o_ and V_v_ defects in the pure t&m-BiVO_4_ films.

**Figure 11:**
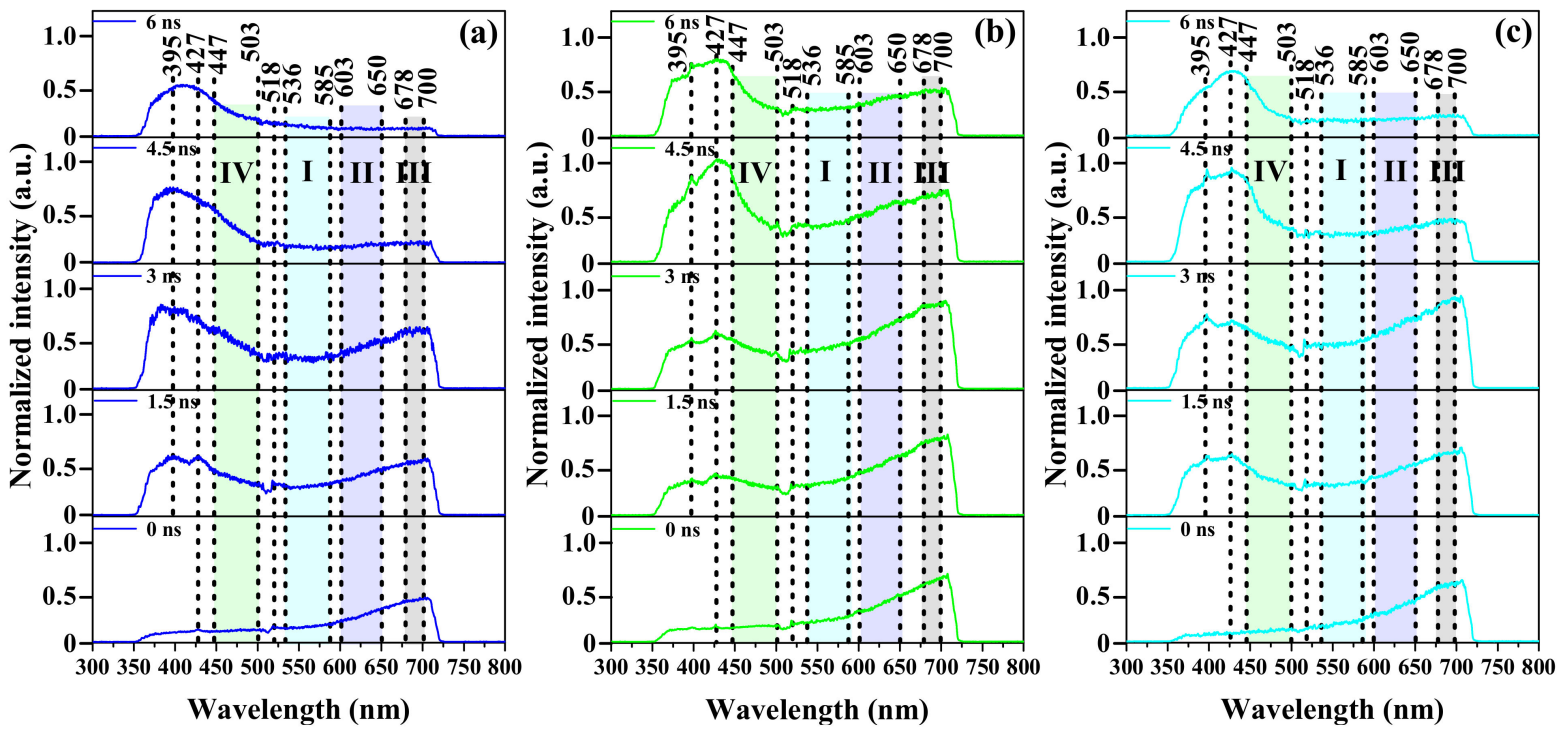
NTRT-PL spectra of the m&t-BiVO_4_/TiO_2_-NTAs nanoheterojunctions for BiVO_4_ NPs with different hydrothermal deposited times: (**a**) 5 h, (**b**) 10 h, and (**c**) 20 h under wavelength of 266 nm irradiation at atmospheric environment, respectively.

Ultrafast time-resolved PL spectroscopy is an authoritative indicator tool to track the CT dynamics. Stronger PL intensities represent higher concentrations of defect levels and holes associated with indirect and direct radiative recombination processes, respectively. We present the NTRT-PL spectra of the m&t-BiVO_4_/TiO_2_-NTAs nano-heterojunctions with various BiVO_4_ NP hydrothermal preparation times (5 h, 10 h, and 20 h) in Figure 11a–c. For the convenience of discussion, the NTRT-PL-wavelength classified regions agree with the steady-state PL, as discussed above. With the evolution of the time spent recording the spectral, we observed transient PL emission peaks in four different wavelength regions: Region I (from 536 nm to 585 nm); Region II (from 603 nm to 650 nm); Region III (from 678 nm to 700 nm); Region IV (from 447 nm to 503 nm). The as-prepared specimens exhibited Regions I–IV emissions that were unambiguously related to the indirect radiative transitions between the trapped electrons at the V_o_ and V_v_ defects in m&t-BiVO_4_, as well as to the indirect radiative emissions from the V_o_ defects in the TiO_2_ NTAs. These findings were consistent with the steady-state PL spectroscopy in Figure 9b. Additionally, the emitted PL peaks sited at 395 nm, 427 nm, and 518 nm can be assigned to the direct NBE transition of the m&t-BiVO_4_/TiO_2_-NTAs heterojunctions, which coincide with the results in Figure 10.

**Figure 12:**
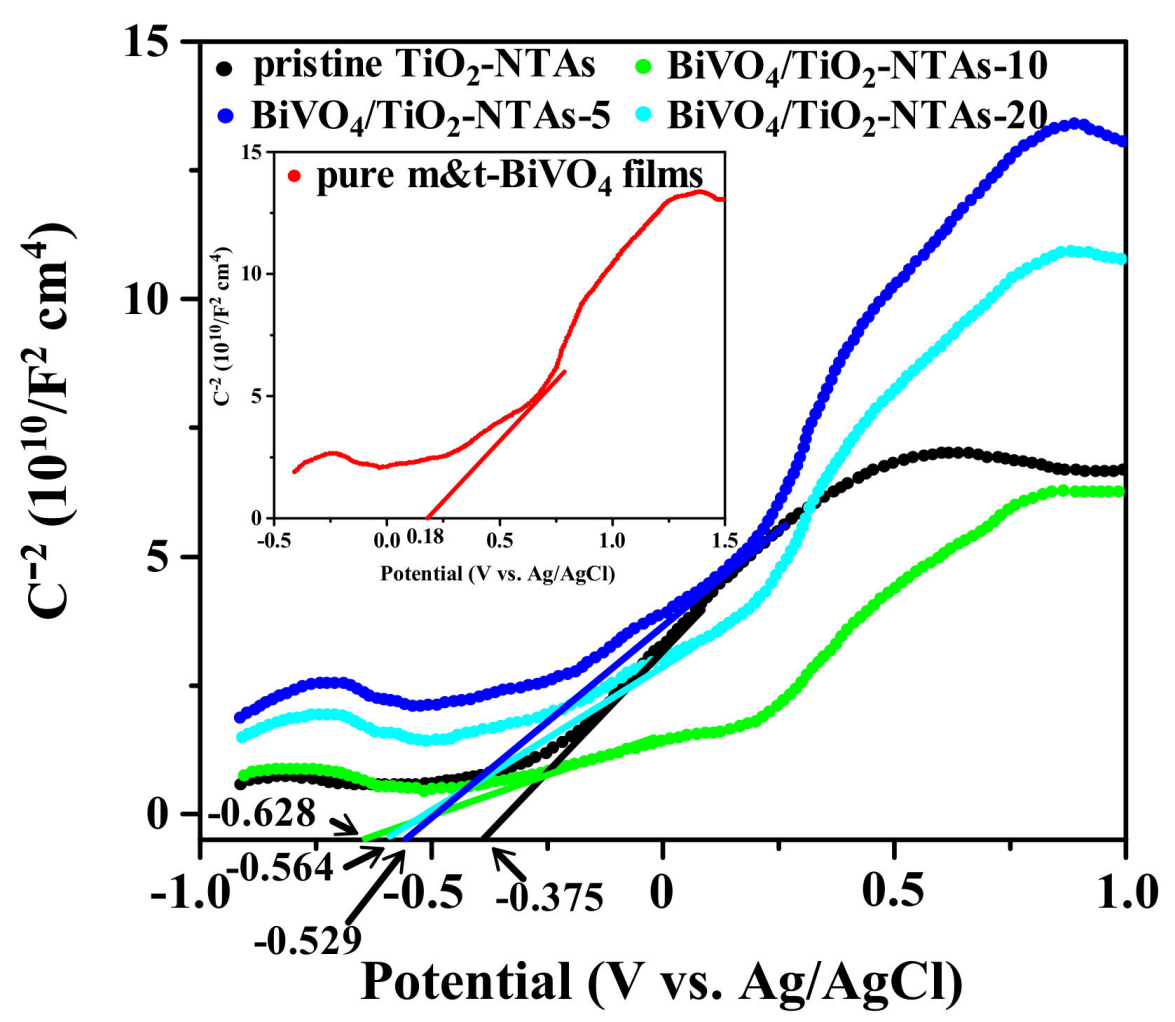
Mott-Schottky plots of the pristine TiO_2_-NTAs, pure m&t-BiVO_4_ films (inset), and as-obtained BiVO_4_/TiO_2_-NTAs nanocomposites with different BiVO_4_ hydrothermal precipitation times, respectively.

The information on the band-gap structure (i.e., CB, VB, and Fermi level (E_F_)) is essential to unveiling the interfacial CT mechanism between the m&t-BiVO_4_ and TiO_2_-NTAs nano-heterojunctions. To further dive into this issue, we subsequently conducted a Mott–Schoktty (M–S) analysis on the deliberated pristine TiO_2_ NTAs, pure m&t-BiVO_4_ films, and m&t-BiVO_4_/TiO_2_-NTAs nanohybrids, as plotted in Figure 12. We used the M–S formula (i.e., 1/C^2^ = (2/eε_0_ε_r_N_d_)(E-E_fb_-k_B_T/e)) [101] to assess the flat band potential (E_fb_) and density of the donor carriers (N_d_), where C and e are the differential capacitances of the Helmholtz layer and electron charge (1.602 × 10^−19^ C), respectively; ε_0_ is the permittivity of the vacuum (8.85 × 10^−12^ F m^−1^); ε_r_ is the relative permittivity (68 for BiVO_4_ and 170 for TiO_2_); E_fb_ is the hypothetical potential at which the semiconductor bands are flat and the band bending is zero, which is extrapolated from the 1/C^2^ axis in M–S plots; E is the applied electrode potential; k_B_ and T are the Boltzman constant (1.38 × 10^−23^ J K^−1^) and absolute temperature, respectively. Furthermore, we can calculate the N_d_ value with the following equation [102]: N_d_ = (2/eε_r_ε_0_) [d(1/C^2^)/dE]^−1^. The specimens were of the n type, with positive slopes for the M–S curves of 1/C^2^ versus the potential. We present the calculated values of the N_d_, E_fb_, and CB in Table 6.

As is evident, the values of the N_d_ for the samples of bare TiO_2_-NTAs, pure m&t-BiVO_4_ films, and m&t-BiVO_4_/TiO_2_-NTAs nano-heterojunctions with different BiVO_4_ amounts from 5 h to 20 h are 6.2 × 10^17^ cm^−3^, 3.3 × 10^18^ cm^−3^, 4.5 × 10^18^ cm^−3^, 7.6 × 10^18^ cm^−3^, and 6.6 × 10^18^ cm^−3^, respectively. The N_d_ values for the bare TiO_2_-NTAs and pure BiVO_4_ films are lower than that of the hydrothermal decorated TiO_2_-NTAs with BiVO_4_ NPs, which sufficiently validates that the donor density can be improved by a more powerful built-in electric field in the nano-heterojunction. The change trend is also similar to the results of the photocurrent densities and EIS, which substantially reduce the carrier recombination. Furthermore, the N_d_ values increased with the BiVO_4_-NPs hydrothermal reaction times, increasing from 5 h to 10 h, and then decreasing when a more compact BiVO_4_-NPs distribution deposition time was applied (20 h), validating that the V_o_ defects could boost the charge carrier densities and electrical conductivities in the m&t-BiVO_4_/TiO_2_-NTAs hetero-nanophotoanodes [98]. The number of active sites in BiVO_4_ constantly increases as a result of the growing trend of vacancy-state active sites with the increment in the BiVO_4_, and it decreases for the excess BiVO_4_ deposition mediated by pH values from 2 to 8. Additionally, the E_fb_ of the pristine TiO_2_-NTAs, pure m&t-BiVO_4_ films, and m&t-BiVO_4_/TiO_2_-NTAs nanohybrids with different BiVO_4_ amounts from 5 h to 20 h are as follows: −0.375 eV, 0.180 eV, −0.529 eV, −0.628 eV, and −0.564 eV vs. Ag/AgCl, respectively. Based on the relationship expression E_NHE_ = E_Ag/AgCl_ + 0.1976 (25 °C) [35], they are approximately −0.175 eV, 0.377 eV, −0.329 eV, −0.428 eV and −0.364 eV vs. NHE, respectively. Because the CB potential position (E_CB_) for the most n-type semiconductors is 0.1 eV higher than that of the E_fb_ [103], the E_fb_ value for the pristine TiO_2_ NTAs was −0.175 eV vs. NHE using the M–S plot, which completely coincides with the previously published literature [104]. The calculated value of the E_CB_ for the pure TiO_2_ NTAs was −0.275 eV, which is almost consistent with the −0.250 eV of the E_CB_ position reported by other researchers [105]. The V_o_ defects are thought to function as electron donors, increasing the potential height of the E_CB_ [106]. Moreover, the E_CB_ positions of the m&t-BiVO_4_/TiO_2_-NTAs heterostructure nanohybrids with different BiVO_4_ hydrothermal deposition times (5 h, 10 h, and 20 h) are approximately −0.429 eV, −0.528 eV, and −0.464 eV vs. NHE, respectively. The exposed surface V_o_ defects in the m&t-BiVO_4_ further strengthen the evidence that they act as electron donors, which could promote the electrical conductivity in BiVO_4_/TiO_2_-NTAs nano-heterojunctions. The presence of V_o_ defects is especially expected to shift the CB edge of m&t-BiVO_4_ towards the VB, resulting in an increase in the bandgap. This effect is caused by the alignment of the E_F_ between the m&t-BiVO_4_ and TiO_2_-NTAs, which increases the degree of the band bending at the interface between BiVO_4_ and TiO_2_-NTAs, which, in turn, facilitates the charge separation and transfer.

**Figure 13:**
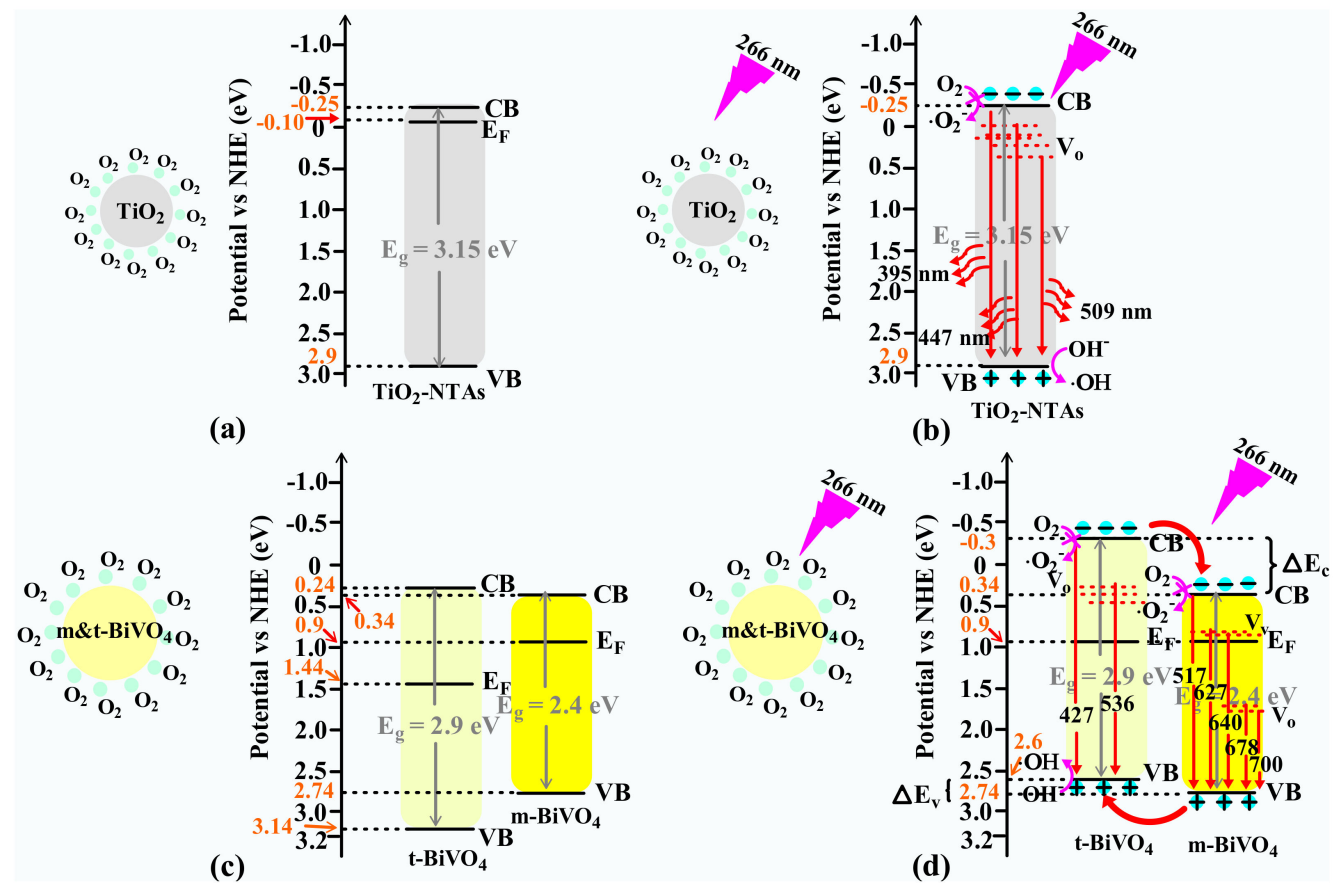
Diagrammatic sketchs of (**a**,**c**) are CB, VB, and E_F_ potential positions (vs. NHE) for single TiO_2_-NTAs and m&t-BiVO_4_ films before photo-irradiation conditions, respectively; Schematic diagrams (**b**,**d**) are photogenerated carriers generated, transferred, and recombination for pristine TiO_2_-NTAs and pure m&t-BiVO_4_ films in atmosphere irradiated by UVC light, severally.

On the basis of the above obtained experimental results of the NTRT-PL spectra and M–S plots, we present the mechanisms proposed to interpret the transient CT process for pristine TiO_2_ NTAs and pure m&t-BiVO_4_ films under fs laser irradiation at a wavelength of 266 nm at room temperature in Figure 13. Due to the absence of CT behavior before the formation of the photoexcited charge carriers, it is likely that the O_2_ was spontaneously attached on individual TiO_2_ NTAs and m&t-BiVO_4_, respectively. Additionally, the E_g_ for the pristine TiO_2_ NTAs is 3.15 eV, employing the aforementioned experimental results for the E_g_ using the Tauc plots in Figure 4b, and the values of the E_F_, E_CB_, and VB potential positions (E_VB_) for the TiO_2_ NTAs are −0.10 eV, −0.25 eV, and 2.9 eV versus the potential of normal hydrogen electrode (vs. NHE, E_NHE_) [105], respectively, which agrees with the M–S analysis and is depicted in Figure 13a. Simultaneously, the values of the E_CB_ and E_F_ for the t-BiVO_4_ and m-BiVO_4_ are 0.24 eV, 1.44 eV, 0.34 eV, and 0.9 eV vs. NHE without photoirradiation conditions, respectively, according to the previously mentioned research [105,107,108], while the E_VB_ edges of the t-BiVO_4_ and m-BiVO_4_ are 3.14 eV and 2.74 eV vs. NHE, respectively, using the formula E_VB_ = E_g_ − E_CB_, and the E_g_ values for them are 2.9 eV and 2.4 eV, respectively (Figure 13c).

In Figure 13b,d, we illustrate the processes of the generation, transfer, and radiative recombination of the photoexcited charge carriers in the TiO_2_ NTAs alone and pristine m&t-BiVO_4_ when exposed to 266 nm light. For the sample of undecorated TiO_2_ NTAs, the large number of electrons in the VB were excited to the CB by the incident photon energy, leaving behind holes in the VB of TiO_2_. This occurs when the photon energy (4.7 eV) is greater than the bandgap energy (3.15 eV) of TiO_2_-NTAs. At the initial time of UVC photoexcitation (denoted as t = 0 ns), the concentration of the e^−^_CB_ in the CB achieved the maximum value because there was no more generation of charges until the next cycle of light irradiation. We detected two transient PL peaks at 395 nm and 509 nm, which originated from direct and indirect radiative recombination, respectively, as illustrated in Figure 10a. As we have previously investigated [96,106], the V_o_ defect energy levels consist of a series of discrete levels that act as shallow donor levels slightly below the CB of anatase TiO_2_. The transient PL intensities centered at 499 nm, 488 nm, 463 nm, and 447 nm gradually decreased with the recording time from 1.5 ns to 6 ns, which was accompanied by a similar variation trend for the PL emitted peak centered at 395 nm, which we mainly ascribed to the direct and indirect carrier radiative recombination between the CB, V_o_ defects, and VB in the TiO_2_ NTAs. Based on previous reports [35], we believe that the probability of radiation from a shallow defect level is much greater than from a deep-trapping defect level, resulting in a blueshift of the transient PL peaks, which accords with the gradually decreased amount of e^−^_CB_, as exhibited in Figure 10a and Figure 13b. The atmosphere’s oxygen content cannot trap the CB from the TiO_2_-NTAs to generate superoxide radical anions ( O_2_^−^) because the E_CB_ level positions are more positive (−0.25 eV vs. NHE) than the redox potential of O_2_/O_2_^−^ (−0.33 eV vs. NHE) [24], which is an essential active oxygen species for impacting the PEC activity. The h^+^_VB_ in the VB can oxidize the OH^−^ into hydroxyl radical (OH) in the atmosphere, which is because the h^+^_VB_ level positions (+2.90 eV vs. NHE) are more positive than the redox potential of OH/OH^−^ (+1.99 eV vs. NHE) [109].

We present the energy-band diagram of the m-BiVO_4_/t-BiVO_4_ nano-heterojunction semiconductor after the thermodynamic equilibrium and irradiated by 266 nm fs light in Figure 13d. After the intimate contact between m-BiVO_4_ and t-BiVO_4_, the E_F_ of t-BiVO_4_ from 1.44 eV is 0.9 eV vs. NHE, and it was the same for the E_F_ of m-BiVO_4_. At the same time, the E_CB_ and E_VB_ of t-BiVO_4_ decreases from 0.24 eV to −0.30 eV and from 3.14 eV to 2.60 eV, respectively, and the establishment of an n–n junction at the interface creates an equilibrium electric field, which, in turn, generates an internal electric field. The m-BiVO_4_ energy band is decreased while the t-BiVO_4_ energy band is increased, which creates an equilibrium state in the nano-system. Hence, the type-II nano-heterojunction bandgap configuration results in a shift in the E_CB_ and E_VB_ of t-BiVO_4_ beyond those of m-BiVO_4_. The calculated CB offset (ΔE_c_) was 0.64 eV, and the VB offset (ΔE_v_) was 0.14 eV. When we irradiated the m-BiVO_4_/t-BiVO_4_ type-II nano-heterojunctions by 266 nm light, the electrons in the VB of the m&t-BiVO_4_ were inevitably excited to the CB with simultaneous generated holes in the VB owing to the fact that the radiated photon energy was larger than both the E_g_ values of the t-BiVO_4_ and m-BiVO_4_. At the end of fs light irradiation (t = 0 ns), the e^−^_CB_ concentration of the CB for the m&t-BiVO_4_ reached the maximum, spontaneously bringing about the NBE direct radiation recombination processes of the e^−^–h^+^ pairs, which could be responsible for the transient PL peaks sited at 427 nm and 517 nm. Additionally, Dai and Wang et al. [110,111] previously reported that the average lifetime (τ_e_) of charge carriers for t-BiVO_4_ is shorter than that of m-BiVO_4_, demonstrating τ_e_ values for t-BiVO_4_ and m-BiVO_4_ of 5.49 ns and 11.22 ns, respectively. The relation between the τ_e_ and the recombination probability is inversely proportional, and the direct radiative recombination probability for t-BiVO_4_ is much greater than that of m-BiVO_4_, which means that the NBE radiative PL intensity for t-BiVO_4_ (λ_PL_ = 427 nm) is higher than that of m-BiVO_4_ (λ_PL_ = 517 nm). With the evolution of the spectral recording time from 0 ns to 1.5 ns (t = 1.5 ns), the new transient radiative PL peaks emerged at 536 nm, 627 nm, 640 nm, 678 nm, and 700 nm, originating from the indirect radiative PL recombination between the e^−^_CB_ in the shallow trapping defect states and the VB of m&t-BiVO_4_. The concentration of the e^−^_CB_ for t-BiVO_4_ decreased when the irradiation time was increased from 1.5 ns to 3 ns (t = 3 ns). The ΔE_c_ should provide a facilitated way for the photogenerated e^−^_CB_ injection from the CB of t-BiVO_4_ to the CB of m-BiVO_4_, and the ΔE_v_ should promote the photogenerated h^+^_VB_ transfer from the VB of m-BiVO_4_ to the VB of t-BiVO_4_, resulting in an enhanced PL intensity of 536 nm and increased e^−^_CB_ concentration for m-BiVO_4_, which are responsible for the boosted PL strengths of the emitted wavelength sited at 627 nm, 640 nm, 678 nm, and 700 nm. Afterwards, we observed the gradually decreased transient-PL intensities for all of them with the evolution of the spectral recording time from 4.5 ns to 6 ns (t = 4.5–6 ns), which were mainly attributed to the continuous consumption for the e^−^_CB_ concentration in t-BiVO_4_ and m-BiVO_4_. The h^+^_VB_ in the VB of m&t-BiVO_4_ could convert the OH^−^ into an OH radical, benefitting from its E_VB_ potential positions that are sufficiently more positive (2.60 eV and 2.74 eV) than the redox potential of OH/OH^−^. The trapped O_2_ in the CB of m&t-BiVO_4_ could not be transformed into O_2_^−^, which was because the E_CB_-level positions were lower (−0.30 eV and 0.34 eV) than the redox potential of O_2_/O_2_^−^, as displayed in Figure 13d.

**Figure 14:**
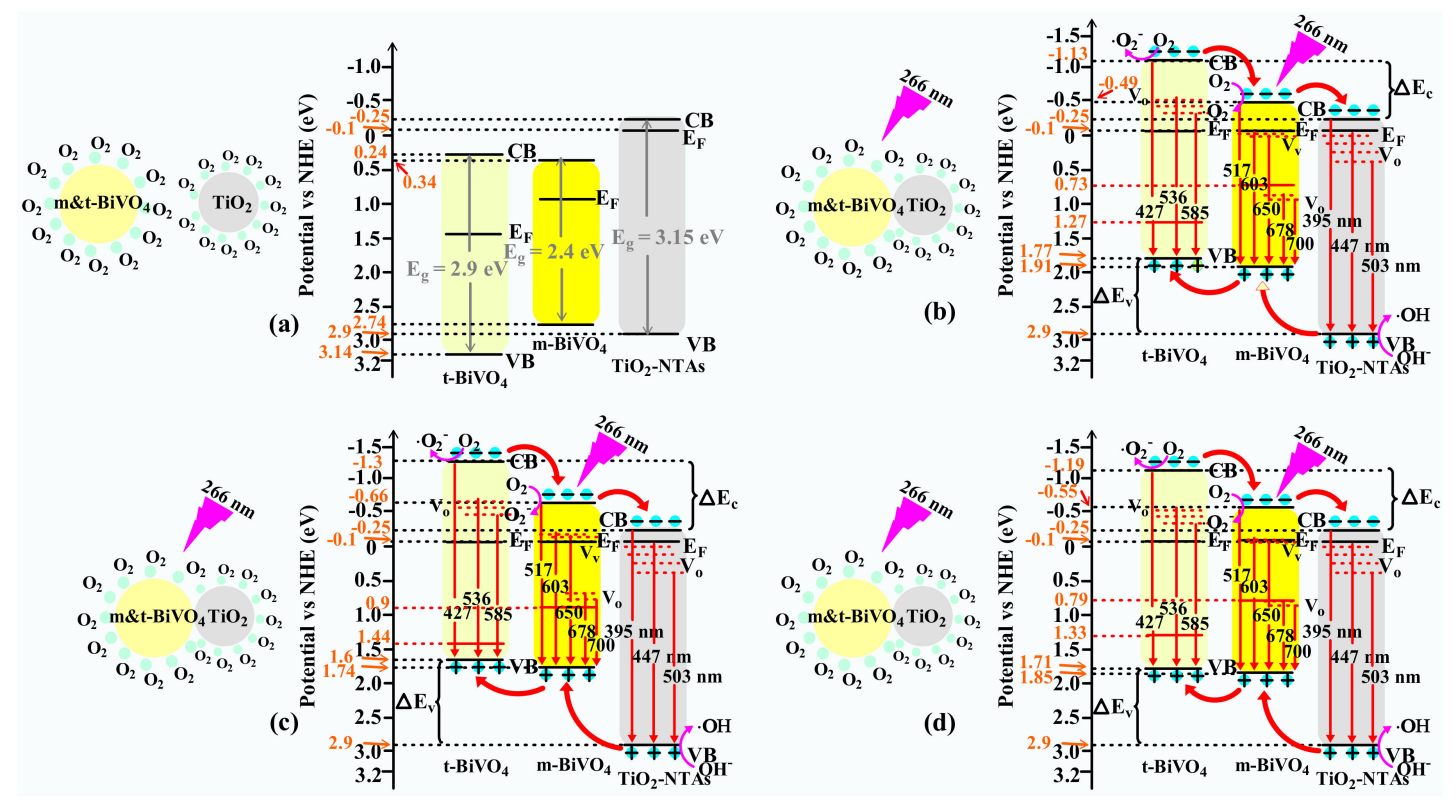
Diagrammatic sketchs of (**a**) CB, VB, and E_F_ potential positions (vs. NHE) for individual BiVO_4_ and TiO_2_-NTAs before contact; (**b**,**c**,**d**) are band-gap structure, charge carriers photo-excited and transient CT for BiVO_4_/TiO_2_-NTAs nanohybrids with various BiVO_4_ NPs hydrothermal-deposited amount before and after irradiated by UVC light, respectively.

We proposed the plausible kinetic process of interfacial CT in the binary BiVO_4_/TiO_2_-NTAs nano-heterostructures, which is dependent on the synergistic effect between the content ratio of the m&t-BiVO_4_ related to the hydrothermal deposition time and the amount of V_o_ defects mediated by the pH value, as we schematically illustrate in Figure 14.

We present the potential energy positions of the CB, VB, and E_g_ for the TiO_2_-NTAs and m&t-BiVO_4_ against NHE in Figure 14a. The specific potential energy values for these materials are similar to those seen in Figure 13. There is no CT process before the individual m&t-BiVO_4_ and pristine TiO_2_-NTAs contact, resulting in rather flat energy bands for the BiVO_4_ and TiO_2_-NTAs. We present the band configurations and schematic diagram of the generation, separation, and transport processes for the photogenerated charge carrier assembling of the m&t-BiVO_4_/TiO_2_-NTAs-5 nano-heterojunction before and after irradiation by 266 nm fs light in Figure 14b. Before light irradiation, the detailed potential energy positions of the E_F_ for the t-BiVO_4_ alone and m-BiVO_4_ were 1.27 eV and 0.73 eV (vs. NHE), respectively, which agrees with previous reports [25,112]. The CB positions of the single t-BiVO_4_ and m-BiVO_4_ are 0.24 eV and 0.34 eV, whereas the CB and E_F_ values for the TiO_2_ NTAs are −0.25 eV and −0.1 eV, respectively. Hence, we can deduce the VB potential positions for the t-BiVO_4_, m-BiVO_4_, and TiO_2_-NTAs sited at 3.14 eV, 2.74 eV, and 2.9 eV, respectively, and the E_g_ values of the t-BiVO_4_, m-BiVO_4_, and TiO_2_ NTAs are 2.9 eV, 2.4 eV, and 3.15 eV, respectively. When the close contact between m&t-BiVO_4_ with a preparation time of 5 h and TiO_2_ NTAs, a t-BiVO_4_/m-BiVO_4_/TiO_2_-NTAs integrated nano-heterojunction barrier is formed at the interface between the BiVO_4_ and TiO_2_, owing to the alignment of their different E_F_ level positions, as stated above. When the thermodynamic equilibrium was established, the E_F_ values for the t-BiVO_4_ and m-BiVO_4_ shifted to become −0.1 eV, which was identical to the E_F_ level of the TiO_2_. Furthermore, the E_C_ and E_V_ potential positions for the t-BiVO_4_ increased from 0.24 eV to −1.13 eV and from 3.14 eV to 1.77 eV, respectively, while those for the m-BiVO_4_ increased from 0.34 eV to −0.49 eV and from 2.74 eV to 1.91 eV, respectively. Logically, the maximal energy difference values of the CB and VB between the t-BiVO_4_ and TiO_2_-NTAs are 0.88 eV and 1.13 eV, respectively, denoted as ΔE_c_ and ΔE_v_, respectively, which suggests the formation of an enhanced built-in electric field force on the interfaces between the m&t-BiVO_4_/TiO_2_-NTAs-5 nano-heterojunctions compared with the isolated t-BiVO_4_/m-BiVO_4_ type-II nano-heterostructures. We present a schematic diagram of the energy-band potential position for the m&t-BiVO_4_/TiO_2_-NTAs-10 specimen under dark conditions in Figure 14c. After the thermodynamic equilibrium, the E_F_ values for t-BiVO_4_ and m-BiVO_4_ were −0.1 eV, which was the same as the E_F_ level of the TiO_2_. Simultaneously, the E_C_ and E_V_ of the t-BiVO_4_ increased from 0.24 eV to −1.3 eV and from 3.14 eV to 1.6 eV, respectively, whereas those of the m-BiVO_4_ increased from 0.34 eV to −0.66 eV and from 2.74 eV to 1.74 eV, respectively, resulting from the E_F_ values for the t-BiVO_4_ and m-BiVO_4_, which were 1.44 eV and 0.9 eV, respectively. The ΔE_c_ and ΔE_v_ values for m&t-BiVO_4_/TiO_2_-NTAs-10 were 1.05 eV and 1.3 eV, respectively, which vividly demonstrates the construction of a powerful built-in electric field driven by the Coulomb repulsive force. Additionally, in Figure 14d, we exhibit the potential energy positions of the bandgap for the m&t-BiVO_4_/TiO_2_-NTAs-20 sample, tightly contacted and without light irradiation. The calculated E_c_ and E_v_ positions of the t-BiVO_4_ were −1.19 eV and 1.71 eV, respectively, while those of the m-BiVO_4_ were −0.55 eV and 1.85 eV, respectively, when the thermodynamic equilibrium was reached, originating from the E_F_ values of the t-BiVO_4_ and m-BiVO_4_, which were 1.33 eV and 0.9 eV moved towards −0.1 eV, respectively, which is in good agreement with the previous description on the variation in the work function with the V_o_ defect concentration [86]. As a consequence, the heterostructure alignment of m&t-BiVO_4_/TiO_2_-NTAs-20 with the extreme ΔE_c_ and ΔE_v_ between the t-BiVO_4_ and TiO_2_-NTAs are 0.94 eV and 1.19 eV, respectively. The ΔE_c_ and ΔE_v_ values increased with the increasing hydrothermally synthesized times of the BiVO_4_ NPs for the m&t-BiVO_4_/TiO_2_-NTAs nanohybrids from 5 h to 10 h, and then decreased when the BiVO_4_ deposition time was 20 h. As expected, the m&t-BiVO_4_/TiO_2_-NTAs-20 specimen exhibited the maximum values for the ΔE_c_ and ΔE_v_ among the as-prepared samples, implying that it is the most forceful supplement of the CT driving force, which is completely consistent with the variation trend of the V_o_ defect amount and the truth for the effective acceleration of the electron mobility. We illustrate and detail the typical CT pathway for t-BiVO_4_/m-BiVO_4_/TiO_2_-NTAs nano-heterojunctions with different BiVO_4_ NP hydrothermal preparation times (5 h, 10 h, and 20 h) under 266 nm light irradiation in ambient air in Figure 14b–d. In the circumstance that the exposed m&t-BiVO_4_/TiO_2_-NTAs nano-heterojunctions are irradiated by UVC light, the exposure of the t-BiVO_4_, m-BiVO_4_, and TiO_2_ semiconductors to photons with energies (ca. 4.7 eV) greater than the E_g_ threshold of each material causes a large number of electrons to be excited from the VB to the CB. This leaves behind h^+^ in the VB, which creates an e^−^–h^+^ pair. When the UVC light impulse is cut off, the m&t-BiVO_4_/TiO_2_-NTAs nanosystem no longer generates photoinduced e^−^–h^+^ pairs. Atmospheric O_2_ molecules can be adsorbed and activated by the V_o_ vacancy sites to produce reactive oxygen species ( O_2_^−^ and OH) (i.e., O_2_ + e^−^_CB_ → O_2_^−^, O_2_^−^ + 2e^−^_CB_ + 2H^+^ → OH + OH^−^) [113], and can also serve as CT channels to deplete the excessive e^−^_CB_ in the CB, which is ascribed to the E_CB_ level position of m&t-BiVO_4_, which is more negative than the reduction potential of O_2_/O_2_^−^ (−0.33 eV vs. NHE). Moreover, atmospheric OH^−^ in water molecules could be oxidized by the h^+^_VB_ in the VB of TiO_2_ to yield OH (OH^−^ + h^+^_VB_ → OH), benefiting from the E_VB_ potential position of the TiO_2_ NTAs (+2.9 eV vs. NHE), which is more positive than that of OH^−^/OH (+1.99 eV vs. NHE). The transient CT process between the t-BiVO_4_/m-BiVO_4_/TiO_2_-NTAs nano-heterojunctions introduces adequate V_o_ and V_v_ defects, which lead to large increases in the charge carrier concentrations and strong electronic perturbations around the vacancy defects [23,113], which induce upward shifts in the E_CB_ and E_VB_ potential sites and enable a large ΔE_c_ and ΔE_v_, which can speed up the migration of the photoexcited carriers. By combining the NTRT-PL spectra for the as-synthesized m&t-BiVO_4_/TiO_2_-NTAs specimens with varied BiVO_4_ NP deposition amounts in Figure 11a–c, we can see that there were almost identical transient PL peaks sited at 3.1 eV, 2.9 eV, and 2.4 eV, which resulted from the direct radiative recombination transition of the photogenerated carrier NBE between the CB and VB for TiO_2_, t-BiVO_4_, and m-BiVO_4_, respectively. Simultaneously, we could clearly discern four NTRT-PL bands, including Region I (from 2.31 eV to 2.12 eV), Region II (from 2.05 eV to 1.91 eV), Region III (from 1.83 eV to 1.77 eV), and Region IV (from 2.77 eV to 2.46 eV), which stemmed from the indirect radiative recombination transition of the self-trapped electrons with V_o_ and V_v_ defect states in m&t-BiVO_4_ and TiO_2_, as depicted in Figure 9. In the initial stage, a nanosystem is irradiated under 266 nm light (t = 0 ns), and plenty of e^−^_CB_ are photoexcited and accumulate in the CB of BiVO_4_, with simultaneous generated holes in the VB, owing to the absorption of most of the incident photons by the surface-covered BiVO_4_ NP films compared with the substrate of the TiO_2_ NTAs. Reasonably, the radiative peak intensities centered at 2.9 eV and in Region I increased with the evolution time increase from 0 ns to 3 ns. Because the band potentials of the m&t-BiVO_4_/TiO_2_-NTAs nanocomposites fit the requirements necessary to form a heterojunction with a straddling gap, the E_CB_ edge potential of t-BiVO_4_ are more negative than those of m-BiVO_4_ and TiO_2_, and the photogenerated high-energy electrons tend to transfer more freely from the CB of t-BiVO_4_ toward the CB of m-BiVO_4_ and TiO_2_ NTAs, stimulated by the built-in electric field force. Thus, the transient PL peak intensities located at 2.4 eV, Regions II and III, increased as the recording time increased from 0 ns to 3 ns. The increasing consumption of the photoinduced e^−^_CB_ in the CB of BiVO_4_, which resulted from the direct and indirect radiative recombination between the CB, vacancy defects, and h^+^_VB_ in the VB during the last stage of the spectral recording time (t = 4.5 ns–6 ns), gave rise to the attenuated transient PL intensities sited at 2.9 eV and 2.4 eV, Regions I–III. Besides the E_CB_ potential for t-BiVO_4_, the E_CB_ edge of m-BiVO_4_ also had a superior potential to that of the TiO_2_ NTAs, and hence, could supply minor electron resistance pathways compared with the single m&t-BiVO_4_ photoanode, representing the ΔE_c_ between the E_CB_ potential position for m-BiVO_4_ and that for TiO_2_, and could act as a secondary built-in electric field force driven by Coulomb repulsive force, which accelerated the charge carrier transfer rate and migration from the CB of m&t-BiVO_4_ to the adjacent. Rationally, the radiative peak intensities centered at 3.1 eV and in Region IV increased with the evolution time increase from 1.5 ns to 4 ns, while the transient PL peak intensities centered at 2.9 eV, 2.4 eV, Regions I–III, decreased at the NTRT-PL recording time which was 4 ns, originating from the elevated e^−^_CB_ concentration of the CB in TiO_2_, and the weakened e^−^_CB_ content in the CB of BiVO_4_, which was caused by the injection of photoproduced carriers from the CB of BiVO_4_ to that of TiO_2_ due to the formation of the m&t-BiVO_4_/TiO_2_-NTAs nano-heterojunctions. At the end stage of the spectra recording (t = 6 ns), the transient PL peak emission centered at 3.1 eV and in Region IV gradually declined, mainly agreeing with the drastic decrease in the e^−^_CB_ concentration in the CB of the TiO_2_ NTAs through PL radiative recombination, as depicted in Figure 11a–c. The NTRT-PL intensities of the as-synthesized m&t-BiVO_4_/TiO_2_-NTAs nano-heterojunctions increased with the increase in the hydrothermal deposition time from 5 h to 20 h, which is intimately connected to the concentration of the photogenerated charge carrier radiative recombination, which is dependent on the decorated amount of m&t-BiVO_4_ NPs. However, the m&t-BiVO_4_/TiO_2_-NTAs-10 specimen evinced the strongest transient PL intensity compared with those for all the other samples, which elucidates the concerted interaction between the deposited amount and the deposition time and V_o_ defect content mediated by the prepared pH value, which induced the discrepancy in the ΔE_c_/ΔE_v_ among the as-formed specimens, and which was identical to the 0.88 eV/1.13 eV, 1.05 eV/1.30 eV, and 0.94 eV/1.19 eV for the different BiVO_4_ hydrothermal deposition contents (5 h, 10 h, and 20 h, respectively).

**Figure 15:**
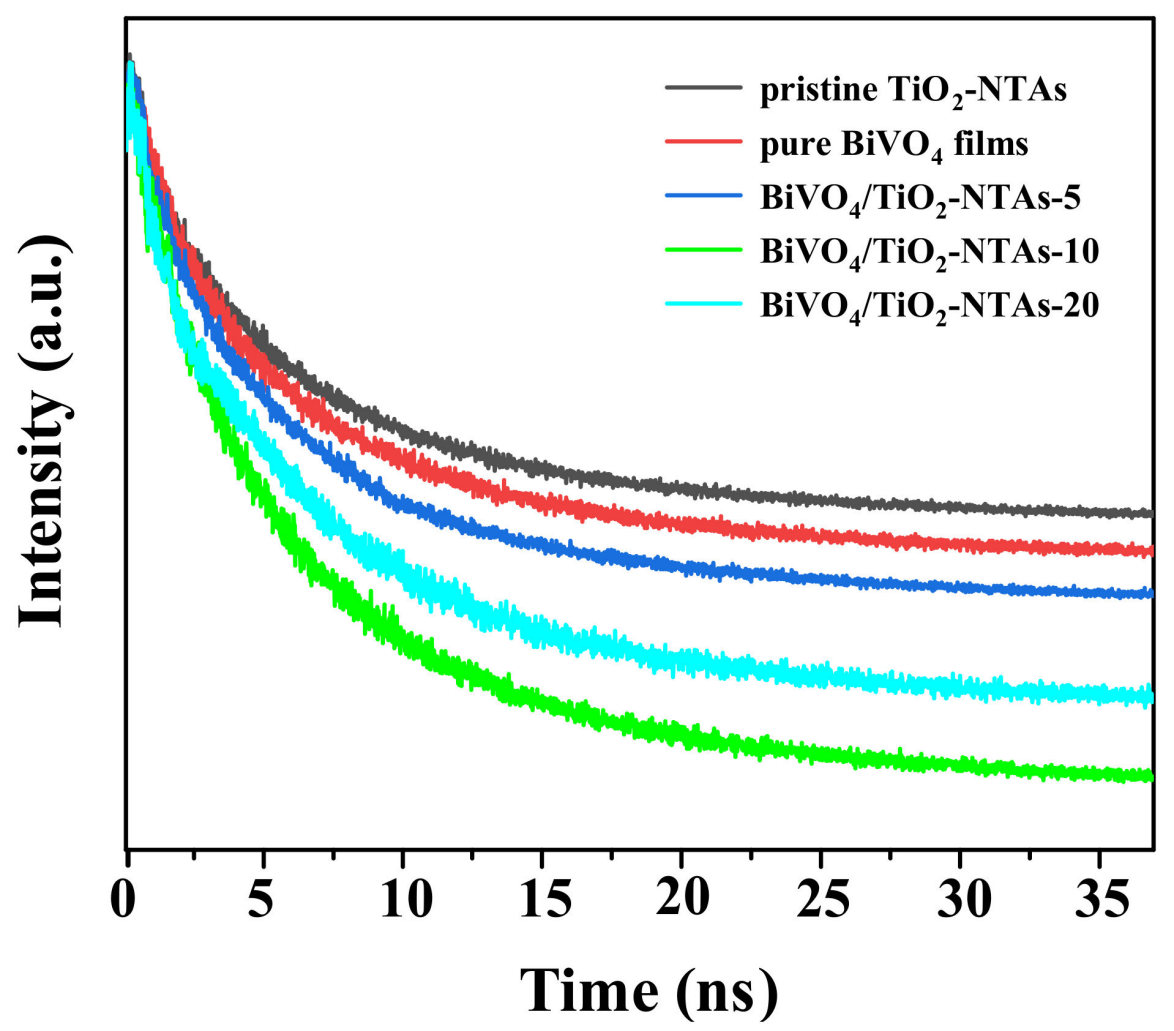
Time-resolved PL spectra of pure TiO_2_-NTAs, plain BiVO_4_ films, and m&t-BiVO_4_/TiO_2_-NTAs with various BiVO_4_ NPs hydrothermal-deposited times, respectively.

We recorded the PL decay profiles of the as-prepared specimens in Figure 15 by exciting the specimens with 375 nm laser pulses. We collected the PL decay trace at 678 nm (ca. 1.8 eV) for the plain BiVO_4_ film sample, and we conducted the other PL-decay traces at 447 nm (ca. 2.8 eV), which originated from the e^−^_CB_ trapped in the V_o_ defect indirect radiative recombination transition to the h^+^_VB_ in BiVO_4_ and TiO_2_, respectively. When irradiated by UVC light, the staggered band offset consequently induces a built-in electric field in the m&t-BiVO_4_ and m&t-BiVO_4_/TiO_2_-NTAs nano-heterojunction specimens, which drive the photogenerated electron injection into the CBs of BiVO_4_ and TiO_2_. These photogenerated e^−^_CB_ preferentially transfer to the V_o_ defect levels, which results in a substantial variation in the PL decay kinetics. By comparing the emission decay profiles for the pristine TiO_2_-NTAs, plain m&t-BiVO_4_ films, and TiO_2_-NTAs decorated with different BiVO_4_ NP amounts, we can obtain the penetrating information for interpreting the fate of the charge carriers between the relevant specimens.

The lifetime of a carrier can be probed from the TRPL spectrum, and it complies with the biexponential rate law: I(τ) = A_1_exp(−τ/τ_1_) + A_1_exp(−τ/τ_2_) [114], where τ_1_ and τ_2_ are the fast and slow components, respectively, which originate from defect-induced nonradiative recombination and radiative recombination, respectively. Both A_1_ and A_2_ correspond to the decay amplitude [115]. We used the formula τ_avg_ = (A_1_τ_1_^2^ + A_2_τ_2_^2^)/(A_1_τ_1_ + A_2_τ_2_) to calculate the average lifetime of a carrier (τ_avg_). As detailed in Table 7, the τ_avg_ values were 4.99 ns, 4.53 ns, 4.29 ns, 3.86 ns, and 4.06 ns for the pristine TiO_2_-NTAs, plain m&t-BiVO_4_ films, and m&t-BiVO_4_/TiO_2_-NTAs with BiVO_4_ NP hydrothermal preparation times of 5 h, 10 h, and 20 h, respectively. The magnitude order of the τ_avg_ for the as-obtained specimens accords well with that previously reported [116,117], and it has consistently corroborated the validity of the simplified kinetics model considered for the synergistic effect between the hydrothermal deposition content and V_o_ defect concentration in mediating the CT of m&t-BiVO_4_/TiO_2_-NTAs heterojunction nanohybrids. Evidently, all the specimens with the characteristic heterostructure indicated shortened τ_avg_ values relative to the pristine TiO_2_-NTAs and single BiVO_4_ films, and especially for the m&t-BiVO_4_/TiO_2_-NTAs sample with a deposition time of 10 h, which possessed the shortest τ_avg_ value (3.86 ns), intimately stemming from the highest band-offset values (ΔE_c_ and ΔE_v_), which suggests that the shorter lifetime reflects the higher carrier injection efficiency [114]. Interestingly, the two dominant benefits that are expected from BiVO_4_ NP incorporation (i.e., separation and fast charge transport) have compatible effects on the CT rate. We also analyzed the interfacial CT kinetics for the BiVO_4_/TiO_2_-NTAs type-II nano-heterojunctions, presuming that the heterojunction interfaces between BiVO_4_ and TiO_2_ were accountable for the observed reduced carrier lifetime. We can evaluate the CT rate constant (k_ct_) by the following equation: k_ct_ (∗ → TiO_2_) = 1/τ_avg_ (∗/TiO_2_) − 1/τ_avg_ (plain TiO_2_), where ∗ represents the BiVO_4_, which is the alternative semiconductor forming a heterostructure. The calculated k_ct_ values were 3.27 × 10^7^ s^−1^, 5.86 × 10^7^ s^−1^, and 4.59 × 10^7^ s^−1^ for m&t-BiVO_4_/TiO_2_-NTAs with diverse BiVO_4_ deposition times of 5 h, 10 h, and 20 h, respectively. The variation tendencies of the k_ct_ values were proportional to the changing trends of the VB offset values, which presented the driving force to promote the photogenerated h^+^_VB_ transfer from the VB of TiO_2_ to the VB of the adjacent BiVO_4_ because the carrier radiative lifetime of TRPL is directly dependent on the recombination lifetime of the minority carriers in nano-heterojunctions. Simultaneously, the k_ct_ value of the m&t-BiVO_4_/TiO_2_-NTAs-10 specimen was higher than that of the others, which indicates that the enlarged band offset (ΔE_c_ and ΔE_v_) associated with the V_o_ defect concentration induces a stronger built-in field force, which achieved the most effective charge spatial separation and active charge-transport injection of the photoinduced e^−^_CB_ − h^+^_VB_ pairs on the different sides of the m&t-BiVO_4_/TiO_2_-NTAs heterojunction, and promoted a great number of the e^−^_CB_ of TiO_2_ and h^+^_VB_ of BiVO_4_ to participate in the redox reaction.

We conducted photodegradation tests of the as-prepared nano-heterostructures to testify to the feasibility of the as-proposed transient CT mechanisms associated with the synergistic effect. We proposed the following chemical reactions:

TiO_2_-NTAs + hν → h^+^_VB_ (TiO_2_) + e^−^_CB_ (TiO_2_) (5)

m&t-BiVO_4_ + hν → h^+^_VB_ (BiVO_4_) + e^−^_CB_ (BiVO_4_) (6)

m&t-BiVO_4_/TiO_2_-NTAs + hν → e^−^_CB_ (BiVO_4_/TiO_2_) + h^+^_VB_ (BiVO_4_/TiO_2_) (7)

e^−^_CB_ + O_2_ → O_2^−^_ (8)

O_2^−^_ + H^+^ → HO_2_ (9)

e^−^_CB_ + H^+^ + HO_2_ → H_2_O_2_ (10)

H_2_O_2_ + e^−^_CB_ → OH + OH^−^ (11)

O_2^−^_, OH, h^+^_VB_ + MO → degradation products (12)

**Figure 16:**
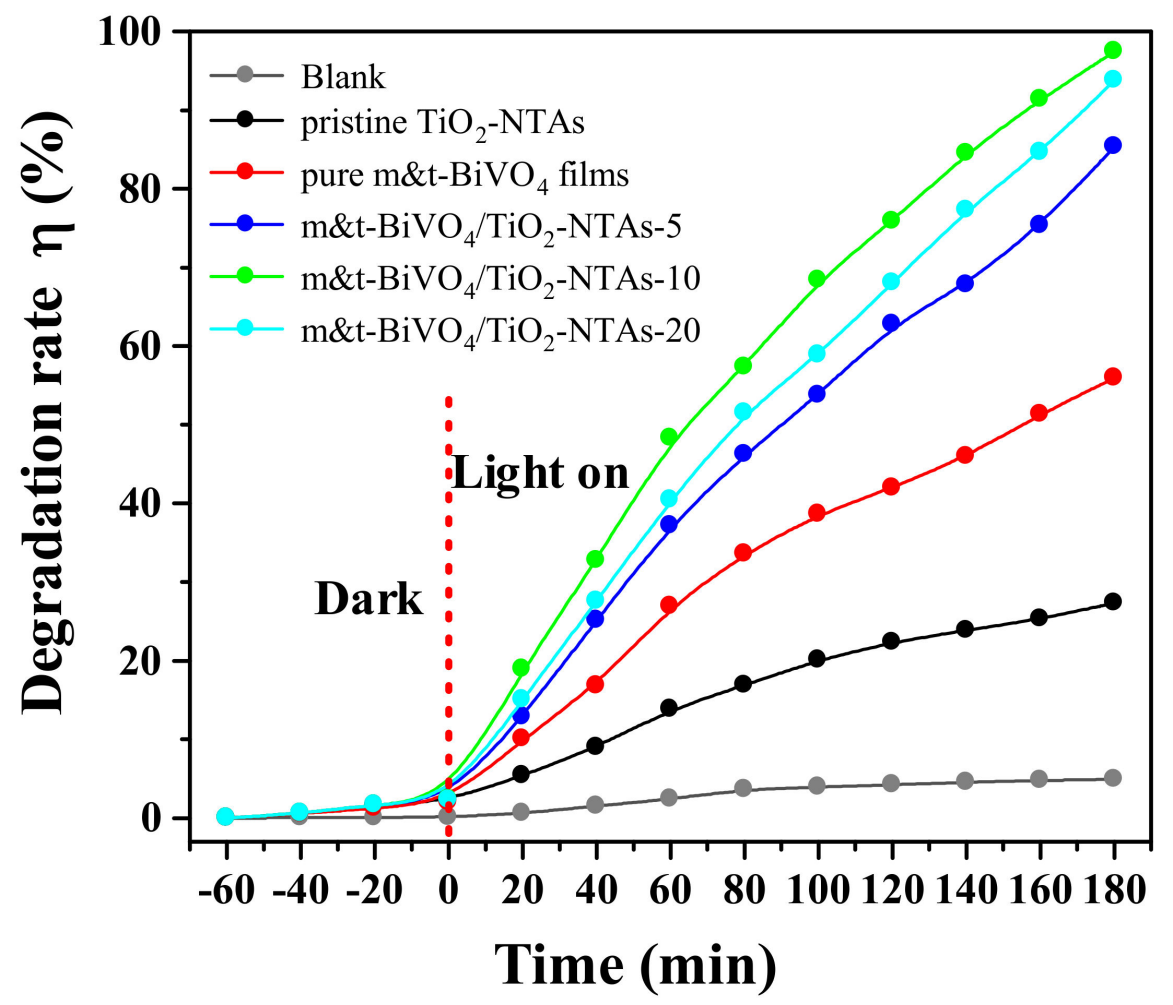
Adsorption process and photodegradation rate (η) of blank test, pristine TiO_2_-NTAs, pure BiVO_4_ films, and m&t-BiVO_4_/TiO_2_-NTAs with various BiVO_4_ amounts (5 h, 10 h, and 20 h) under conditions of dark and UV-Visible light irradiation, respectively.

We conducted the UV–visible photodegradation performance inspections for the TiO_2_-NTAs-based heterostructure nanohybrids irradiated by a standard solar simulated light source. We positioned the as-prepared samples at the center of self-constructed reaction container using double-sided tape, aligning the MO dye adsorbed face upwards and towards the lamp. Initially, we performed the self-degradation test of the MO, aiming to eliminate the influence of the photobleaching effect. Thus, we present the adsorption process and photodegradation efficiency (η) results for the intrinsic self-decomposition of the MO, pristine TiO_2_ NTAs, plain BiVO_4_ films, and BiVO_4_/TiO_2_-NTAs nano-heterojunctions with different BiVO_4_ NP hydrothermal deposition amounts, and with and without UV–visible lamp irradiation (photon-flux of 77.5 W/m^2^) for 180 min, respectively, in Figure 16. We detected the degraded MO solutions at 20 min intervals to calculate the dye concentration as per the equation [118]: η = (C_i_ − C_f_)/C_i_ × 100%, where C_i_ and C_f_ are the initial and final concentrations of the MO solution after irradiation, respectively. The self-photodegradation of MO is not significant (less than 5%). In addition, the samples of pristine TiO_2_-NTAs and pure BiVO_4_ films exhibited less photodegradation activity (27% and 56%, respectively), compared with that of the BiVO_4_/TiO_2_-NTAs nanohybrids under UV–visible lamp irradiation, which was mainly ascribed to the inferior capability of the light absorption in the UV-visible region and the higher reduction potential position for the CB. Apparently, the m&t-BiVO_4_/TiO_2_-NTAs nano-heterojunctions manifested more elevated photodegradation performances for the MO dye than the single TiO_2_ and BiVO_4_ semiconductors due to the cooperative effect of the prolongated light-absorption scope and staggered energy-band structure of the type-II heterostructure, which can acquire more energetic carriers to participate in the oxidation-reduction reaction. Notably, the photodegradation characteristics for the m&t-BiVO_4_/TiO_2_-NTAs specimens were enhanced from ca. 85% to ca. 97% with the BiVO_4_ hydrothermal synthesized time increase from 5 h to 10 h, while the deposition time of the BiVO_4_ further increased to 20 h, the η of which decreased to approximately 93%, which provides convincing evidence that the upgraded CT rate may be slightly more dominant than the carrier lifetime.

**Figure 17:**
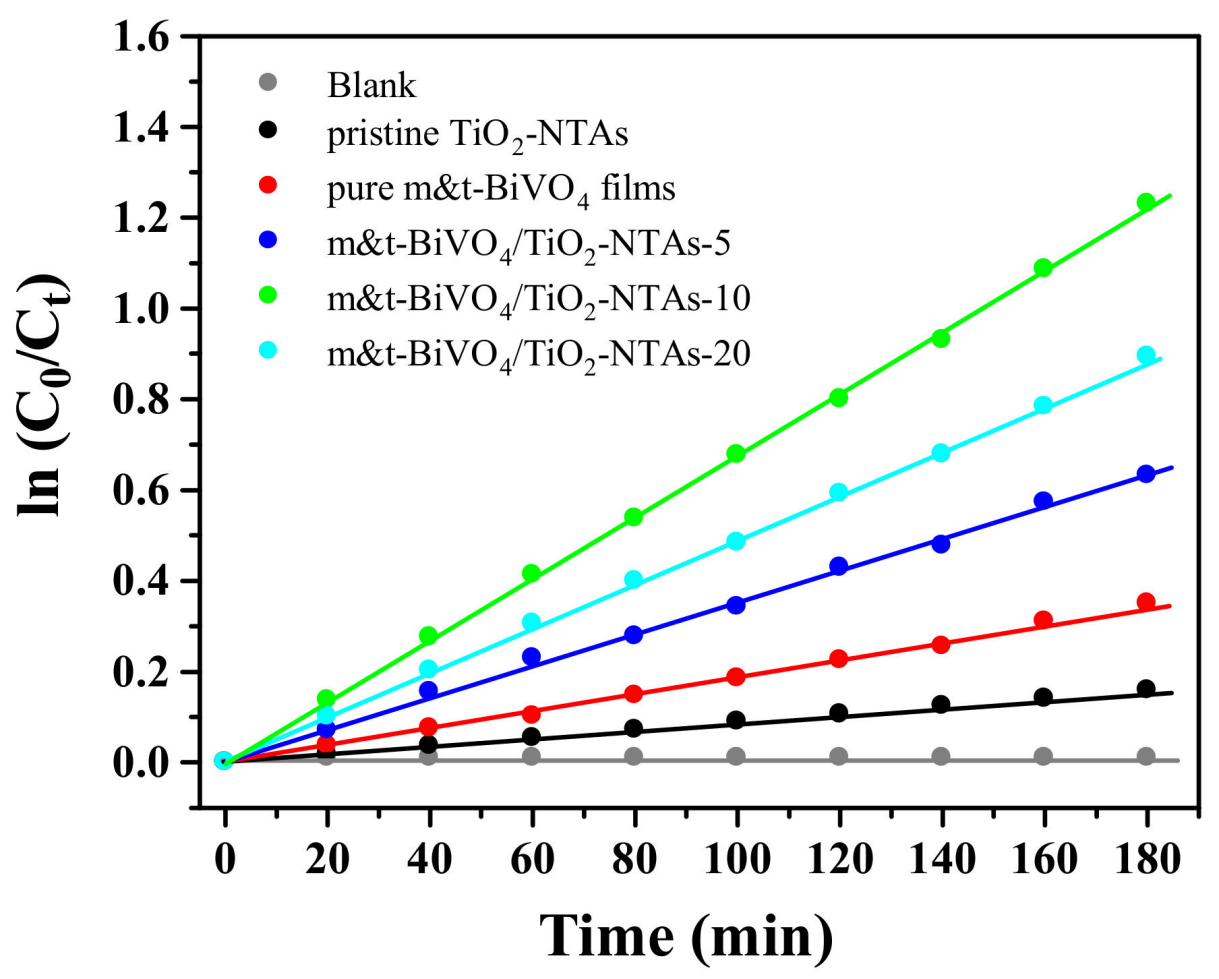
Pseudo-first-order kinetic analysis toward to MO degradation for blank test, pristine TiO_2_-NTAs, pure BiVO_4_ films, and BiVO_4_/TiO_2_-NTAs with different BiVO_4_ deposited times under UV-Visible light irradiation, respectively.

We used the pseudo-first-order kinetic model to quantitatively study the reaction dynamics. This model assumes that ln(C_0_/C_t_) = kt [119], where k is the reaction rate constant, C_0_ is the initial concentration of the reactant, and C_t_ is the concentration of the reactant at time (t). In Figure 17, we explicitly show that m&t-BiVO_4_/TiO_2_-NTAs-10 has the maximum k value, which indicates the optimum photodegradation activity among the as-formed nano-heterojunctions.

**Figure 18:**
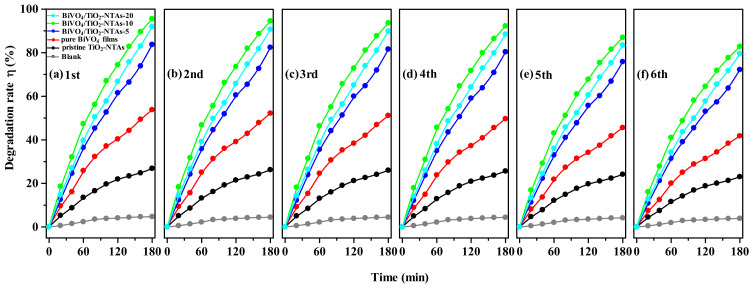
Cyclic photodegradation tests of the blank test, pristine TiO_2_-NTAs, pure BiVO_4_ films, and BiVO_4_/TiO_2_-NTAs with different BiVO_4_ deposited amounts (5 h, 10 h, and 20 h) under UV-Visible light irradiation, (**a**) 1st, (**b**) 2nd, (**c**) 3rd, (**d**) 4th, (**e**) 5th, (**f**) 6th, respectively.

Besides the degradation efficiency, the stability and usability of a photocatalyst are also critical factors that affect its feasibility. We successively conducted cyclic photodegradation tests of the TiO_2_-NTAs-based nano-heterostructures under the same circumstances six times, as depicted in Figure 18.

According to the results of the six cyclic tests, the degradation activities of the as-formed nanohybrids had a slight decrease, as expected, which was mainly ascribed to the inescapable weight loss, which only approached 15% for the maximal deterioration of the photodegradation performance. This highlights the fact that the as-obtained m&t-BiVO_4_/TiO_2_-NTAs nano-heterojunctions had comparatively excellent photodegradation stabilities.

**Figure 19:**
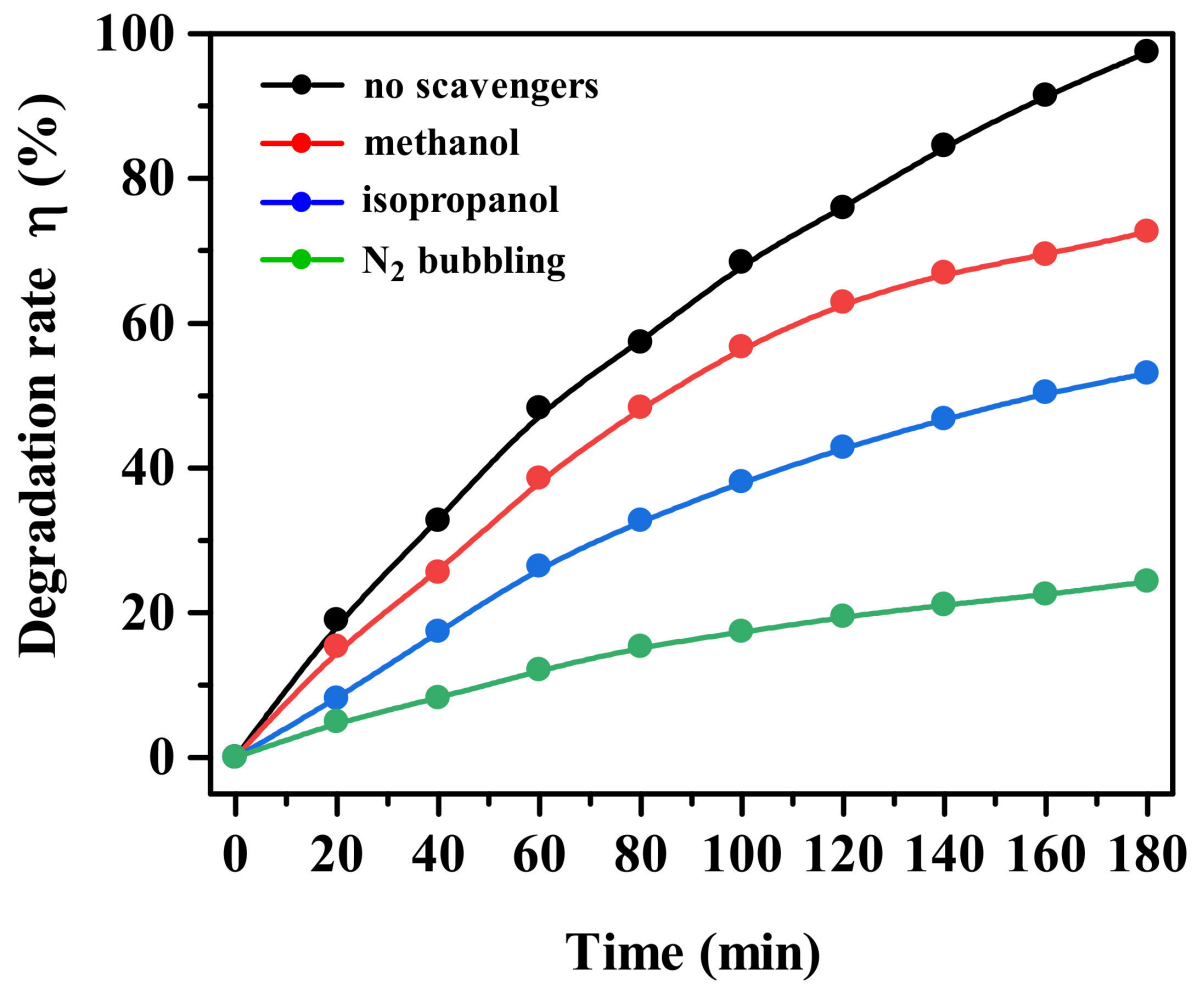
Photodegradation rate η of MO dye for the sample of m&t-BiVO_4_/TiO_2_-NTAs-10 without and with the presence of various scavengers irradiated by UV-Visible light, respectively.

The purpose of the free-radical-trapping experiments was to determine which substances were reactive, such as h^+^, OH, and O_2_^−^, as well as which of them undertakes the primary role for the photodegradation towards MO dye, as exhibited in Figure 19.

The η value of m&t-BiVO_4_/TiO_2_-NTAs-10 was ca. 97% without radical scavengers, and the η values were ca. 73% and ca. 53% with methanol and IPA, respectively. Furthermore, we continuously injected the high-purity N_2_ throughout the degradation reaction, with the aim of eliminating the dissolved O_2_ and inhibiting the generation of O_2^−^_. The removal rate for the MO was only ca. 24%, compared with the 97% under normal atmospheric conditions. Thus, both the OH and O_2^−^_ radical groups are the collective reactive species involved in the degradation process, and O_2^−^_ especially plays a dominant role in the reaction, which is strongly dependent on the number of V_o_ defects.

**Figure 20:**
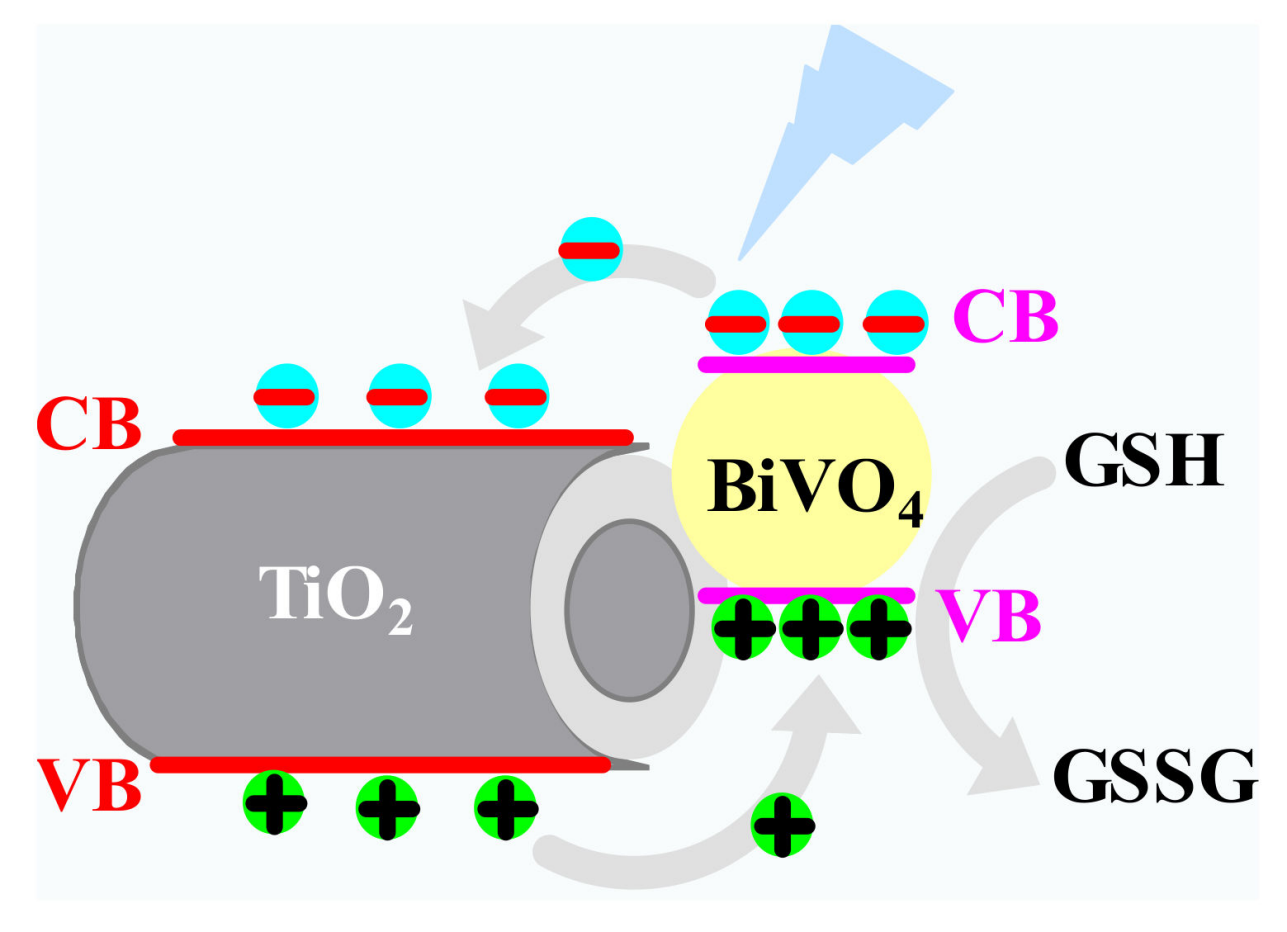
Mechanism diagram of the photoelectrochemical biosensing for GSH detection.

The oxidase-mimicking ability of BiVO_4_/TiO_2_-NTAs nanohybrids makes them an optimal choice of biosensing platforms for accurately determining the GSH levels. We present the GSH detection mechanism of the m&t-BiVO_4_/TiO_2_-NTAs nano-heterojunctions in Figure 20. Under the excitation of simulated solar light, BiVO_4_ and TiO_2_ simultaneously absorb photons to generate e^−^_CB_–h^+^_VB_ pairs. Owing to the existence of the stepped-energy-band heterostructure, photoinduced e^−^_CB_ can quickly transfer from the CB of BiVO_4_ to the CB of TiO_2_, and then transfer to the external circuit. At the same time, the photoexcited h^+^_VB_ migrate from the VB of TiO_2_ to the VB of BiVO_4_, driven by the force of the built-in electric field between BiVO_4_ and TiO_2_. The direction of the built-in electric field is the same as that of the applied positive bias (0.5 V vs. Ag/AgCl), pointing from the TiO_2_ to the BiVO_4_. During the CT process, GSH can be oxidized to glutathione disulfide (GSSG), trapped by the holes in the VB of BiVO_4_ and restraining the rapid recombination of e^−^–h^+^ pairs, which result in the substantial promotion of the photocurrent response compared with the transient I–t tests in Figure 8a. Therefore, the relationship between the GSH concentration and amplified photocurrent effect forms the basis of the biosensing function.

**Figure 21:**
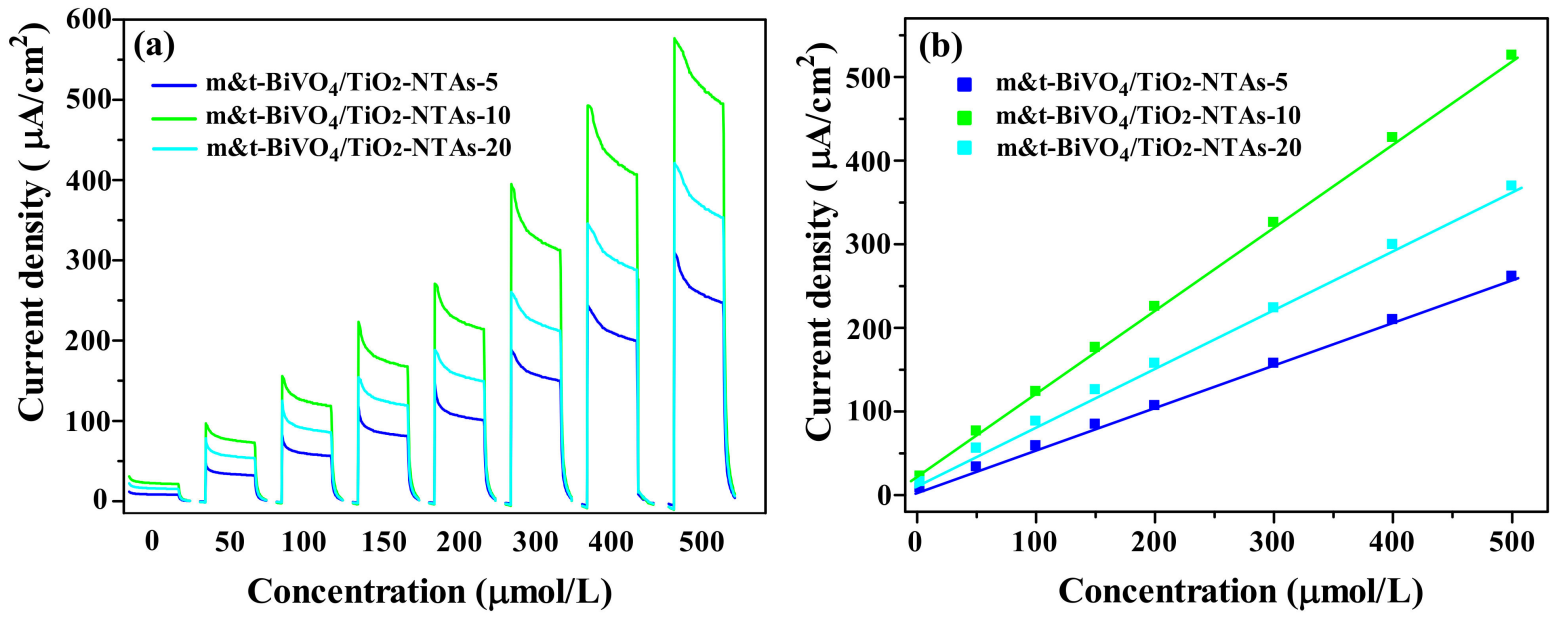
(**a**) Photocurrent performance of the m&t-BiVO_4_/TiO_2_-NTAs type-II heterostructure nanocomposites with various BiVO_4_ NPs hydrothermal-deposited times in the presence of 0, 50, 100, 150, 200, 300, 400, and 500 μM GSH (from left to right) in 0.1 M PBS solution (pH 7.0) at the potential of 0.5 V irradiated by simulated sunlight, respectively; (**b**) the plots of m&t-BiVO_4_/TiO_2_-NTAs heterojunction nanoelectrodes with various BiVO_4_ NPs deposition times 5 h, 10 h, and 20 h with linear relationship toward photocurrent density and concentration of GSH, respectively.

**Figure 22:**
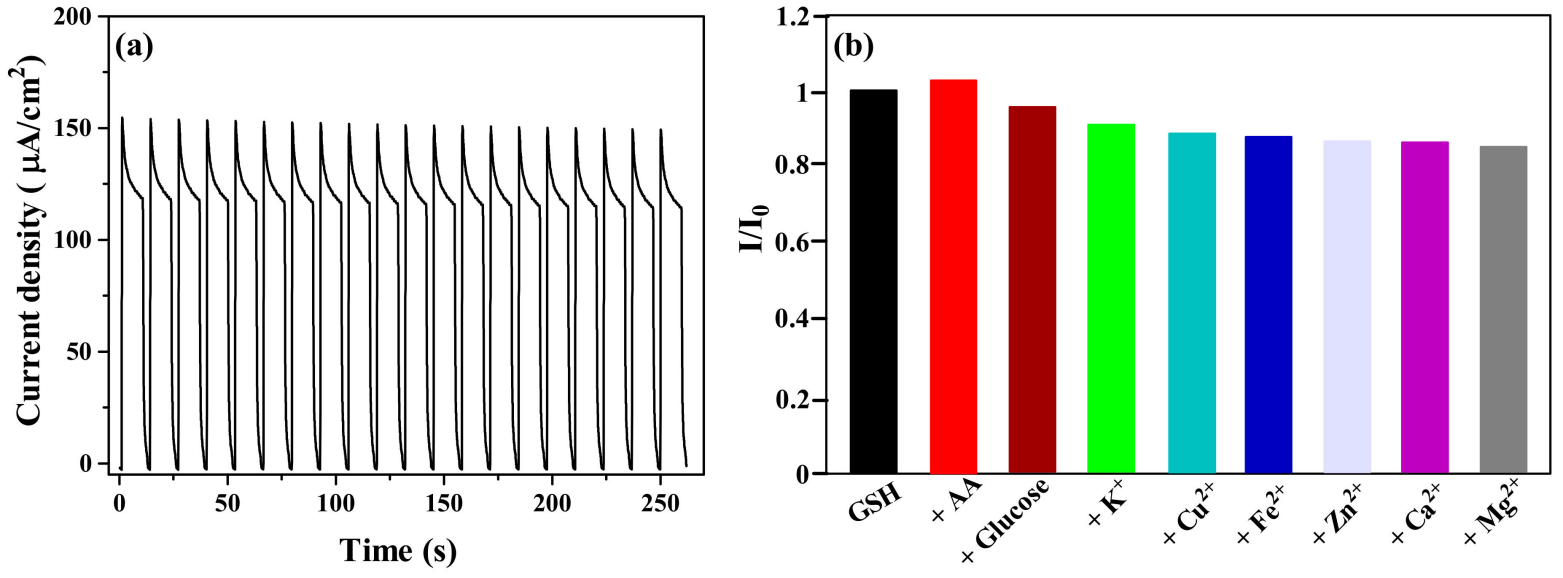
(**a**) Time-based photocurrent performance for the sample of m&t-BiVO_4_/TiO_2_-NTAs-10 irradiated by several cycles in 0.1 M PBS solution containing 100 μM GSH at the potential of 0.5 V (vs. Ag/AgCl) under simulated sunlight irradiation; (**b**) photocurrent ratio I/I_0_ of the m&t-BiVO_4_/TiO_2_-NTAs-10 electrode in 0.1 M PBS solution containing 200 μM GSH with the successive addition of different interfering substances (the concentration of all other interfering species is 200 μM).

We quantitatively tested the constructed m&t-BiVO_4_/TiO_2_-NTAs nano-heterojunctions for PEC biosensing in GSH solutions of various concentrations (0 μM–500 μM), and we recorded the concentration-current curves in 0.1 M PBS solution (pH 7.0) at the potential of 0.5 V (vs. Ag/AgCl) under simulated sunlight irradiation, as exhibited in Figure 21a. The photocurrent responses of the as-synthesized specimens gradually increased with the increase in the GSH concentration, and the photocurrent density of m&t-BiVO_4_/TiO_2_-NTAs-10 was substantially higher than that of the m&t-BiVO_4_/TiO_2_-NTAs-5 and m&t-BiVO_4_/TiO_2_-NTAs-20 with the increase in the GSH concentration, confirming that the former has a superior photoinduced carrier CT efficiency and separation ability to the others, which is mainly due to the greater values of the ΔE_c_ and ΔE_v_ for m&t-BiVO_4_/TiO_2_-NTAs-10 mediated by the synergistic effect. In addition, the photocurrent response of m&t-BiVO_4_/TiO_2_-NTAs-10 had an excellent linear relationship with the GSH concentration (R^2^ = 0.9889), with a linear range from 0 to 500 μM, as shown in Figure 22b. This upper detection limit is more pertinent to detecting GSH in biological specimens because the cellular GSH concentration is at mM levels [120]. Simultaneously, the PEC biosensing performance for m&t-BiVO_4_/TiO_2_-NTAs-10 showed a limit of detection (LOD) of 2.6 μM (a signal-to-noise ratio of 3), with a sensitivity of 960 mA cm^−2^ M^−1^, which was 1.92-fold and 1.38-fold greater than those for the m&t-BiVO_4_/TiO_2_-NTAs-5 and m&t-BiVO_4_/TiO_2_-NTAs-20 specimens, respectively.

We review the analysis performance of the GSH using m&t-BiVO_4_/TiO_2_-NTAs heterostructure nanohybrids in this work and other modified materials found in the literature in Table 8. The linear response range was wider than those of the colorimetric biosensing, fluorescence biosensor, and other PEC methods. The m&t-BiVO_4_/TiO_2_ NTAs also displayed a lower detection limit for GSH compared with the fluorimetry and other PEC methods. Most important of all, the proposed BiVO_4_/TiO_2_-NTAs nano-heterostructure PEC biosensing approach is characterized by excellent stability and selectivity.

To be effective, PEC biosensors should possess good stability and selectivity. We chose the m&t-BiVO_4_/TiO_2_-NTAs-10 specimen as the candidate for the stability and selectivity testing, as it has the optimal PEC activity among all the as-fabricated nanohybrids. We evaluated the photoexcited biosensing stability of the selected sample by measuring the time-based photocurrent response under several on/off irradiation cycles in a 0.1 M PBS solution containing 100 μM GSH at a potential of 0.5 V (vs. Ag/AgCl), irradiated by simulated sunlight irradiation. Within 260 s, the detection process of the nano-heterojunction had cycled 20 times, and as exhibited in Figure 22a, there was almost no decay on the photocurrent and 96.5% of its initial value was retained, which demonstrates that the BiVO_4_/TiO_2_-NTAs electrode had the desirable stability in the GSH detection. For probing the selectivity of the constructed nano-heterostructure photoelectrode, we adopted the ratio of the photocurrent intensity (I/I_0_) to characterize the effects of a series of interfering substances on the photocurrent. I and I_0_ represent the photocurrents before and after the addition of other interferents, respectively. For the characterization, we used metal ions (K^+^, Cu^2+^, Fe^2+^, Zn^2+^, Ca^2+^, and Mg^2+^), glucose, and ascorbic acid (AA). As displayed in Figure 22b, we did not observe any salient photocurrent variation with the successive addition of 200 μM AA, glucose, and other metal ions into the electrolyte containing 200 μM GSH. Among them, AA is a good electron donor and can be photocatalytically oxidized by the as-prepared nano-heterojunctions, and AA also caused the photocurrent to slightly increase, but it had little effect on the experimental results. Eventually, we verified the biosensing stability of the m&t-BiVO_4_/TiO_2_-NTAs photoelectrode via intermittent photocurrent response tests.

**Figure 23:**
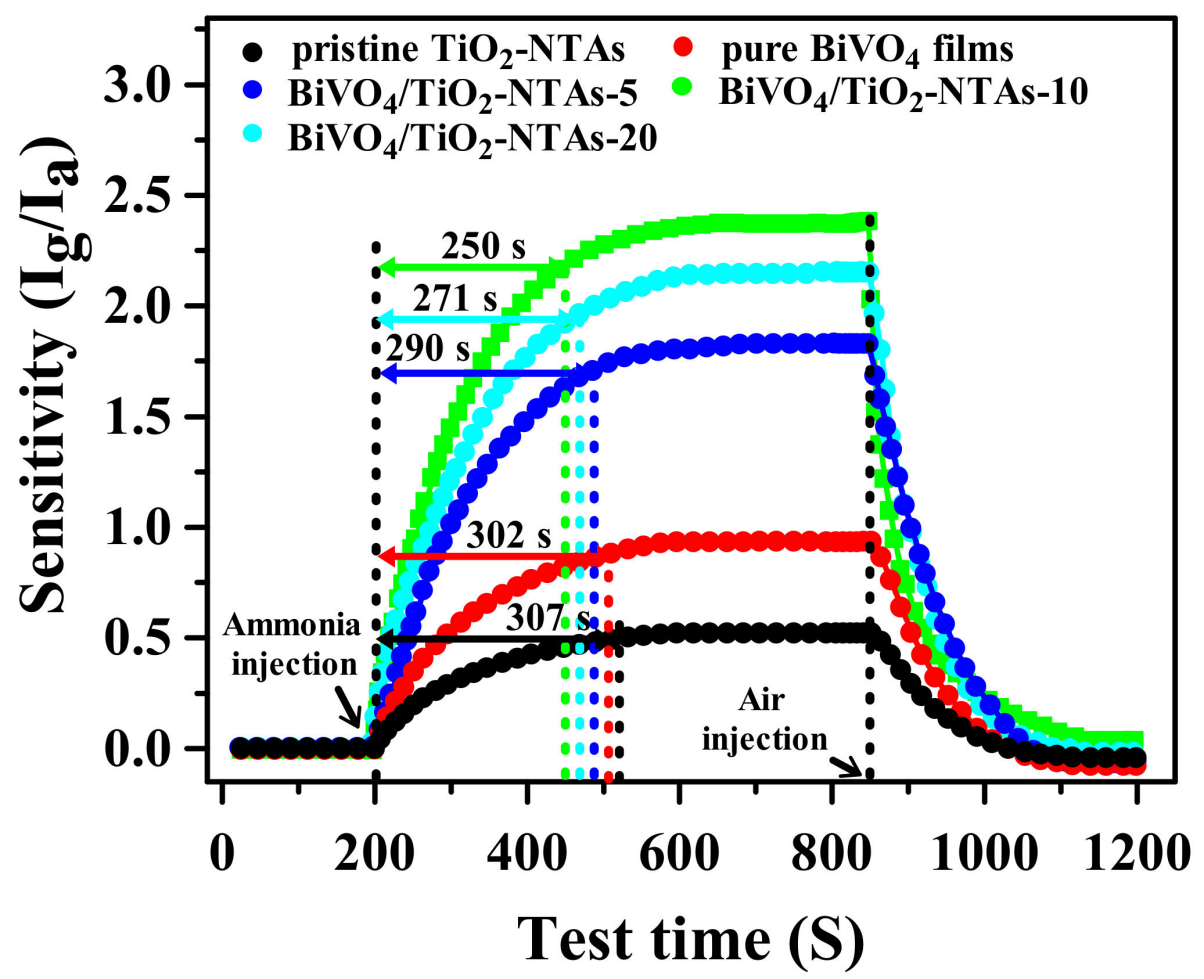
Sensitivity of pristine TiO_2_-NTAs, pure BiVO_4_ films and m&t-BiVO_4_/TiO_2_-NTAs nanohybrids with different BiVO_4_ NPs depositing amounts as a function of time, respectively.

We present the graphical sensitivity vs. time relations of the as-prepared specimens at room temperature in Figure 23. The parameter sensitivity (S) for gas-sensing can be defined as follows [126]: S = I_g_/I_a_, where I_g_ is the experimentally recorded stable current values during the targeted gas flow, and I_a_ is the recorded stable current during the air gas flow. The response time (τ_res_) is defined as the time to reach 90% of the final equilibrium value. When the NH_3_ permeation reached t = 200 s, the S values were exponentially increased, as expected, while they were exponentially decreased when the air injection reached t = 850 s. Simultaneously, there were smaller S values (ca. 0.5 and 0.8) for the gas-sensing of the pristine TiO_2_ NTA and pure BiVO_4_ film sensors owing to their larger electronic transfer impedances and narrower light-absorption scopes, which led to lower photoexcited current responses. The S value increased from ca. 1.8 to ca. 2.4 with the increase in the BiVO_4_ hydrothermal deposition times from 5 h to 10 h; however, the S value decreased to ca. 2.2 with the increase in the of BiVO_4_ preparation time to 20 h, which are consistent with the abovementioned observed trends in the photodegradation and PEC biosensing. In addition, the τ_res_ values for the pristine TiO_2_ NTAs and plain BiVO_4_ films were 307 s and 302 s, respectively. As a comparison, the τ_res_ of the m&t-BiVO_4_/TiO_2_-NTAs-5 sample was about 290 s towards the sensing of NH_3_ gas, while those for the m&t-BiVO_4_/TiO_2_-NTAs-10 and m&t-BiVO_4_/TiO_2_-NTAs-20 samples were around 250 s and 271 s, respectively. The gas-sensing performances of the sensitivity and response speed for the m&t-BiVO_4_/TiO_2_-NTAs nano-heterostructure are superior to those of the individual BiVO_4_ and TiO_2_ semiconductors. The specimen of m&t-BiVO_4_/TiO_2_-NTAs-10, especially, is the ideal platform for gas-sensing, possessing a higher sensitivity and faster response speed in comparison with those of the m&t-BiVO_4_/TiO_2_-NTAs-5 and m&t-BiVO_4_/TiO_2_-NTAs-20.

Combined with the obtained results, we propose a reasonable theory to explain the gas-sensing mechanism toward NH_3_. The conductivity of nano-heterostructures is proportional to the concentration of conducting electrons. In the beginning, the atmospheric O_2_ can be converted to O_2_^−^, attaching to the active sites of the V_o_ defects in nano-heterojunctions, which leads to a reduced concentration of carriers. With the injection of NH_3_ gas, reductive NH_3_ molecules can react with O_2_^−^ (i.e., 4NH_3_ (gas) + 3 O_2_^−^ (adsorption) → 6H_2_O (gas) + 2N_2_ (gas) + 3e^−^), liberating the electrons as free charges and increasing the conductivity of the nano-heterostructures, and the electrically neutral N_2_ gas will be released back to the ambience. The increased concentration of e^−^_CB_ due to the exposure of the hetero-nanosystem to NH_3_ gas is a result of the electron-donating properties of analyte gas. Specifically, the m&t-BiVO_4_/TiO_2_-NTAs heterostructure nanocomposites can inject redundant electrons into the CB of TiO_2_ from the CB of BiVO_4_ irradiated by UV–vis light, which facilitates the formation of O_2_^−^. The m&t-BiVO_4_/TiO_2_-NTAs-10 sample manifested the best performances for photodegradation and gas-sensing, which are tightly associated with the O_2_^−^ concentration, which is mainly attributed to the number of V_o_ defect active sites and the superior capacity of CT associated with the greater ΔE_c_ and ΔE_v_ values.

## 4. Conclusions

In conclusion, we constructed the m&t-BiVO_4_/TiO_2_-NTAs type-II nano-heterojunctions via m&t-BiVO_4_ NPs integrated with the ordered arrangement of TiO_2_ NTAs using the low-cost hydrothermal-deposition approach. The as-synthesized m&t-BiVO_4_/TiO_2_-NTAs nanohybrids exhibited dramatically improved photodegradation, PEC biosensing, and NH_3_ gas-sensing performances compared with the single semiconductor under UV–visible irradiation, as expected, which is consistent with the variation trend of the PEC activity tests, which is mainly ascribed to the positive synergistic effect between the content ratio of the m&t-BiVO_4_ related to the hydrothermal preparation time and the number of V_o_ defects mediated by the pH value, which induce the uplifted band offset and promote the exposed reaction active sites related to V_o_ defects. We verified the deduction by the probing results of the NTRT-PL and TRPL spectra, correspondingly proposing semi-qualitative and semi-quantitative analyses for the interfacial CT dynamics process, which demonstrates the promotion of the separation of the photoinduced e^−^–h^+^ pairs and elevated charge injection efficiency for the as-obtained nano-heterojunctions. Thus, it is expectable that the m&t-BiVO_4_/TiO_2_-NTAs nano-heterojunctions not only provide in-depth comprehension for the interfacial CT process between different photocatalysts, but also contributes new insight into the design of devices for PEC biosensing and NH_3_ gas-sensing with superior performances.

## Figures and Tables

**Table 1:** The percentage composition of tetragonal and monoclinic phases of BiVO_4_ in pure BiVO_4_ and m&t-BiVO_4_/TiO_2_-NTAs nanoheterojunctions (determined by Equations (1) and (2)) under different pH values of precursor.

as-Formed Samples	pH Value of Precursor	ms-BiVO_4_ (η_ms-B/T_, %)	tz-BiVO_4_ (η_tz-B/T_, %)
pristine BiVO_4_ films	5	46.7	53.3
BiVO_4_/TiO_2_-NTAs-5	2	36.8	63.2
BiVO_4_/TiO_2_-NTAs-10	5	48.4	51.6
BiVO_4_/TiO_2_-NTAs-20	8	71.9	28.1

**Table 2:** Comparative data of E_g_ for Tauc and E_g-W_ for weighted contents towards m&t-BiVO_4_/TiO_2_-NTAs dual nanohybrids with different fabrication conditions.

as-Prepared Samples	Calculated E_g_ for Tauc (eV)	Predicted E_g-W_ (eV)
m&t-BiVO_4_/TiO_2_-NTAs-5	2.65	2.66
m&t-BiVO_4_/TiO_2_-NTAs-10	2.58	2.59
m&t-BiVO_4_/TiO_2_-NTAs-20	2.52	2.54

**Table 3:** Surface atomic ratios Ti^3+^/Ti^4+^ of Ti 2p XPS spectra for spin-orbit splitting doublets Ti 2p_1/2_ and Ti 2p_3/2_ towards to the samples of pristine TiO_2_-NTAs and BiVO_4_/TiO_2_-NTAs-10, respectively.

as-Prepared Samples	Assignment	Binding Energy (eV)	Surface Atomic Ratio Ti^3+^/Ti^4+^
pristine TiO_2_-NTAs	Ti 2p_3/2_	458.5	0.543
	Ti^3+^ 2p_3/2_	458.2	
	Ti^4+^ 2p_3/2_	458.7	
	Ti 2p_1/2_	464.2	
	Ti^3+^ 2p_1/2_	463.9	
	Ti^4+^ 2p_1/2_	464.5	
m&t-BiVO_4_/TiO_2_-NTAs-10	Ti 2p_3/2_	458.2	0.988
	Ti^3+^ 2p_3/2_	458.1	
	Ti^4+^ 2p_3/2_	458.8	
	Ti 2p_1/2_	463.8	
	Ti^3+^ 2p_1/2_	463.6	
	Ti^4+^ 2p_1/2_	464.8	

**Table 4:** Surface molar ratios V^4+^/V^5+^ of V 2p XPS spectra for spin-orbit splitting doublets V 2p_1/2_ and V 2p_3/2_ as to the specimens of m&t-BiVO_4_/TiO_2_-NTAs with different hydrothermal synthesis times, respectively.

as-Prepared Samples	Assignment	Binding Energy (eV)	Surface Atomic Ratio V^4+^/V^5+^
m&t-BiVO_4_/TiO_2_-NTAs-5	V^4+^ 2p_3/2_	516.2	0.436
	V^5+^ 2p_3/2_	516.5	
	V^4+^ 2p_1/2_	523.4	
	V^5+^ 2p_1/2_	524.1	
m&t-BiVO_4_/TiO_2_-NTAs-10	V^4+^ 2p_3/2_	515.5	0.587
	V^5+^ 2p_3/2_	516.3	
	V^4+^ 2p_1/2_	523.1	
	V^5+^ 2p_1/2_	523.8	
m&t-BiVO_4_/TiO_2_-NTAs-20	V^4+^ 2p_3/2_	515.8	0.491
	V^5+^ 2p_3/2_	516.4	
	V^4+^ 2p_1/2_	523.3	
	V^5+^ 2p_1/2_	524.0	

**Table 5:** Surface V_o_/(L_o_ + A_o_), and A_o_/(L_o_ + V_o_) molar ratios of O 1s XPS spectra for the specimens of m&t-BiVO_4_/TiO_2_-NTAs with different hydrothermal synthesis times (5 h, 10 h, and 20 h), respectively.

as-Prepared Samples	Assignment	Binding Energy (eV)	V_o_/(L_o_ + A_o_), and A_o_/(L_o_ + V_o_)
m&t-BiVO_4_/TiO_2_-NTAs-5	L_o_	529.9	0.361 and 0.336
	V_o_	530.5	
	A_o_	531.3	
m&t-BiVO_4_/TiO_2_-NTAs-10	L_o_	529.9	0.571 and 0.423
	V_o_	531.1	
	A_o_	531.3	
m&t-BiVO_4_/TiO_2_-NTAs-20	L_o_	530.0	0.402 and 0.396
	V_o_	530.5	
	A_o_	531.7	

**Table 6:** The density of donor carriers (N_d_), flat band potential (E_fb_), and position of CB for pure TiO_2_-NTAs, pristine m&t-BiVO_4_ films, and m&t-BiVO_4_/TiO_2_-NTAs nanocomplex with BiVO_4_ depositing times 5 h, 10 h, and 20 h, respectively.

Samples	N_d_ (cm^−3^)	E_fb_ (vs. NHE)	CB Position (vs. NHE)
pristine TiO_2_-NTAs	6.2 × 10^17^	−0.175	−0.275
Pure BiVO_4_ films	3.3 × 10^18^	0.377	0.277
m&t-BiVO_4_/TiO_2_-NTAs-5	4.5 × 10^18^	−0.329	−0.429
m&t-BiVO_4_/TiO_2_-NTAs-10	7.6 × 10^18^	−0.428	−0.528
m&t-BiVO_4_/TiO_2_-NTAs-20	6.6 × 10^18^	−0.364	−0.464

**Table 7:** Average lifetime of PL (τ_avg_) for pristine TiO_2_-NTAs and BiVO_4_/TiO_2_-NTAs nanoheterojunctions with different BiVO_4_ hydrothermal-depositing times 5 h, 10 h, and 20 h, respectively.

Sample Codes	λ_ex_ (nm)	λ_em_ (eV)	τ_1_ (ns)	A_1_/(A_1_ + A_2_) (%)	τ_2_ (ns)	A_2_/(A_1_ + A_2_) (%)	τ_avg_ (ns)
pristine TiO_2_-NTAs	375	2.8	2.33	54.0	6.16	46.0	4.99
plain BiVO_4_ films	375	1.8	2.38	52.8	5.56	47.2	4.53
m&t-BiVO_4_/TiO_2_-NTAs-5	375	2.8	2.35	60.1	5.53	39.9	4.29
m&t-BiVO_4_/TiO_2_-NTAs-10	375	2.8	2.21	42.8	4.47	57.2	3.86
m&t-BiVO_4_/TiO_2_-NTAs-20	375	2.8	2.31	56.5	5.09	43.5	4.06

**Table 8:** Linear range and limit of detection (LOD) comparison of various methods for detecting GSH.

Sensor Type	Sensing Method	Linear Range (μM)	LOD (μM)	Ref.
BSA-AuNP@ZnCo_2_O_4_	Colorimetric biosensing	0.5–15	0.0885	[121]
CuPd@H-C_3_N_4_	Colorimetric biosensing	2–40	0.58	[122]
In_2_O_3_/In_2_S_3_	PEC biosensing	1–100	0.82	[123]
N, S-Cdots-MnO_2_	fluorimetry	0–250	28.5	[124]
Bi_2_S_3_/TiO_2_-NTAs	PEC biosensing	15–200	7	[125]
m&t-BiVO_4_/TiO_2_-NTAs	PEC biosensing	0–500	2.6	This work
